# Impact of insecticide resistance evolution on malaria vector control

**DOI:** 10.1371/journal.pcbi.1014612

**Published:** 2026-08-03

**Authors:** Neil Philip Hobbs, Sumin Kim, Thiery Masserey, Nakul Chitnis

**Affiliations:** 1 Department of Epidemiology and Public Health, Swiss Tropical and Public Health Institute, Allschwil, Switzerland; 2 University of Basel, Basel, Switzerland; Fundação Getúlio Vargas: Fundacao Getulio Vargas, BRAZIL

## Abstract

**Background:**

Insecticide-treated nets (ITNs) are the staple of malaria vector control. Given their importance, insecticide resistance (IR) is extremely concerning. Understanding how ITNs simultaneously impact the evolution of IR and malaria control is of critical importance, especially with next-generation mixture ITNs (NGM-ITNs) becoming available.

**Method:**

We developed a mosquito population dynamics and genetics model to explore the simultaneous impact of ITNs on both malaria transmission (measured as vectorial capacity, an entomological measure of transmission) and IR evolution (resistance gene frequencies), and their interactions. We explored the long-term impact of interventions often neglected in other modelling exercises. We conducted three sets of simulations and sensitivity analyses. The first set investigated the impact of pyrethroid-only ITNs (PYR-ITNs) on IR evolution and its consequent impact on malaria vector control. The second set investigated the dual impact of NGM-ITNs in controlling malaria transmission and mitigating the evolution of resistance: IR management (IRM). The third set further considered net retention and insecticide decay.

**Results:**

For both ITN types, higher ITN coverage provided greater malaria vector control than lower coverage (of the same ITN type), even when considering IR evolution. In addition, NGM-ITNs were superior to PYR-ITNs for both malaria vector control and IRM. Even when NGM-ITNs were deployed at reduced coverage (up to 30%) than PYR-ITNs (to account for their higher procurement costs), their transmission control efficacy remains superior to PYR-ITNs by providing an additional IRM benefit through reduced selection pressures. Moreover, we found that insecticide selection on male mosquitoes may be an important consideration for malaria vector control loss. While male mosquitoes do not contribute to transmission, they propagate resistance genes, and their importance in this process is a knowledge gap.

**Discussion:**

Our results highlight the need to consider IRM when evaluating NGM-ITNs, which is not adequately considered in the evaluation pipeline. Not accounting for IRM means new interventions are being undervalued, as a component of their long-term effectiveness is being overlooked.

## Author summary

Malaria vector control is reliant on insecticides primarily deployed as insecticide treated nets. To counter the threat of resistance, the use of insecticide resistance management is recommended to slow the spread of resistance. We developed a model which integrates insecticide resistance genetics and mosquito population dynamics to allow us to simultaneously explore how different malaria vector control options impact both transmission and resistance. Using parameter space testing and sensitivity analysis, we identified the surprising role of male mosquitoes as key in accelerating resistance spread (which compromises vector control) without providing any vector control benefit. In exploring the role of next-generation mixture insecticide treated nets we found these to be better at managing resistance than deploying single insecticide nets. Given next-generation mixture nets are more expensive, we further considered the implication of reduced coverage and how this impacts both resistance dynamics and transmission dynamics. Lowering coverage further reduced insecticide selection pressures extending the usable lifespan of next-generation mixture nets, while maintaining equivalent or better transmission than deploying higher coverage pyrethroid-only insecticide treated nets.

## Introduction

Interventions targeting *Anopheles* mosquitoes to prevent malaria transmission have played a critical role in reducing the burden of malaria, especially those in the form of insecticide-treated nets (ITNs) and indoor residual spraying (IRS) [1]. Both interventions rely on insecticides and their use contributed to the evolution of insecticide resistance (IR), which concerningly threatens malaria vector control [2,3].

Over recent years, there has been a plateauing in the prevalence of malaria globally [4], with IR being identified as a contributing factor. The pyrethroid insecticide class which, until recently, has been the only class suitable for use on ITNs, has widespread IR [5]. In response, the WHO developed the Global Plan for Insecticide Resistance Management (GPIRM) [6], which suggested insecticide mixtures for use when they become available as a potential insecticide resistance management (IRM) strategy (defined as a strategy delaying the evolution of IR). Next-generation mixture ITNs (NGM-ITNs), containing pyrethroids and new insecticides, are becoming available, following successful evaluation in epidemiological field trials [7,8]. However, NGM-ITNs still contain pyrethroid, which may have consequences for IRM.

Understanding how to deploy new insecticidal interventions in ways which maximise malaria vector control while minimising the selection for IR is of crucial importance. Evaluating the IRM impact of insecticidal interventions is challenging during field trials. Where resistance monitoring is done, this is often insufficient to fully capture the long-term dynamics of IR [9]. Much of the guidance regarding IRM is therefore underpinned by mathematical modelling [10–15].

Previous models have either assessed how IR affects the impact of malaria vector control tools (assuming a static level of IR [16]), while others focused on studying factors that impact the evolution of IR [11,17]. Extending models to allow for the interaction between malaria transmission dynamics and IRM is a clear priority to understand the long-term implications of deploying insecticidal interventions. Here, we developed a mathematical model, “PopGEM”, for integrating mosquito population dynamics with IR gene dynamics. We used PopGEM to model the impact of pyrethroid-only ITNs (PYR-ITNs) and NGM-ITNs on the evolution of IR and malaria vector control over time and identify how to maximise malaria vector control, while minimising IR evolution.

We searched the Web of Science database on 24/06/2025 using the search terms “malaria transmission”, “insecticide resistance management” and “model” OR “simulation” to identify the current available modelling evidence. Most modelling studies focus either on the evolution of IR [10,11,17] or on how IR impacts malaria vector control [16] independently. Some studies integrated both IR evolution and its impact on malaria vector control [18], however, they primarily focused on PYR-ITNs and did not further explore the mechanistic explanation underlying evolutionary-epidemiological processes, preventing them from drawing conclusions on the drivers of both IR and malaria vectorial control. NGM-ITNs containing a mixture of insecticides (a pyrethroid and a novel insecticide) are becoming more widely available. This makes it essential to develop models that integrate IR evolution and malaria transmission dynamics to maximise malaria vector control and minimise the selection for IR.

This novel modelling approach integrates IR evolution with malaria transmission dynamics (measured as vectorial capacity, an entomological measure of transmission), providing a more comprehensive framework than previous models that examined IR evolution in isolation. It improves our understanding of how IR evolution over time changes the effectiveness of ITNs by comparing PYR-ITNs with NGM-ITNs, an emerging intervention now being deployed in the field, but still lacking optimised implementation guidelines. The model also identifies key parameters driving IR and malaria vector control, offering valuable insights for more effective and sustained malaria vector control strategies. The study demonstrated that, for both ITN types, higher ITN coverage provided greater malaria vector control than lower coverage, even in the presence of IR, maintaining high coverage could off-set the impact of resistance on malaria vector control. Additionally, NGM-ITNs consistently outperformed PYR-ITNs in controlling malaria vector, even when deployed at lower coverages (up to 30% reduction), which further improved the IRM impact of NGM-ITNs. The study also highlighted the important role of male mosquitoes in IR evolution, despite their limited anthropophilic behaviour and minimal direct contact with ITNs.

## Methods

### Mathematical model overview

**Fig 1:**
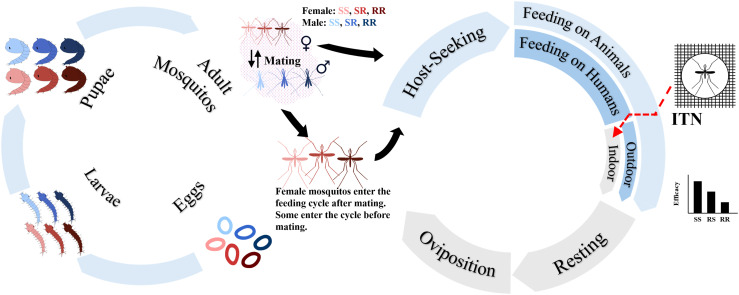
Overview of the PopGEM (Population Genetics and Ecology in Mosquitoes) model. The model simulates the developmental life cycle of mosquitoes, progressing from eggs to adults. The model tracks both male (blue) and female (red) mosquitoes that can mate when they reach the adult stage. Adult female mosquitoes transition to the blood-feeding cycle, either before or after mating. During this phase, females seek blood meals from vertebrate hosts, bloodfeed, rest, and lay eggs (if they managed to mate beforehand). Mosquitoes that are both anthropophilic (feed on humans) and endophagic (feed indoors) are exposed to ITNs dependent on coverage. Mosquito survival after ITN exposure depends on IR genotype. The model assumes resistance to each insecticide is encoded at single loci, with two alleles per loci (either Susceptible (S) or Resistant **(R)**). Mosquitoes can have three different genotypes: SS (light red, light blue), RS (red, blue), and RR (dark red, dark blue). The genotypes dictate ITN efficacy. Note that the killing effect of ITN for each genotype varies within a range in the analysis (see Table 1). For modelling NGM-ITNs, we extended the model to include a second gene, to allow for tracking IR to the second insecticide (see appendix 5).

PopGEM is a deterministic stage structured model and is fully described in appendices 1–5 in S1 File and conceptually shown in Fig 1. Briefly, PopGEM models the life cycle of mosquitoes in daily time-steps, incorporating both aquatic (eggs, larvae, pupae) and adult stages for males and females. The model considers that each mosquito can have a different level of IR, caused by resistance genes encoded at single loci, with two alleles per loci (either Susceptible (S) or Resistant (R)). For modelling mixture interventions, we extend the model to include a second gene, to allow for tracking IR to the second insecticide (see appendix 5). PopGEM models adult female mosquitoes that enter the feeding cycle starting with seeking a host, then they can find a host and feed on it, rest and lay eggs. Mosquitoes can bloodfeed on either animals or humans. Mosquitoes that prefer humans (anthropophagic) and feed indoors (endophagic) may be exposed to ITNs, depending on coverage. The impact of ITNs will depend on the ITN type and the genotype of the mosquito encountering the ITN as described in Fig 1 and Table 1.

**Table 1:** Parameters in sensitivity analysis simulations.

Name	Symbol	Value	Description/Rationale
Male genotype insecticide survival probability	$i_{A}^{\mathrm{\backslash mars}MM}$	$i_{i}^{\mathrm{\backslash venus}FF}(\text{0}\cdot \text{49630446 to }\text{0}\cdot \text{8543562})$	See parameter estimation details in appendix 3 in S1 File).
Genotype blood-feeding probability when ITN present.	$f_{HI|net}^{\mathrm{\backslash venus}FF}$	$\text{1}\;-(\text{exp}(\delta_{\text{4}}(\text{1}-\text{exp}\left(\delta_{\text{5}}i_{i}^{\mathrm{\backslash venus}FF}\right))/\delta_{\text{5}}))$	Calculation from [19], converts experimental hut survival to blood-feeding probability.
Dominance of resistance	${Dominance}_{i}$	0-1	Covers all possible ranges for the dominance, which indicates the RS phenotype in relation the SS and RR phenotypes.
Female survival probability (SS) to ITN containing insecticide i	$i_{i}^{\mathrm{\backslash venus}SS}$	0 – 0·2	Covers plausible range female mosquitoes with SS genotype when exposed to ITNs with insecticide i.
Female survival probability (RS) to ITN containing insecticide i	$i_{i}^{\mathrm{\backslash venus}RS}$	$\left(\left(i_{i}^{\mathrm{\backslash venus}RR}-\;i_{i}^{\mathrm{\backslash venus}SS}\right)*{Dominance}_{i}\right)+\;i_{i}^{\mathrm{\backslash venus}SS}$	Covers plausible range female mosquitoes with RS genotype when exposed to ITNs with insecticide i.
Female survival probability (RR) to ITN containing insecticide i	$i_{i}^{\mathrm{\backslash venus}RR}$	0·4 – 1	Covers plausible range female mosquitoes with RR genotype when exposed to ITNs with insecticide i.
Mating timing preference	Q	0 – 1	Covers full range of potential biological preferences.
Female mating success	$s^{FF}$	0·2 to 1	Covers full range of plausible values.
Probability successful outdoor blood-feeding	$f_{HO}^{\mathrm{\backslash venus}FF}$	0·625 to 0·74	Heavily damaged untreated net [20] and therefore mimics human host defensive behaviours.
Probability success feeding on an animal	$f_{A}^{\mathrm{\backslash venus}FF}$	0·56 to 0·78	Feeding success probability from [21]
Anthropophily	H	0·1 to 1	Covers full range of plausible values for mosquitoes involved in malaria transmission.
Endophagy	Z	0·1 to 1	If values are 0, then the mosquitoes never feed indoors, in which case bed-nets would not be a viable intervention.
Adult female natural survival probability	$\rho_{H}^{\mathrm{\backslash venus}FF},\;\rho_{R}^{\mathrm{\backslash venus}FF},\rho_{O}^{\mathrm{\backslash venus}FF}$	0·66 to 0·95 day^-1^	From 49, with the extremes of the 95% confidence intervals. Values the same irrespective of physiological state.
Adult male natural survival probability	$\rho_{A}^{\mathrm{\backslash mars}MM}$	$\rho_{R}^{\mathrm{\backslash venus}FF}$* 0·75	Average life span of males is 75% of female [22, 23].
ITN Coverage	Y	0·1 to 1	Coverage values accounting for full range of possible scenarios.
Male insecticide encounter probability	$\varepsilon^{\mathrm{\backslash mars}}$	0 to 1 *Z	Accounts for male mosquitoes being less likely to go indoors.

The table lists the parameters used in two sensitivity analyses: *Impact of PYR-ITNs on Insecticide Resistance (IR) and Malaria Vector Control* and *Impact of NGM-ITNs on IR and Malaria Vector Control*. The ranges of values were used to assess the sensitivity of model outcomes to variations in input values. The parameters that have static constants are provided in appendix 3. PYR-ITNs: pyrethroid-only ITNs, IR: insecticide resistance, NGM-ITNs: next-generation mixture ITNs, SS: genotype SS, RS: genotype RS, RR: genotype RR, δ_4_ and δ_5_: shape parameters for converting resistance phenotype to blood-feeding phenotype, i_PYR_: indicates the survival possibility to pyrethroid insecticide, i_Novel_: indicates survival possibility to novel insecticide, f^FF^_ITN_: is a blood-feeding probability

Unlike most resistance management models, which include an unspecified mechanism for fitness costs associated with IR [11,12,14,17], the fitness costs in PopGEM are directly attached to specific life-history traits, better representing how fitness costs impact real-world mosquito populations. However, given the expansive parameter space this can add (see section 5.8 of Appendix 5 in S1 File), we did not include fitness costs in the presented simulations. Considering fitness costs are most beneficial when considering the rotation strategy [10] which was not evaluated here, this omission does not impact the model conclusions.

We applied the model in three simulation sets to explore the deployment of three ITN types (agnostic to specific net brands). These ITN types are:

- Pyrethroid-only ITN (PYR-ITN): an ITN containing only a pyrethroid insecticide.
- Next-generation mixture ITN (NGM-ITN): a mixture ITN, containing both a pyrethroid and a single novel insecticide partner in mixture. We assume both insecticides are deployed at full-dose.
- Novel-ITN: a hypothetical ITN, containing only a single novel insecticide, the same that is used in the NGM-ITN. This allows for full quantification of any potential IRM benefit of the NGM-ITN.

PopGEM can account for ITN attrition and insecticide efficacy decay which is explored in Simulation Set 3; but is not included in Simulation Set 1 or 2.

### Simulation Set 1: Impact of PYR-ITNs on IR and Malaria Vector Control

First, we investigated the impact of the evolution of IR considering PYR-ITN only deployments for comparison with previous studies [18,24,25]. This also allowed us to identify parameters driving the spread of IR and the ability to control vector populations via sensitivity analysis.

We compared three scenarios. We first ran a scenario without any interventions (worst case). Next, we ran two scenarios that included interventions with randomly sampled intervention coverage (Table 1), where the coverage was defined as the proportion of households using ITNs. In one simulation, IR was not included and allowed to spread (best case), whereas in the other, IR evolution was allowed. For the IR simulations, the initial genotype frequency was set under Hardy-Weinberg equilibrium (1 = R^2^ + 2RS + S^2^) with inputs S=0·999999, R = 0·000001.

**Fig 2:**
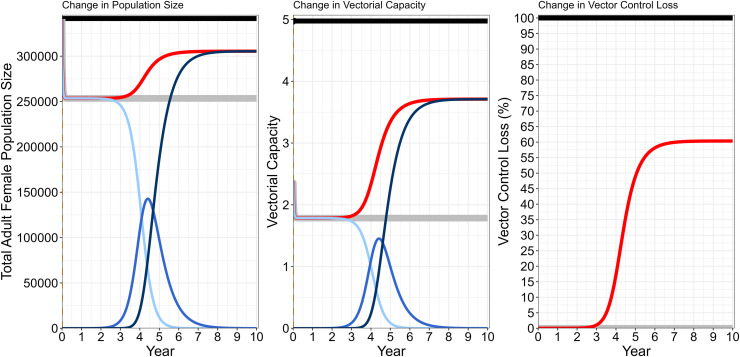
An example of simulation outcomes for the three scenarios of PYR-ITNs: No intervention, intervention without IR, and intervention with IR. For all panels, the black line is the no intervention comparison, the grey line is the intervention without IR, and the red line is the intervention with IR simulation. Within the IR scenarios, genotypes are further stratified by blue shades (SS = light blue, RS = blue, RR = dark blue) for the intervention with IR simulation. Left Panel: Change in the adult female mosquito population size over time across all genotypes. Centre panel: Changes in vectorial capacity over time. Right panel: Changes in vector control loss over time in the simulation where IR can evolve. This is a single simulation to demonstrate the underlying process, parameter values are therefore omitted to avoid overinterpretation of a single simulation run.

From these three scenarios (No Intervention, Intervention with no IR, Intervention with IR), we extracted the vectorial capacity (an entomological measure of transmission, see appendix 6 in S1 File), total adult female population size, and resistance allele frequency at 1-, 3-, 5- and 10-year time horizons. Based on these calculated vectorial capacities, we calculated the vector control loss (%) in the simulation where IR was allowed to evolve. Vector control loss measured the relative reduction in the PYR-ITNs capability to control the mosquito population (i.e., reduction in vectorial capacity) due to the spread of IR (see Equation S6.16 in S1 File). We reported the vector control loss in percentage at 1-, 3-, 5-, and 10-year time horizons. Fig 2 illustrates how changes in resistance allele frequency over time impact adult female mosquito population size and vectorial capacity, causing the progressive loss of vector control in the scenario where IR was allowed to evolve.

For each scenario, we simulated an adult female population size between 10,000 and 1,000,000 depending on larval carrying capacity (see appendix 2 in S1 File). To perform the sensitivity analysis, Latin hypercube sampling [26] was used to generate 100,000 parameter sets (Table 1). Only populations which were viable in the absence of interventions were included in the analysis. Of the 100,000 parameter sets used, 90,637 gave viable populations (see Fig U in S1 File). The sensitivity analysis involved using plots of the parameter density distributions and partial rank correlation coefficients (PRCC) to understand how parameter values impacted vector control loss. The secondary analysis involved understanding the implication of the randomly sampled parameter values on the primary outcomes of malaria vectorial capacity and population size. The analysis was conducted in R, using the package epiR [27] for the PRCC.

### Simulation Set 2: Impact of NGM-ITNs on IR and malaria vector control

NGM-ITNs may demonstrate their full potential (from an IRM perspective) only over long time horizons, which is impractical to measure in field trials. We compared the performance of NGM-ITNs against PYR-ITNs over a long evolutionary timeframe and allowed IR to both insecticides to increase over time dependent on the level of selection.

We assumed that the insecticides on the ITNs were deployed at the full recommended dose. In all simulations, we considered the novel insecticide resistance allele to be rare (frequency of 10^-6^), while pyrethroid resistance allele was moderately frequent (frequency of 0·25), allowing the impact of IRM to be seen for both the novel and pyrethroid insecticides. We assume that the survival phenotype to the NGM-ITN is the product of the survival phenotypes to each individual insecticide.

PYR-ITNs costs have lower per unit costs than NGM-ITNs [28]. This highlights that achieving equivalent coverage with NGM-ITNs would require higher financial investment. To offset costs, there have long been concerns that manufacturers might reduce insecticide doses to reduce per net cost [15]. The alternative may be that donors procure fewer nets, leading to lowering coverage [15]. We accounted for the cost discrepancy by reducing the NGM-ITN coverage by 10%, 20% and 30% of the comparator pyrethroid-ITN coverage, as a reduced coverage may impact both malaria vector control and IRM. This is implemented by scaling the Y parameter (Table 1). We therefore ran six deployment scenarios: PYR-ITN, NGM-ITNs with no reduction, and 10%, 20% and 30% reduction in coverage of the comparator PYR-ITN coverage, and a Novel-ITN. The Novel-ITN (same coverage as PYR-ITN) was used to quantify the full IRM benefit of NGM-ITN.

For each deployment scenario, the same 20,000 parameter sets were used for each deployment type, enabling direct comparisons between the deployment scenarios (Table 1). Only simulations which gave viable populations in the absence of an intervention (defined as the adult female population size being between 10,000 and 1,000,000) were included in the analysis. Therefore, 19,108 (95·54%) of the simulation sets were included in the analysis. Each simulation was run for 15 years. The outcomes were the corresponding resistance allele frequencies to the pyrethroid and novel insecticide, adult female population size, and malaria vectorial capacity. The outcomes were compared across each deployment scenario.

### Simulation Set 3: Impact of NGM-ITNs on IR and Malaria Vector Control with Net Retention and Insecticide Decay

Insecticide decay and ITN retention are two important considerations for both vector control and insecticide resistance evolution. We therefore replicate Simulation Set 2 using the exact same parameter inputs. The one exception is now including decaying insecticide efficacy and ITN retention when ITNs are deployed every three years. In brief, we assume ITNs are retained following a Weibull distribution [29], and similarly that insecticide efficacy also follows a Weibull distribution, with each insecticide having the same decay profile, which is detailed in the Intervention Module in Appendix 1 of the S1 File. Given that in these simulations we are looking at whether the inclusion of decay and retention alters the conclusions from Simulation Set 2, the exact parameterisation is not overly critical. We acknowledge that different ITN brands and different insecticides have different retentions and decays and these are often further location dependent. Rather than using the vectorial capacity at the end of each year (as in Simulation Set 2), we instead compare the yearly sums of the vectorial capacity due to the more complex resistance-transmission dynamics the inclusion of insecticide efficacy decay and ITN retention (Fig 5).

**Fig 3:**
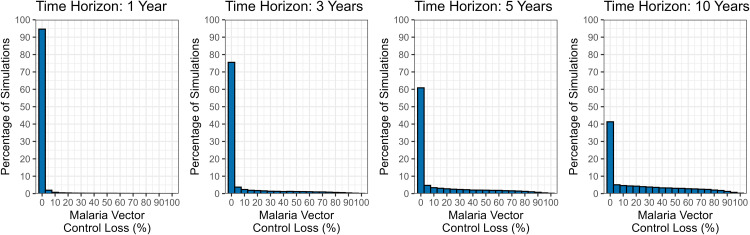
Malaria vector control loss from IR evolution at different time horizons under PYR-ITNs deployments. Each panel reports the distribution of vector control loss due to IR across all simulations (see parameter ranges in Table 1) at the specified time horizon from the 90730 simulations that gave a viable mosquito population (defined as the adult female population size being between 10,000 and 1,000,000 in the absence of interventions). Malaria vector control loss was calculated from equation S6.16 in S1 File). Note, while absolute times are reported, interpretation must consider these as considering short-term (1-3 years) and longer-term (10 years) trends, as precise timescale predictions of evolutionary models are not possible.

**Fig 4:**
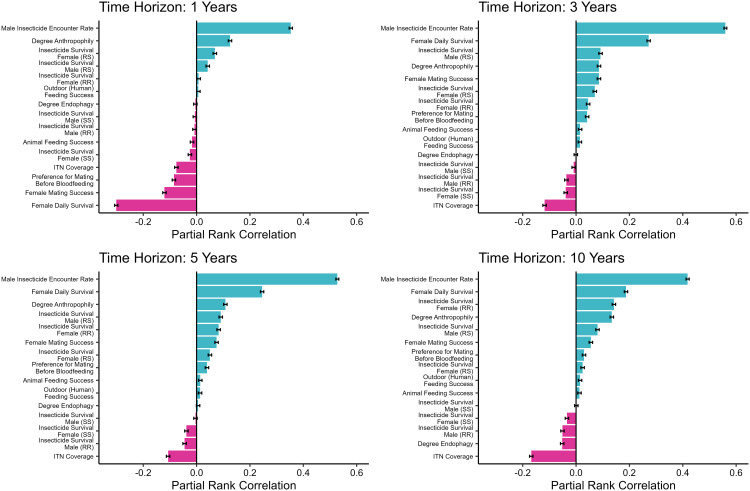
Partial Rank Correlation Coefficient of parameter value on the outcome (vector control loss) at four time horizons. Partial Rank Correlation Coefficients (PRCCs) showing the influence of parameter values (see parameter ranges in Table 1) on vector control loss for a PYR-ITN across four time horizons (1, 3, 5, and 10 years). A positive bar (blue) indicates that increasing the parameter is associated with greater vector control loss, while a negative bar (pink) indicates a reduction in vector control loss. Bar length reflects the magnitude of each parameter’s effect on vector control loss, and error bars represent 95% confidence intervals.

## Results

### Results for Simulation Set 1: Impact of PYR-ITNs on IR and malaria vector control

Fig 3 illustrates how malaria vector control declined over time due to the IR evolution in PYR-ITN scenario across all simulations (see parameter ranges in Table 1). In the first year, the vector control loss was minimal as resistance gene frequencies remained low and most mosquitoes were still susceptible (and effectively controlled). Over time, as the frequency of resistance genes increased, mosquito survival against ITNs increased, resulting in the increased vector control loss. However, as the ITNs still had an impact on homozygote resistant (RR) mosquitoes, even when the resistance gene frequency reached high levels after 10 years, ITNs still contribute to vector control. Therefore, the vector control loss did not reach 100%. In opposition, in some simulations, vectorial capacity remained stable (less than 1% change) even after 10 years indicating the level of selection was not sufficient to cause the resistance allele to be frequent enough to yet compromise vector control in the time horizons evaluated.

To understand why, in some cases, the vector control loss was large, and in some other cases low, we investigated the influence of parameters on vector control loss (Fig 4), but note that due to population viability filtering this means some parameter values occur more frequently than expected in the analysis. At the 10-year mark, the male insecticide encounter rate had the largest impact on vector control loss. Higher rates of male insecticide encounter accelerated IR spread and consequently reduced vector control. Male mosquitoes are often overlooked in this context because they do not transmit *P. falciparum*, however, our simulations demonstrated that their exposure to insecticides significantly influences the spread of resistance genes, accelerating IR development and exacerbating vector control loss.

ITN coverage emerged as the most important factor in preventing vector control loss. While this may seem counterintuitive (since higher ITN coverage accelerated the spread of IR), however once IR had spread, higher ITN coverage led to overall better vector control because higher coverage better suppresses malaria transmission by exposing mosquitoes to more ITNs (that still have some impact). Thus, although insecticide pressure accelerated resistance, maintaining high ITN coverage remained a crucial strategy to sustain effective malaria control. Interestingly, the mosquitoes’ feeding behaviour also had a great impact on vector control loss. Highly anthropophilic species were associated with greater vector control loss, as they were more exposed to ITNs and therefore, any reduction in efficacy (due to resistance) was more noticeable. Similarly, highly endophagic species were associated with higher levels of vector control loss, since they were also more exposed to ITNs.

### Results for Simulation Set 2: Impact of NGM-ITNs on IR and malaria vector control

**Fig 5:**
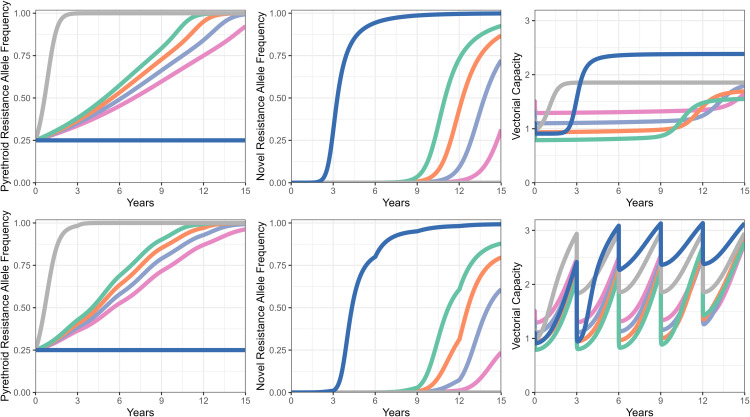
An example simulation plots for the combined IRM and vector control of NGM-ITNs for Simulation Sets 2 and 3. An example simulation is shown to give a representation of how the simulation performs for the three outcomes of pyrethroid resistance allele frequency (left panel), novel resistance allele frequency (centre panel), and vectorial capacity (right panel). This simulation was selected randomly, and we do not report here the randomly sampled parameter values used in the simulation to prevent over-interpretation. Within this simulation run, six simulations were conducted, one for each of the interventions. In all plots, the grey line is the PYR-ITN only run. The green line is NGM-ITN without coverage reduction (relative to the standard pyrethroid-only ITN coverage). The orange line (10% coverage reduction), purple line (20% coverage reduction), and pink line (30% coverage reduction) are the NGM-ITN simulations where coverage was reduced when compared to the standard PYR-ITN coverage. The blue line is the Novel-ITN, and was at the same coverage as to the standard PYR-ITN coverage. The simulations are replicated with the same input parameter values except without (top row) and with (bottom row) insecticide efficacy decay and ITN retention.

We next examined the effects of NGM-ITNs on IRM and malaria vector control. Fig 5 presents a randomly selected simulation that illustrate the typical impact of the different scenarios on the spread of IR. Generally, deploying NGM-ITNs slowed the spread of pyrethroid resistance compared to deploying PYR-ITNs. Moreover, lower coverage of NGM-ITNs further reduced the spread of pyrethroid resistance. A similar pattern was seen for resistance to the novel insecticide, where deploying NGM-ITNs provided greater IRM benefits than deploying Novel-ITNs (since the pyrethroid still had a minor effect), and reducing the coverage of the NGM-ITNs increased this IRM benefit. Further sensitivity analysis is presented in appendix 7 in S1 File.

**Fig 6:**
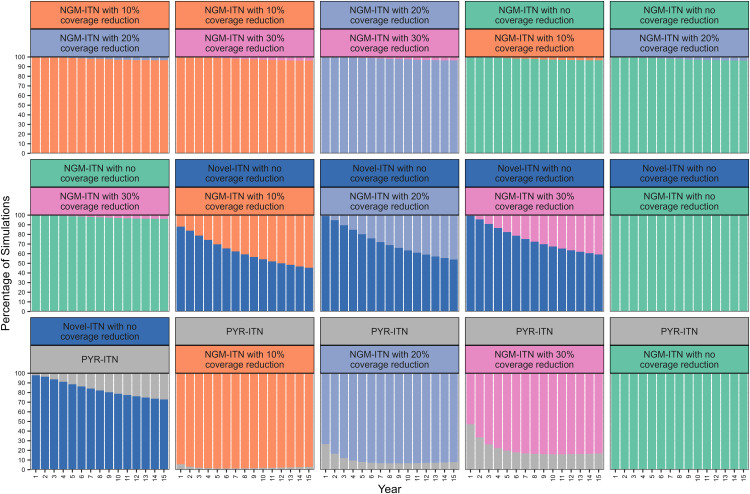
Comparative performance of the different ITN deployments over time. The figure shows the percentage of simulations (see parameter range in Table 1) that had a lower vectorial capacity between the comparator strategies (PYR-ITN, NGM-ITN, and Novel-ITN) at yearly time points. A lower vectorial capacity is defined as being more effective, as there is less transmission. The panel descriptors describe which two deployment options are being compared. The colour scheme is the same as for Fig 5, where the bar colours are used to designate the winning strategy (lower vectorial capacity). For example, for the top left panel, this would be read as comparing NGM-ITN with 10% coverage reduction and NGM-ITN at 20% coverage reduction, where the orange bars indicate the percentage of simulations where the NGM-ITN with 10% coverage reduction winning (having a lower vectorial capacity), and the purple bars indicates this for the NGM-ITN at 20% coverage reduction won.

We compared the differences in vectorial capacity of the different types of ITNs at yearly intervals across all simulations (see parameter ranges in Table 1), where the strategy with the lowest vectorial capacity was declared winning (Fig 6). At equal coverage levels, NGM-ITNs consistently reduced vectorial capacity more than PYR-ITNs. Even when deployed at reduced coverage, NGM-ITNs generally outperformed PYR-ITNs. The benefit of NGM-ITNs at reduced coverage becomes more apparent over time as rising pyrethroid resistance reduces the effectiveness of PYR-ITNs. When comparing the different reduced coverage levels of NGM-ITNs, the higher coverage almost always provided stronger vector control than the lower coverages. However, in a small subset of simulations, lower coverages appeared in some simulations to provide better vector control than the higher coverage in long term. We suspected that lower coverage exerted less selection pressure for resistance, resulting in the slower development of IR, which allowed NGM-ITNs to maintain their effectiveness over a longer time-period, but this is offset by a reduced ability to control mosquito populations early in the simulations.

An operational question facing manufacturers is whether there is an advantage of mixing their novel chemistries with pyrethroids. Providing coverage is equal, the NGM-ITNs consistently outperform a Novel-ITN, as the pyrethroid can still have a minor mosquito killing effect. However, when NGM-ITN coverage is reduced, the Novel-ITN can becomes preferable in early years, but becomes less beneficial over time as resistance to the novel insecticide evolves more rapidly than when in a NGM-ITN.

**Fig 7:**
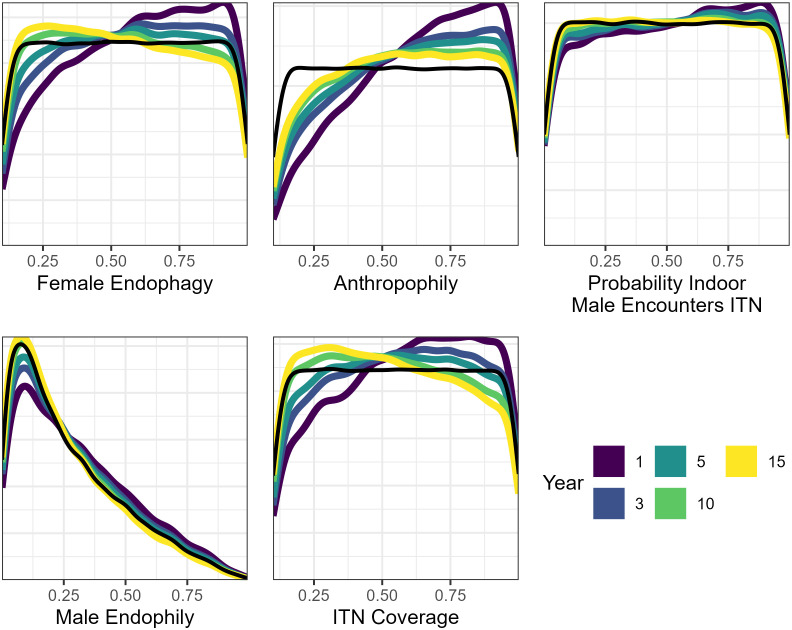
Parameter Distributions for Simulations where the NGM-ITN at 30% coverage reduction outperforms the PYR-ITN in reducing vectorial capacity. This compares the parameter distributions for all simulations (black line). The coloured lines show simulations at each different time horizons at where the NGM-ITN with 30% coverage reduction outperformed the PYR-ITN. The NGM-ITN with 30% coverage reduction was chosen as the comparator because it highlights a moderate reduction in coverage. Where the coloured lines are above the black line this means the parameter values are over-represented (portions of the parameter space where NGM-ITNs with 30% coverage reduction outperform PYR-ITNs), and where the coloured lines are below the black line this means the parameter values are under-represented (portions of the parameter space where PYR-ITNs outperform NGM-ITNs with 30% coverage reduction).

We investigated in which conditions where NGM-ITNs with a 30% coverage reduction were more effective than PYR-ITNs (Fig 7). This happened in simulations where pyrethroid was still (mostly) effective at killing female resistant mosquitoes. However, over time, as the RR genotype became more prevalent in the population, the benefit of switching to NGM-ITN (despite being at lower coverages) increased. This supports the recommendation that NGM-ITNs should be used in areas of high pyrethroid resistance. PYR-ITNs were also better if females were not very endophagic or anthropophilic. If female mosquitoes are not very endophagic or anthropophilic, there is a need for more nets rather than fewer effective better nets. This is because mosquitoes would be unlikely to encounter an NGM-ITN at the reduced coverage, and therefore they would not be able to provide the improved protection to individuals or the wider community (through increased mosquito mortality and population suppression). Further sensitivity analysis is presented in appendix 7.

### Results for simulation Set 3: Impact of NGM-ITNs on IR and malaria vector control with net retention and insecticide decay

**Fig 8:**
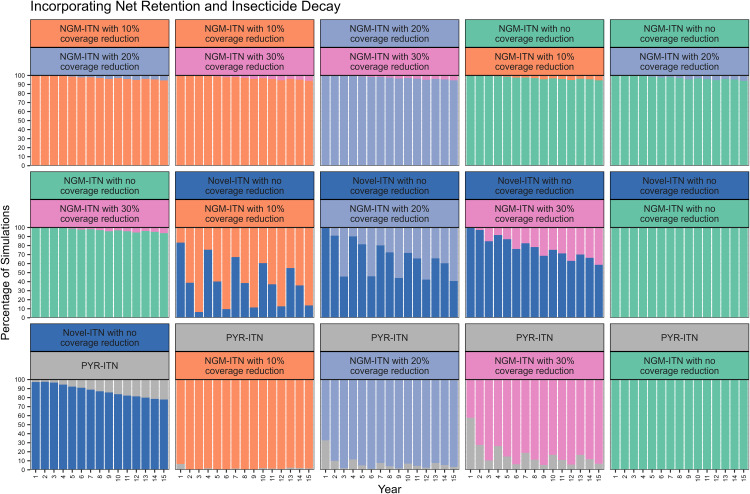
Comparative performance of the different ITN deployments over time accounting for ITN retention and insecticide decay. The figure shows the percentage of simulations (see parameter range in Table 1) that had a lower vectorial capacity between the comparator strategies (PYR-ITN, NGM-ITN, and Novel-ITN) at yearly time points. A lower vectorial capacity is defined as being more effective, as there is less transmission. The panel descriptors describe which two deployment options are being compared. The colour scheme is the same as for Fig 5, where the bar colours are used to designate the winning strategy (lower vectorial capacity). For example, for the top left panel, this would be read as comparing NGM-ITN with 10% coverage reduction and NGM-ITN at 20% coverage reduction, where the orange bars indicate the percentage of simulations where the NGM-ITN with 10% coverage reduction winning (having a lower vectorial capacity), and the purple bars indicates this for the NGM-ITN at 20% coverage reduction won.

Fig 8 shows the comparison accounting for ITN retention and the decay of insecticide efficacy between re-deployments. Overall, the same qualitative conclusions can be drawn as for Simulation Set 2. The example simulation in Fig 5 highlights how the inclusion of ITN retention and insecticide efficacy decay may marginally improve the IRM aspect as coverage lowers. Albeit decay in insecticide efficacy increases the survival probabilities of heterozygous and homozygous resistant individuals.

## Discussion

How IR evolution impacts vector-borne disease transmission is a key question in public health [3]. We developed PopGEM, a model that enables the integration of mosquito population dynamics and resistance genetics to explore the long-term impact of different types of ITN on malaria transmission, considering their impact on the evolution of IR. Our PYR-ITN simulations indicated (as with previous simulation models [18,25,30]) that as IR increases in the population, the impact of PYR-ITNs on malaria vector control decreases. However, our use of a wide parameter space gives additional insight into the parameters driving both IR evolution and malaria vector control. Furthermore, our work extends prior models by evaluating NGM-ITNs for both their IRM benefits and efficacy as vector control tools.

### The dual benefit of NGM-ITNs for vector control and IRM

In our simulations, NGM-ITNs were more effective than PYR-ITNs not only because it is a superior intervention with a greater mosquito killing effect, but also because NGM-ITNs provide an IRM benefit that extends their impact over a longer timeframe. Trials evaluating NGM-ITNs may underestimate their value by not fully accounting for the additional long-term IRM benefits compared to PYR-ITNs. This is our key operational finding, which supports the WHO recommendation of using mixture ITNs [31].

### The role of coverage and economic trade-offs in ITN deployment

Increasing ITN coverage is beneficial for controlling malaria transmission, however, increasing coverage inevitably comes at the expense of increased selection for IR. Nevertheless, our simulations highlight that maintaining high coverage of ITNs can also help mitigate the negative impact of IR on malaria vector control. ITNs reduce transmission in two ways. First, by providing a personal protective chemical and physical barrier reducing an individual’s likelihood of being bitten. Second, by providing community protection through mass killing, which reduces mosquito survival rates and population sizes. ITN users benefit from both personal protection and community protection, while non-users only benefit from community protection. As IR in the mosquito population increases there is a declining efficacy of ITNs. This, in turn, can lead to an increase in mosquito population size and mosquito survival, which reduces the overall community-level protective effect. To offset this effect, an increase in ITN coverage is needed. Killeen [32] argued that the mass effect of ITNs as a result of increased mortality rates in mosquitoes was of such critical importance that a decrease in coverage is justifiable if more lethal ITNs are distributed. Our simulations considering the deployment of NGM-ITNs were consistent with this argument.

One issue which NGM-ITNs have long since had is that both insecticides in NGM-ITNs must be deployed at their respective full-doses for their IRM efficacy to be highest [11,17,33]. There have long been concerns that to compensate for the increased costs of NGM-ITNs, the dose of each respective insecticide partner may be reduced [15]. However, reduced dose NGM-ITNs are less efficacious for IRM than a full dose NGM-ITNs [17,33]. Consequently, we expect reduced coverage (in the short term) to be an inevitability due to the limited budgets of national malaria control programmes and higher cost of NGM-ITNs. From a combined IRM and disease management perspective, if the increased cost of the mixtures must be offset, reducing coverage is the option to choose as this does not compromise IRM in the way that reducing dosing does [33] (and should further encourage manufacturers to develop full-dose mixtures). Due to the superior performance of NGM-ITNs versus PYR-ITNs in reducing transmission [7,8], this reduction in coverage may not compromise malaria control, while extending the “resistance lifespan” of the mixture, as reducing coverage inevitably reduces selection. Rather than compromising on the dosing of the NGM-ITNs (where the maximal IRM efficacy is obtained), the economic compromise, which may be better for both epidemiological outcomes and IRM, is to reduce the coverage of NGM-ITNs, and the magnitude of this reduction will be dependent on the transmission setting. These findings were further found to be robust when including insecticide efficacy decay and ITN retention.

### Role of male mosquitoes

Although male mosquitoes are widely regarded as unimportant for malaria transmission, we identified their importance in accelerating the loss of malaria vector control due to increasing IR. Male mosquitoes are critical in the spread of resistance genes, as they provide half the genes for the next generation. Our knowledge of male mosquitoes is often severely limited [34], and there is little understanding of the insecticide selection pressures on adult male mosquitoes in the field. Models which have evaluated exclusively the impact of IRM strategies have shown that while selection on male mosquitoes accelerates the spread of IR [10], this is often considered a low issue (because models scale selection so males do not encounter insecticides as frequently as females). However, by reframing the evaluation of the impact of insecticide selection by interventions on vectorial capacity, we instead see that male mosquitoes increase in their importance. We can frame this by understanding that IR selection on male mosquitoes is inherently wasted, because male mosquitoes play no direct role in transmission, however, when considering resistance, they help propagate IR through the population. Further studies should better evaluate the exposure of male mosquitoes to insecticides and monitor the level of IR in male mosquitoes to address this knowledge gap.

### Model limitations

As with any modelling study, there are some limitations which must be addressed. Each simulation had an upper limit on IR, and this may not be applicable to areas with intense levels of IR [35]. The three gene model version (see appendix 5 of the S1 File) would allow for the cumulative addition of resistance genes, which may be more suitable for dealing with higher resistance intensities. Seasonality and environmental conditions also impact mosquito population dynamics, however, to focus directly on the impact of resistance evolution we have not considered this. The potential for behavioural resistance to evolve was not considered, however behavioural resistance (whereby mosquitoes adapt to avoid interventions) would impact both insecticide selection pressure and transmission, and so is likely to have complicated interwoven dynamics. Finally, we used vectorial capacity as our measure of transmission. The advantage of this is the generalizability to multiple mosquito-borne disease systems (malaria, dengue, lymphatic filariasis), where resistance is also a key consideration. This comes with the benefit of not needing to additionally extend the model to directly include malaria transmission and the human malaria lifecycle. However, we expect IR to have interactions with transmission intensity and the immunity of the population to malaria (as seen with malaria drug resistance [36]). By reporting vectorial capacity, we have negated these complications to better aid model results communication, but understand this may lead to underestimation of epidemiological benefits of IRM.

## Conclusion

The inclusion of IR genetics into models of mosquito population dynamics is a complex challenge. However, a consistent narrative is emerging across the various modelling strands: NGM-ITNs are the best available IRM tool, whose interaction with transmission control is beneficial, and that NGM-ITNs are superior to PYR-ITNs. Given this growing body of evidence, we suggest NMCPs should prioritise the transition to NGM-ITNs to optimise both malaria transmission control and IRM. Continued reliance on PYR-ITNs only increases the risk of IR and failure of malaria vector control. This approach is increasingly outdated and counterproductive. Considering the findings presented in this study, the adoption of NGM-ITNs should be strongly promoted from both a malaria vector control and IRM standpoint. Furthermore, male mosquitoes play an important role in the selection of insecticide resistance, and understanding their exposure to insecticides should be given more consideration.

## Data sharing

This study did not utilize individual-level participant data. Model parameters were informed by values reported in existing literature, as detailed in the main text and appendix 2 of the S1 File. All datasets and scripts used to generate the figures are accessible at https://osf.io/ehbj2/overview. The simulation and analysis code is also available at https://github.com/NeilHobbs/PopGEM.

## Supporting information

S1 File Mathematical model equations and additional sensitivity analysis. (PDF)
