# Supplementary material for: Impact of insecticide resistance evolution on malaria vector control
Source: PLoS Comput Biol. 2026 Aug 3;22(8):e1014612. doi: 10.1371/journal.pcbi.1014612 (PMC13446747; doi:10.1371/journal.pcbi.1014612)
Supplement: Supplementary file 1 [file pcbi.1014612.s001.pdf]

Mathematical model equations and additional sensitivity analysis

Table of Contents

Table of Contents 1
Appendix 1: PopGEM model description 2
1.1 Oviposition Module 4
1.2 Aquatic Module 6
1.2.1. Aquatic Module: Larvae 7
1.2.2. Aquatic Model: Density Dependent Population Regulation 9
1.3 Adult Male Module 11
1.4 Adult Unmated Female Module 13
1.5 Mating Module 16
1.6 Mated Female Module 18
1.7 Blood-feeding Module 19
1.8 Intervention Module 20
Appendix 2: State Variables and Parameters in PopGEM and Their Descriptions 20
Appendix 3: Parameter Estimation 26
Appendix 4: Climate Module 31
4.1 Impact of Climatic Conditions: 31
4.2 Carrying Capacity: 31
4.3 Impact of Temperature on Mosquito Biological Parameters 31
Appendix 5: Extension to a two and three gene system: 37
5.1 Update to the Oviposition Module 37
5.2 Update to the Aquatic Module 39
5.3 Update to the Adult Male Module 42
5.4 Update to the Unmated Female Model 44
5.5 Update to the Mating Model 45
5.6 Update to the Mated Female Module 46
5.7 Defining insecticide survival in the two and three gene model 47
Defining fitness costs associated with resistance in the two and three gene model 49
Appendix 6: Vectorial Capacity and Vector Control Loss (%) 53
Appendix 7: Additional Sensitivity Analysis 56
References 62

Appendix 1: PopGEM model description

PopGEM (Population Genetics and Ecology in Mosquitoes) is a stage structured model, incorporating both the aquatic (eggs, larvae, pupae) and adult stages of mosquitoes, including both male and female mosquitoes (Fig A which is a more detailed version of Fig 1 in the main manuscript). Male mosquitoes are required as part of the population genetics relevant to insecticide resistance (IR) mating portion of the model, alongside their role in density dependence at the larval stages. IR is included in the model as a classically monogenic trait, that are resistance genes are encoded at single loci, with two alleles per loci (Susceptible = S, Resistance = R). The model is divided into distinct but linked modules, which allows for subsequent future extensions and modifications. PopGEM runs in discrete daily time steps, as is common with other models which have modelled mosquito population dynamics [1,2]. In this Appendix (Appendix 1), the methodology for a single gene (with a table of parameter in appendix 2; and parameter estimation in Appendix 3). We describe how this can be further extended to include climate (Appendix 4) and multiple genes (Appendix 5).

The model code is written in R, and is available from the GitHub repository: https://github.com/NeilHobbs/PopGEM, which includes model code and code for running and analysing the simulations. The PopGEM R code was written in a modular format, allowing for unit testing[3] to ensure the model performs as expected.

To aid the reading of the equations, we use:

- The symbols ♀ and ♂ to refer to female and male mosquitoes respectively
- For female genotypes we use the letters FF (which can be either the R or S genes), and male genotypes we use the letters MM (which can be either the R or S genes). When referring to mated female mosquitoes we denote them FF_MM, to highlight both their own genotype and that of the male mosquito with which she mated.
- A full list of all parameters and plausible parameter values are given in the tables in Appendix 2.
- We introduce the single gene model version (as described here in Appendix 1), before layering over the increased genetic complexity with the two/three gene version as described in Appendix 5.

The model equations are extensive, because the model must simultaneously consider both population dynamic and resistance dynamic processes. Due to the current uncertainty as to which parameters are needed to effectively simulate these processes together, this means some parameters and features may be included even if not explored. Furthermore, as the simulation of both these processes in a single framework is still at the early stages, there remains substantial unknowns regarding how these processes interact (see the results for male mosquitoes in the main manuscript as an obvious example). The equations also include the ability to simulate features which we have not conducted in this investigation (e.g., life-history associated fitness costs which can be sex and life-stage specific). This is a key additional benefit of the model, and allows for subsequent investigations to further evaluate insecticide resistance management strategies in the context of malaria vector control.

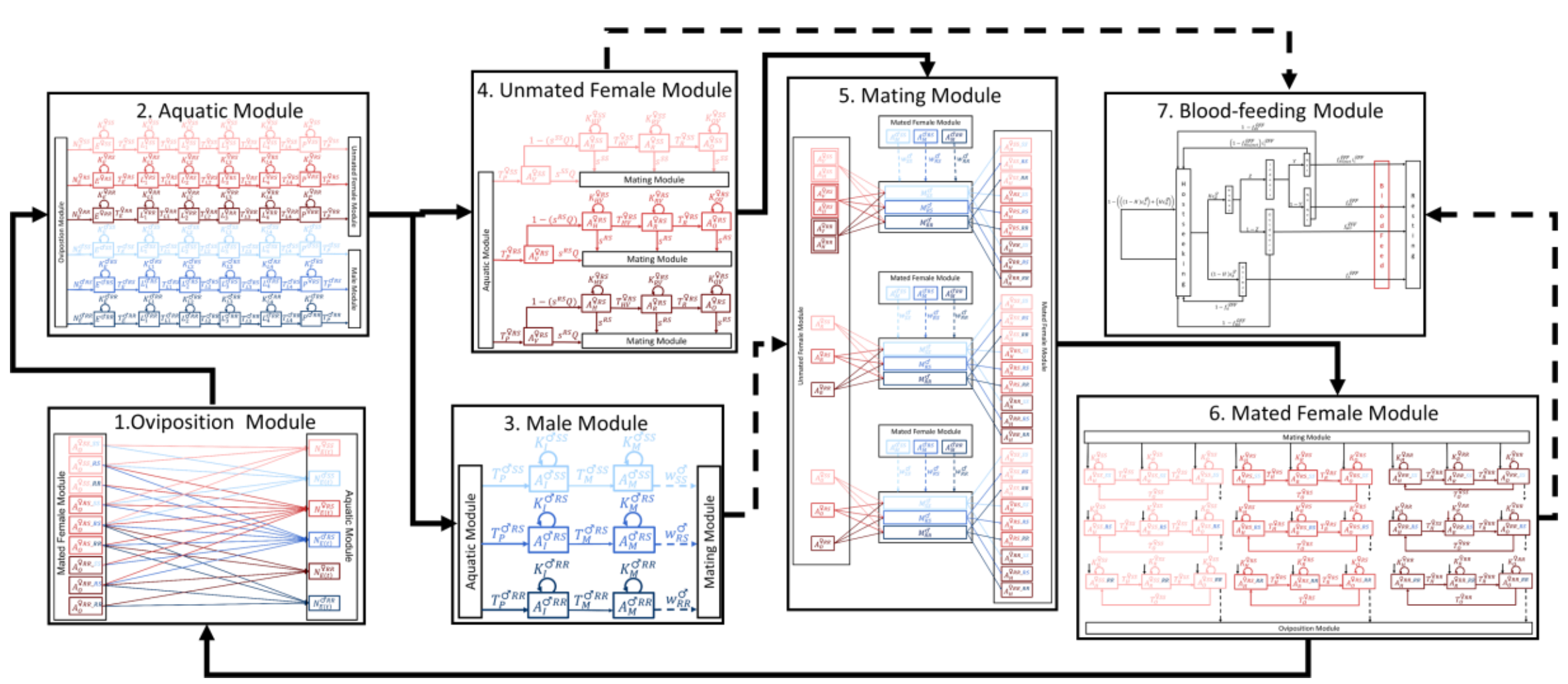

Fig A: Overview of the PopGEM (Population Genetics and Ecology of Mosquitoes) model

PopGEM consists of seven linked modules, where each module is fully described in Appendix 1. Eggs are laid by mated female mosquitoes in the Oviposition Module (1), which then enter the Aquatic Module (2), to develop through the larval instars and into pupae. Adult mosquitoes emerge, where males enter the Male Module (3). Newly emerged female mosquitoes enter the Unmated Female Module (4). Mating between adult male and female mosquitoes is implemented in the Mating Module (5). Females which successfully mate enter the Mated Female Module (6). Adult female mosquitoes can blood-feed in the Blood-feeding Module (7), where they can interact with insecticide treated nets. It is in the Blood-feeding Module (7), where differential survival to insecticides deployed as insecticide-treated nets is implemented where survival depends on the mosquito genotype. Colours are used to differentiate males (blues) and females (reds), and the shades are used to indicate the genotype where the lightest shade is the homozygous susceptible (SS), the darkest shade is the homozygous resistant (RR) and the intermediate shade is the heterozygote (RS).

1.1 Oviposition Module

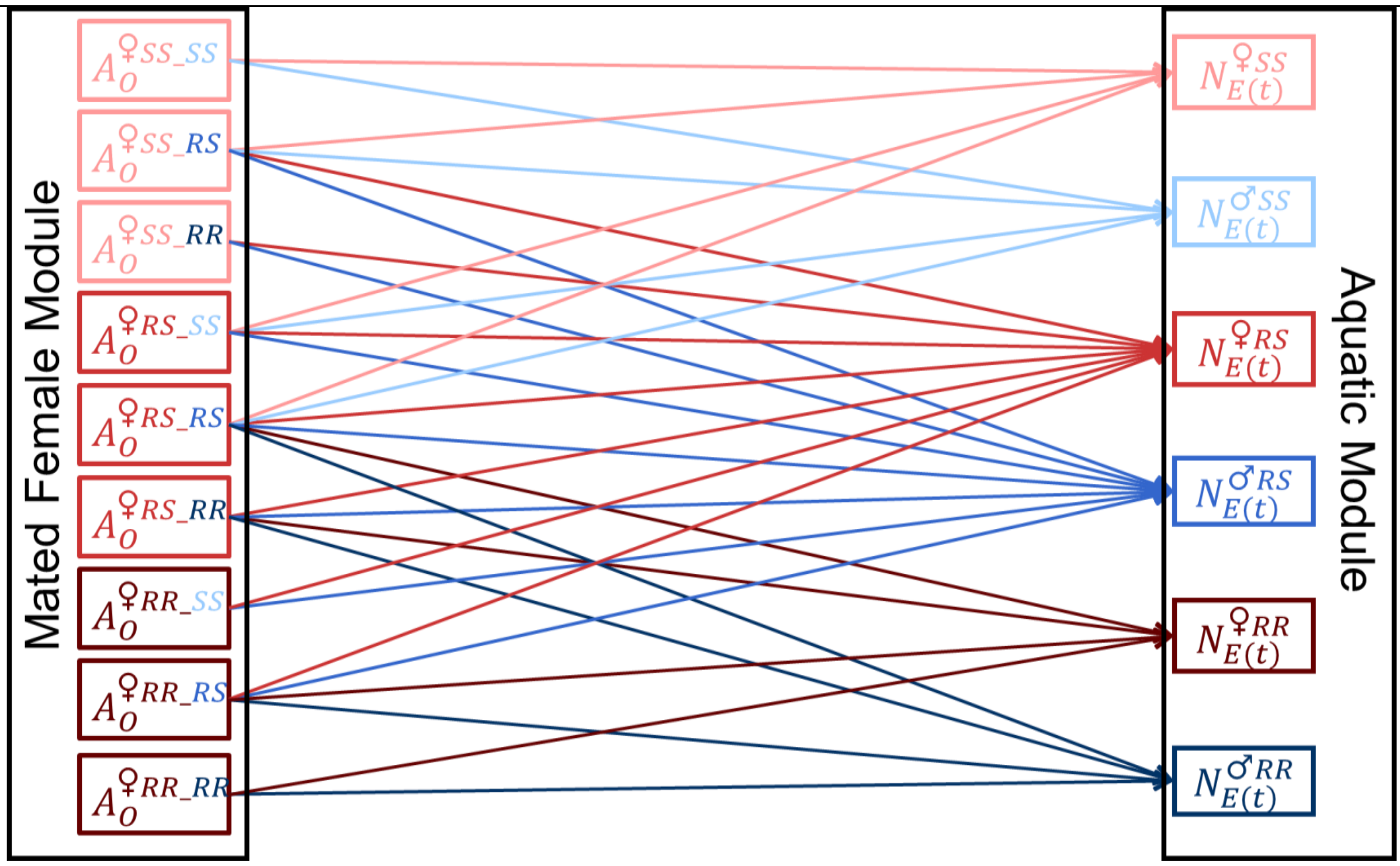

Fig B: Oviposition Module

Adult female mosquitoes that have mated and entered the oviposition stage of their gonotrophic cycle can lay eggs. The genotype of the eggs laid depends on both the female's genotype and that of her male mating partner. Therefore, female mosquitoes with different mated genotype permutations produce eggs of varying genotypes (indicated by arrow colour), and in different proportions, as calculated in equations S1.1.1 to S1.1.2. These eggs then enter the aquatic module. ♀, ♂: Denote female and male sex, respectively. S, R: Represent susceptible (S) and resistant (R) alleles. Genotypes (e.g., SS, SR, RR) refer to allele permutations. $A_O^{♀FF\_MM}$: Number of female mosquitoes at oviposition, where FF is the female genotype and MM is the genotype of her mating male (example: $A_O^{♀SS\_RS}$ indicates the number of homozygous susceptible females mated with heterozygous resistant males at oviposition). $N_{E(t)}^{XGG}$: Number of eggs laid at time t, with genotype GG and sex X (example: $N_{E(t)}^{♀RR}$), is the number of eggs laid at time t that are homozygous resistant females).

In the Oviposition Module (Fig B) egg laying occurs. Eggs are laid by mated female mosquitoes during the oviposition stage of their gonotrophic cycle. The oviposition module links the Mated Female Module and the Aquatic Module. The number of eggs of each sex and genotype being laid each day are calculated. Mated female mosquitoes have the mated genotype denoted in the superscript FF_MM, where FF is the females own genotype, and MM is the genotype of the male she mated. For a single gene set-up this gives nine possible mated genotypes permutations.

The number of eggs (female and male) laid by female mosquitoes with each mated genotype will be:

$$F_{E(t)}^{♀FF\_MM} = A_{O(t)}^{♀FF\_MM} \theta_{(t)}^{FF} \, \Phi^{FF} a_o^{♀FF}$$

Equation S1.1.1(♀)

$$F_{E(t)}^{♂FF\_MM} = A_{O(t)}^{♀FF\_MM} \theta_{(t)}^{FF} \, (1 - \Phi^{FF}) a_o^{♀FF}$$

Equation S1.1.1(♂)

$F_{E(t)}^{♀FF\_MM}$ and $F_{E(t)}^{♂FF\_MM}$ are the total number of female and male eggs laid by adult females in the oviposition state with the mated genotype FF_MM ($A_{O(t)}^{♀FF\_MM}$) on day t. $\theta_{(t)}^{FF}$ is the number of eggs laid per female with the genotype

FF. Φ^{FF} is the proportion of female eggs (out of all eggs) laid by females with the genotype FF. $a_o^{♀FF}$ is proportion of females in the oviposition stage with the genotype FF who successfully oviposit.
$F_{E(t)}^{♀FF\_MM}$ and $F_{E(t)}^{♂FF\_MM}$ are then distributed through classical Mendelian inheritance. The total number of female and male eggs of each genotype being laid on day t is:

$$N_{E(t)}^{♀SS} = F_{E(t)}^{♀SS\_SS} + \left(\frac{F_{E(t)}^{♀SS\_RS} + F_{E(t)}^{♀RS\_SS}}{2}\right) + \left(\frac{F_{E(t)}^{♀RS\_RS}}{4}\right)$$

Equation S1.1.2(a♀)

$$N_{E(t)}^{♂SS} = F_{E(t)}^{♂SS\_SS} + \left(\frac{F_{E(t)}^{♂SS\_RS} + F_{E(t)}^{♂RS\_SS}}{2}\right) + \left(\frac{F_{E(t)}^{♂RS\_RS}}{4}\right)$$

Equation S1.1.2(a♂)

$$N_{E(t)}^{♀RS} = F_{E(t)}^{♀SS\_RR} + F_{E(t)}^{♀RR\_SS} + \left(\frac{\left(F_{E(t)}^{♀SS\_RS} + F_{E(t)}^{♀RS\_SS} + F_{E(t)}^{♀RS\_RS} + F_{E(t)}^{♀RS\_RR} + F_{E(t)}^{♀RR\_RS}\right)}{2}\right)$$

Equation S1.1.2(b♀)

$$N_{E(t)}^{♂RS} = F_{E(t)}^{♂SS\_RR} + F_{E(t)}^{♂RR\_SS} + \left(\frac{\left(F_{E(t)}^{♂SS\_RS} + F_{E(t)}^{♂RS\_SS} + F_{E(t)}^{♂RS\_RS} + F_{E(t)}^{♂RS\_RR} + F_{E(t)}^{♂RR\_RS}\right)}{2}\right)$$

Equation S1.1.2(b♂)

$$N_{E(t)}^{♀RR} = F_{E(t)}^{♀RR\_RR} + \left(\frac{\left(F_{E(t)}^{♀RS\_RR} + F_{E(t)}^{♀RR\_RS}\right)}{2}\right) + \left(\frac{F_{E(t)}^{♀RS\_RS}}{4}\right)$$

Equation S1.1.2(c♀)

$$N_{E(t)}^{♂RR} = F_{E(t)}^{♂RR\_RR} + \left(\frac{\left(F_{E(t)}^{♂RS\_RR} + F_{E(t)}^{♂RR\_RS}\right)}{2}\right) + \left(\frac{F_{E(t)}^{♂RS\_RS}}{4}\right)$$

Equation S1.1.2(c♂)

1.2 Aquatic Module

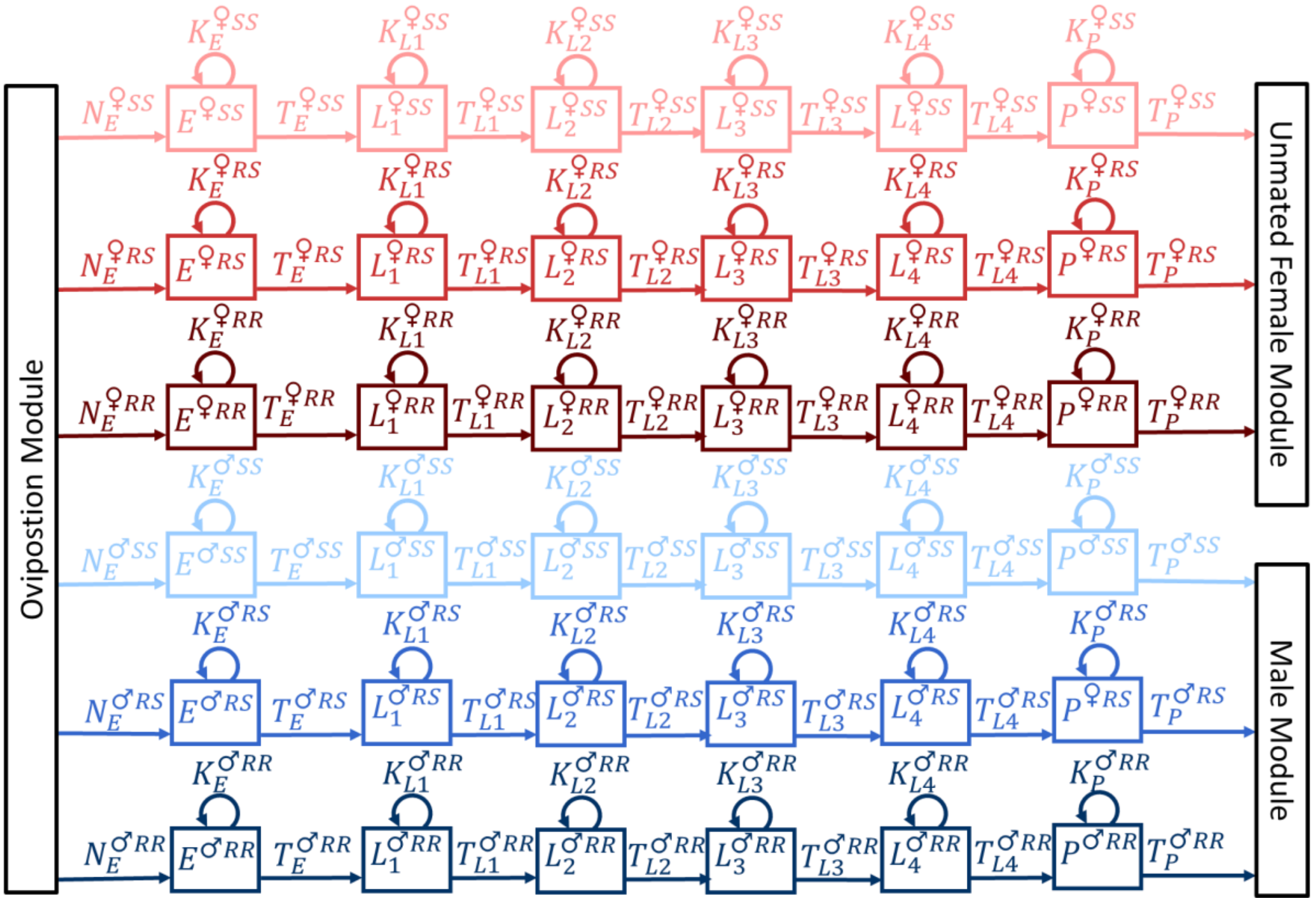

Fig C: Aquatic Module

A number of eggs are laid (N_E) by ovipositing females (as detailed in the Oviposition Module, equations S1.1.2). Eggs (E) hatch and transition to first-instar larvae stage (L1), then to the second (L2), third (L3), and fourth (L4) instar larvae. Survival of the larval instars is density-dependent (see equations S1.2.8 to 1.2.9). Fourth-instar larvae (L4) then transition into pupae (P), which undergo metamorphosis prior to emergence. Emerging adult mosquitoes transition to the next module according to their sex: females enter the Unmated Female module (see section 1.4), while males enter the Adult Male module (see section 1.3). The different genotypes and sex permutations are highlighted in different colours. ♀, ♂: Denote female and male sex, respectively. S, R: Represent susceptible and resistant alleles. Genotypes (e.g., SS, SR, RR) refer to allele permutations. Y^{XGG}: Number of mosquitoes at stage Y, with genotype GG and sex X (example: $L_1^{♀RR}$ is the number of female L1 larvae with the homozygous resistant genotypes). T_Y^{XGG}: the proportion of mosquitoes from stage Y with genotype GG and sex X transitioning to the next stage (example: $T_{L1}^{♀RR}$ is the proportion of female larvae with homozygote resistant genotype transitioning from L1 to L2 larval instars). K_Y^{XGG}: the proportion of mosquitoes from stage Y with genotype GG and sex X that do not transition to the next stage but survive (example: $K_{L1}^{♀RR}$ is the proportion of female larvae with homozygote resistant genotype not transitioning from L1 to L2 instars, but surviving to remain as L1 larval instars).

The aquatic module concerns the development, growth and survival of eggs, larvae and pupae. A number of eggs are laid by ovipositing females (as calculated in the oviposition module, equations S1.1.2) and can then hatch and transition to the larvae stages. Larvae are categorised into four instars (stages) (L1, L2, L3, and L4). The number of eggs per genotype and sex at time t depends on the number of newly laid eggs and the number of surviving unhatched eggs:

$$E_{(t)}^{♀FF} = N_{E(t)}^{♀FF} + \left(K_{E(t-1)}^{♀FF} E_{(t-1)}^{♀FF}\right)$$

Equation S1.2.1(a♀)

$$E_{(t)}^{♂MM} = N_{E(t)}^{♂MM} + \left(K_{E(t-1)}^{♂MM} E_{(t-1)}^{♂MM}\right)$$

Equation S1.2.1(a♂)

$E_{(t-1)}^{♀FF}$ and $E_{(t-1)}^{♂MM}$ are the number of eggs from the previous day. $K_E^{♀FF}$ and $K_E^{♂MM}$ are the proportion of eggs which survived did not transition to L1 the previous day:

$$K_{E(t)}^{♀FF} = \rho_{E(t)}^{♀FF}\left(1 - h_{E(t)}^{♀FF}\right)$$

Equation S1.2.2(b♀)

$$K_{E)t)}^{♂MM} = \rho_{E(t)}^{♂MM}\left(1 - h_{E(t)}^{♂MM}\right)$$

Equation S1.2.2(b♂)

This depends on the survival probability of eggs ($\rho_{E(t)}^{♀FF}$ and $\rho_{E(t)}^{♂MM}$) and egg hatch probability ($h_{E(t)}^{♀FF}$ and $h_{E(t)}^{♂MM}$).

1.2.1. Aquatic Module: Larvae

The number of L1 instars for each genotype and sex at time t is:

$$L1_{(t)}^{♀FF} = L1_{(t-1)}^{♀FF}K_{L1(t-1)}^{♀FF} + E_{(t-1)}^{♀FF}T_{E(t-1)}^{♀FF}$$

Equation S1.2.3(a♀)

$$L1_{(t)}^{♂MM} = L1_{(t-1)}^{♂MM}K_{L1(t-1)}^{♂MM} + E_{(t-1)}^{♂MM}T_{E(t-1)}^{♂MM}$$

Equation S1.2.3(a♂)

$K_{L1}^{♀FF}$ and $K_{L1}^{♂MM}$ are the proportion of the L1 instars which did not grow sufficiently to transition to L2, while still surviving. $T_E^{♀FF}$ and $T_E^{♂MM}$ are the proportion of eggs hatching to give L1 larvae.

$$K_{L1(t)}^{♀FF} = \rho_{L1(t)}^{♀FF}\left(1 - \delta_{L1(t)}^{♀FF}\right)$$

Equation S1.2.3(b♀)

$$K_{L1(t)}^{♂MM} = \rho_{L1(t)}^{♂MM}\left(1 - \delta_{L1(t)}^{♂MM}\right)$$

Equation S1.2.3(b♂)

$$T_{E(t)}^{♀FF} = \rho_{E(t)}^{♀FF}h_{E(t)}^{♀FF}$$

Equation S1.2.3(c♀)

$$T_{E(t)}^{♂MM} = \rho_{E(t)}^{♂MM}h_{E(t)}^{♂MM}$$

Equation S1.2.3(c♂)

$\rho_{L1}^{♀FF}$ and $\rho_{L1}^{♂MM}$ are the daily survival probabilities, and are updated each day to incorporate density-dependence (see Section 1,2.2, equations S1.2.8 to S1.2.9). $\delta_{L1(t)}^{♀FF}$ and $\delta_{L1(t)}^{♂MM}$ are probabilities that L1 larvae transition to L2 larvae.

This process is similar for L2, L3 and L4 instars:

$$L2_{(t)}^{♀FF} = L2_{(t-1)}^{♀FF}K_{L2(t-1)}^{♀FF} + L1_{(t-1)}^{♀FF}T_{L1(t-1)}^{♀FF}$$

Equation S1.2.4(a♀)

$$L2_{(t)}^{♂MM} = L2_{(t-1)}^{♂MM}K_{L2(t-1)}^{♂MM} + L1_{(t-1)}^{♂MM}T_{L1(t-1)}^{♂MM}$$

Equation S1.2.4(a♂)

$$K_{L2(t)}^{♀FF} = \rho_{L2(t)}^{♀FF}\left(1 - \delta_{L2(t)}^{♀FF}\right)$$

Equation S1.2.4(b♀)

$$K_{L2(t)}^{♂MM} = \rho_{L2(t)}^{♂MM}\left(1 - \delta_{L2(t)}^{♂MM}\right)$$

Equation S1.2.4(b♂)

$$T^{♀FF}_{L1(t)} = \rho^{♀FF}_{L1(t)}\delta^{♀FF}_{L1(t)}$$

Equation S1.2.4(c♀)

$$T^{♂MM}_{L1(t)} = \rho^{♂MM}_{L1(t)}\delta^{♂MM}_{L1(t)}$$

Equation S1.2.4(c♂)

$$L3^{♀FF}_{(t)} = L3^{♀FF}_{(t-1)}K^{♀FF}_{L3(t-1)} + L2^{♀FF}_{(t-1)}T^{♀FF}_{L2(t-1)}$$

Equation S1.2.5(a♀)

$$L3^{♂MM}_{(t)} = L3^{♂MM}_{(t-1)}K^{♂MM}_{L3(t-1)} + L2^{♂MM}_{(t-1)}T^{♂MM}_{L2(t-1)}$$

Equation S1.2.5(a♂)

$$K^{♀FF}_{L3(t)} = \rho^{♀FF}_{L3(t)}\left(1 - \delta^{♀FF}_{L3(t)}\right)$$

Equation S1.2.5(b♀)

$$K^{♂MM}_{L3(t)} = \rho^{♂MM}_{L3(t)}\left(1 - \delta^{♂MM}_{L3(t)}\right)$$

Equation S1.2.5(b♂)

$$T^{♀FF}_{L2(t)} = \rho^{♀FF}_{L2(t)}\delta^{♀FF}_{L2(t)}$$

Equation S1.2.5(c♀)

$$T^{♂MM}_{L2(t)} = \rho^{♂MM}_{L2(t)}\delta^{♂MM}_{L2(t)}$$

Equation S1.2.5(c♂)

$$L4^{♀FF}_{(t)} = L4^{♀FF}_{(t-1)}K^{♀FF}_{L4(t-1)} + L3^{♀FF}_{(t-1)}T^{♀FF}_{L3(t-1)}$$

Equation S1.2.6(a♀)

$$L4^{♂MM}_{(t)} = L4^{♂MM}_{(t-1)}K^{♂MM}_{L4(t-1)} + L3^{♂MM}_{(t-1)}T^{♂MM}_{L3(t-1)}$$

Equation S1.2.6(a♂)

$$K^{♀FF}_{L4(t)} = \rho^{♀FF}_{L4(t)}\left(1 - \delta^{♀FF}_{L4(t)}\right)$$

Equation S1.2.6(b♀)

$$K^{♂MM}_{L4(t)} = \rho^{♂MM}_{L4(t)}\left(1 - \delta^{♂MM}_{L4(t)}\right)$$

Equation S1.2.6(b♂)

$$T^{♀FF}_{L3(t)} = \rho^{♀FF}_{L4(t)}\delta^{♀FF}_{L4(t)}$$

Equation S1.2.6(c♀)

$$T^{♂MM}_{L3(t)} = \rho^{♂MM}_{L3(t)}\delta^{♂MM}_{L3(t)}$$

Equation S1.2.6(c♂)

L4 instars can then develop into pupae:

$$P^{♀FF}_{(t)} = P^{♀FF}_{(t-1)}K^{♀FF}_{P(t-1)} + L4^{♀FF}_{(t-1)}T^{♀FF}_{L4(t-1)}$$

Equation S1.2.7(a♀)

$$P^{♂MM}_{(t)} = P^{♂MM}_{(t-1)}K^{♂MM}_{P(t-1)} + L4^{♂MM}_{(t-1)}T^{♂MM}_{L4(t-1)}$$

Equation S1.2.7(a♂)

$P^{♀FF}_{(t)}$ and $P^{♂MM}_{(t)}$ are the number of pupae of the specified sex at time t. $K^{♀FF}_{P}$ and $K^{♂MM}_{P}$ are the proportion of pupae which did not grow sufficiently to transition to adults, while still surviving. $T^{♀FF}_{L4}$ and $T^{♂MM}_{L4}$ are the proportion of L4 larvae which successfully transition to pupae.

$$K_{P(t)}^{♀FF} = \rho_{P(t)}^{♀FF}\left(1 - \delta_{P(t)}^{♀FF}\right)$$
Equation S1.2.7(b♀)

$$K_{P(t)}^{♂MM} = \rho_{P(t)}^{♂MM}\left(1 - \delta_{P(t)}^{♂MM}\right)$$
Equation S1.2.7(b♂)

$$T_{L4(t)}^{♀FF} = \rho_{L4(t)}^{♀FF}\delta_{L4(t)}^{♀FF}$$
Equation S1.2.7(c♀)

$$T_{L4(t)}^{♂MM} = \rho_{L4(t)}^{♂MM}\delta_{L4(t)}^{♂MM}$$
Equation S1.2.7(c♂)

$\rho_P^{♀FF}$ and $\rho_P^{♂MM}$ is the survival probability given density dependence (see equations 9, 10, 11), while $\delta_{P(t)}^{♀FF}$ and $\delta_{P(t)}^{♂MM}$ are the probabilities of pupae developing into adults.

1.2.2. Aquatic Model: Density Dependent Population Regulation

Density-dependent population regulation (DDPR) limits the growth of mosquito populations[4]. In mosquitoes, DDPR occurs during the larval instar stages, where larval instars must compete for both space and resources. Competition therefore occurs between all larval instars (regardless of instar, genotype or sex). Different larval instars are different sizes and so consume different amounts of resources[5]. Total resource utilization depends on the number of larvae (and pupae) of all instars, genotypes and sexes.

$$R_{L1(t)} = \alpha_{L1}\left(L1_{(t-1)}^{♀SS} + L1_{(t-1)}^{♂SS} + L1_{(t-1)}^{♀RS} + L1_{(t-1)}^{♂RS} + L1_{(t-1)}^{♀RR} + L1_{(t-1)}^{♂RR}\right)$$
Equation S1.2.8(a)

$$R_{L2(t)} = \alpha_{L2}\left(L2_{(t-1)}^{♀SS} + L2_{(t-1)}^{♂SS} + L2_{(t-1)}^{♀RS} + L2_{(t-1)}^{♂RS} + L2_{(t-1)}^{♀RR} + L2_{(t-1)}^{♂RR}\right)$$
Equation S1.2.8(b)

$$R_{L3(t)} = \alpha_{L3}\left(L3_{(t-1)}^{♀SS} + L3_{(t-1)}^{♂SS} + L3_{(t-1)}^{♀RS} + L3_{(t-1)}^{♂RS} + L3_{(t-1)}^{♀RR} + L3_{(t-1)}^{♂RR}\right)$$
Equation S1.2.8(c)

$$R_{L4(t)} = \alpha_{L4}\left(L4_{(t-1)}^{♀SS} + L4_{(t-1)}^{♂SS} + L4_{(t-1)}^{♀RS} + L4_{(t-1)}^{♂RS} + L4_{(t-1)}^{♀RR} + L4_{(t-1)}^{♂RR}\right)$$
Equation S1.2.8(d)

Where α_{L1}, α_{L2}, α_{L3} and α_{L4} are the resource requirements for larvae of each instar category, which are in arbitrary units and account for both nutritional and space requirements. Mosquito population dynamics models including DDPR generally only consider larvae[2,4,6], however pupae may play a role in DDPR for example through space usage. We therefore include the capacity to allow for pupae to impact DDPR:

$$R_{P(t)} = \alpha_P\left(P_{(t-1)}^{♀SS} + P_{(t-1)}^{♂SS} + P_{(t-1)}^{♀RS} + P_{(t-1)}^{♂RS} + P_{(t-1)}^{♀RR} + P_{(t-1)}^{♂RR}\right)$$
Equation S1.2.8(e)

Pupae can be excluded from the DDPR calculation by simply setting α_P to 0.
The total amount of resources consumed is R_C.

$$R_{C(t)} = R_{L1(t)} + R_{L2(t)} + R_{L3(t)} + R_{L4(t)} + R_{P(t)}$$
Equation S1.2.8(f)

The value of $DDPR$ is therefore:

$$DDPR_{(t)} = \frac{1}{1 + \left(\frac{R_{C(t)}}{R_{T(t)}}\right)}$$

Equation S1.2.9

$R_{T(t)}$ is the total amount of resources available at time t and is a composite measure the total size of the breeding sites and the total amount of nutritional resources available. R_T is implemented as arbitrary "resource units". For simplicity, we assume an L4 instar requires 1 "resource unit" per day, and scale the resource usage of L1, L2 and L3 larval instars dependent on size (see Table S2.2 in Appendix 2). Unless otherwise stated simulations do not include pupae in the DDPR calculation. DDPR is used to modulate the natural survival probabilities. The daily survival probabilities of larvae (sex, genotype and instar) are therefore:

$$\rho_{L1(t)}^{♀FF} = DDPR_{(t)} * \gamma_{L1(t)}^{♀FF}$$

Equation S1.2.9(a♀)

$$\rho_{L1(t)}^{♂MM} = DDPR_{(t)} * \gamma_{L1(t)}^{♂MM}$$

Equation S1.2.9(a♂)

$$\rho_{L2(t)}^{♀FF} = DDPR_{(t)} * \gamma_{L2(t)}^{♀FF}$$

Equation S1.2.9(b♀)

$$\rho_{L2(t)}^{♂MM} = DDPR_{(t)} * \gamma_{L2(t)}^{♂MM}$$

Equation S1.2.9(b♂)

$$\rho_{L3(t)}^{♀FF} = DDPR_{(t)} * \gamma_{L3(t)}^{♀FF}$$

Equation S1.2.9(c♀)

$$\rho_{L3(t)}^{♂MM} = DDPR_{(t)} * \gamma_{L3(t)}^{♂MM}$$

Equation S1.2.9(c♂)

$$\rho_{L4(t)}^{♀FF} = DDPR_{(t)} * \gamma_{L4(t)}^{♀FF}$$

Equation S1.2.9(d♀)

$$\rho_{L4(t)}^{♂MM} = DDPR_{(t)} * \gamma_{L4(t)}^{♂MM}$$

Equation S1.2.9(d♂)

And for pupae:

$$\rho_{P(t)}^{♀FF} = DDPR_{(t)} * \gamma_{P(t)}^{♀FF}$$

Equation S1.2.9(e♀)

$$\rho_{P(t)}^{♂MM} = DDPR_{(t)} * \gamma_{P(t)}^{♂MM}$$

Equation S1.2.9(e♂)

When pupae are not affected by DDPR ($\alpha_P = 0$ or $R_P = 0$), then $DDPR$ in Equations S1.2.9(e) is set to 1 such that there is no DDPR impact on pupae.

1.3 Adult Male Module

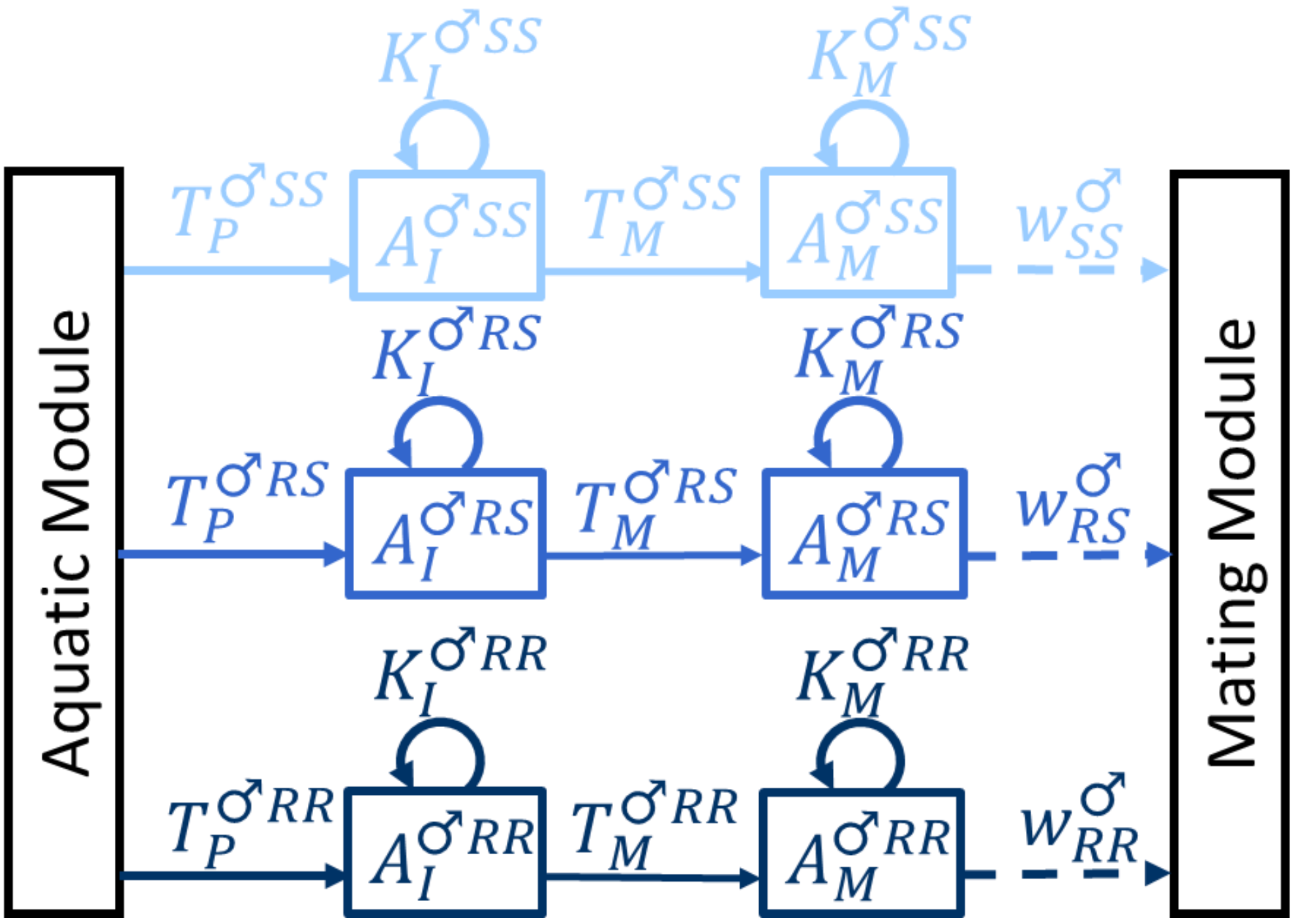

Fig D: Adult Male Module

Adult male mosquitoes emerge from the Aquatic Module as sexually immature (denoted by the I subscript, $A_I^{♂}$) and require a developmental period (approximately one day) for maturation (e.g., terminalia inversion [7]) before they become capable of mating. Sexually mature males (denoted by the M subscript, $A_M^{♂}$) become available to participate in the Mating Module (see section 1.5), where they mate with unmated adult females. The different genotypes are highlighted in different colours. ♂: denotes the male sex. S, R: Represent susceptible and resistant alleles. Genotypes (e.g., SS, SR, RR) refer to allele permutations. $A_I^{♂GG}$ and $A_M^{♂GG}$ are the number sexually immature or mature adult male mosquitoes with genotype GG, respectively. $T_P^{♂GG}$ and $T_M^{♂GG}$ are the proportions of males with genotype GG transitioning from pupae to immature adults and from immature to mature adults, respectively. $K_I^{♂GG}$ and $K_M^{♂GG}$ are the proportions of males with genotype GG that remain in the sexually immature or sexually mature state from one day to the next, respectively. $w_{MM}^{♂}$: is the mating competitiveness parameter of male with genotype GG (detailed in the Mating Module in section 1.5).

Male pupae emerge to give adult male mosquitoes. Adult male mosquitoes require a period of approximately one day for the inversion of the terminalia[7] before they are able to mate. The Male Module (Fig D) therefore has two life-stages of immature males, and sexually mature males.

The number of immature adult males (denoted by the I subscript) of each genotype on day t is:

$$A_{I(t)}^{♂MM} = \left(A_{I(t-1)}^{♂MM} K_{I(t)}^{♂MM}\right) + \left(P_{(t-1)}^{♂MM} T_{P(t)}^{♂MM}\right)$$

Equation S1.3(a)

$K_{I(t)}^{♂MM}$ is the proportion of immature males which have yet to develop into sexually mature males while still surviving. $T_{P(t)}^{♂MM}$ is the proportion of male pupae emerging.

$$T_{P(t)}^{♂MM} = \rho_{P(t)}^{♂MM} \delta_{p(t)}^{♂MM}$$

Equation S1.3(b)

$\rho_{P(t)}^{♂MM}$ is the daily survival probability of the pupae and $\delta_{p(t)}^{♂MM}$ is the daily probability of a pupa developing into an adult.

The number of sexually mature males at time t is:

$$A_{M(t)}^{♂MM} = \left(A_{M(t-1)}^{♂MM} K_{M(t)}^{♂MM}\right) + \left(A_{I(t-1)}^{♂MM} T_{M(t)}^{♂MM}\right)$$

Equation S1.3(c)

$K_{M(t)}^{♂MM}$ is the daily survival probability of male mosquitoes. $T_{M(t)}^{♂MM}$ is the proportion of the immature males which transition to being sexually mature.

The transition ($T_{M(t)}^{♂MM}$) and survival probabilities ($K_{I(t)}^{♂MM}$ and $K_{M(t)}^{♂MM}$) are dependent on the natural daily survival probabilities of males ($\rho_A^{♂MM}$) and insecticide exposure.

$$K_{I(t)}^{♂MM} = \left(\left(1-\varepsilon^{♂}\right)\rho_A^{♂MM}\left(1-\delta_A^{♂MM}\right)\right) + \left(\varepsilon^{♂}\rho_A^{♂MM}\left(1-\delta_A^{♂MM}\right)i_i^{♂MM}\right)$$

Equation S1.3(d)

$$K_{M(t)}^{♂MM} = \left(\left(1-\varepsilon^{♂}\right)\rho_A^{♂MM}\right) + \left(\varepsilon^{♂}\rho_A^{♂MM} i_i^{♂MM}\right)$$

Equation S1.3(e)

$$T_{M(t)}^{♂MM} = \left(\left(1-\varepsilon^{♂}\right)\rho_A^{♂MM}\delta_A^{♂MM}\right) + \left(\varepsilon^{♂}\rho_A^{♂MM}\delta_A^{♂MM} i_i^{♂MM}\right)$$

Equation S1.3(f)

During their lifespan, male mosquitoes may encounter insecticides, with survival outcomes dependent on their genotype. $\varepsilon^{♂}$ is the probability a male mosquito encounters an insecticide each day. $\rho_A^{♂MM}$ is the natural daily survival probability for male mosquitoes given the genotype in the absence of insecticide. $\delta_A^{♂MM}$ is the daily maturation probability of male mosquitoes given the genotype. $i_A^{♂MM}$ is the male insecticide survival probability given the genotype.

1.4 Adult Unmated Female Module

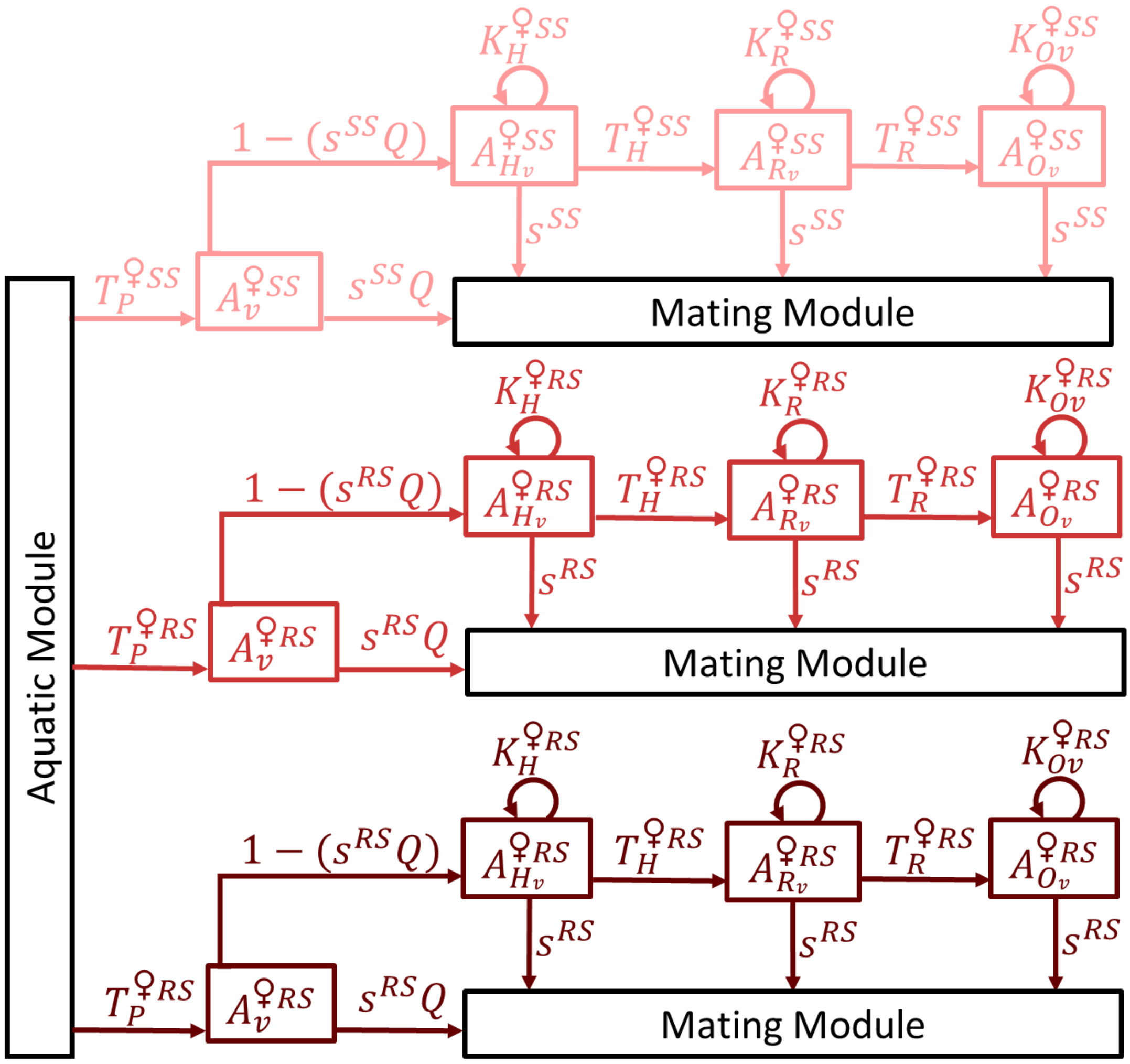

Fig E: Adult Unmated Female Module

Adult females emerge from pupae as unmated virgins (the subscript v denoting they are unmated, $A_v^{♀}$) and may mate before attempting to host seek and blood feed. Those not mating (or being unsuccessful in their mating) can attempt to host seek and blood feed. These unmated females then transition through the host-seeking ($A_{Hv}^{♀}$), resting ($A_{Rv}^{♀}$) (if blood-fed) to oviposition stages ($A_{Ov}^{♀}$), attempting to mate at each state. Unmated females in the oviposition state must mate before being able to lay eggs. The different genotypes and permutations are highlighted in different colours. ♀: Denote female sex. S, R: Represent susceptible and resistant alleles. Genotypes (e.g., SS, SR, RR) refer to allele combinations. $A_v^{♀GG}$, $A_{Hv}^{♀GG}$, $A_{Rv}^{♀GG}$, $A_{Ov}^{♀GG}$ are the number of females of genotype GG in virgin host-seeking, virgin resting, and virgin oviposition state, respectively. $T_P^{♀GG}$, $T_H^{♀GG}$, $T_R^{♀GG}$: Proportions of females with genotype GG transitioning from pupae to adults virgin, from adults virgin to host-seeking, form host-seek to resting, and from resting to oviposition state, respectively. S^{GG}: the swarming and mating success probability of females of the genotype GG. Q: biological preference to attempt to mate before an initial blood feed. $K_Y^{♀GG}$: proportions of females with genotype GG that remain in their current state Y to the next day (Example: $K_H^{♀RS}$: proportion of female with RS that remain in host-seeking state into the next day).

The overview of the Adult Unmated Female Module is given in Fig E. Adult females emerge from pupae. To produce viable offspring, female mosquitoes must mate with a male mosquito. We assume that female mosquitoes mate only once. We note that some female mosquitoes can successfully mate with multiple male mosquitoes, but this is generally infrequent [8]. Female mosquitoes who successfully mate transition to the Mated Female Module. Unmated females, which fail to mate, can start their host-seeking and blood-feeding behaviours, and attempt to mate the next day. Females may have a higher or lower preference for mating before an initial blood-feed, as some

mosquito species and/or populations may have a preference for having an initial blood feed before attempting to mate[9]. It is plausible this preference may impact resistance dynamics; which justifies the inclusion of this behaviour in the model.

The number of virgin female mosquitoes of each genotype emerging on day t is:

$$A_{V(t)}^{\female FF} = P_{(t-1)}^{\female FF} T_{P(t-1)}^{\female FF}$$

Equation S1.4(a)

$P_{(t-1)}^{\female FF}$ is the number of pupae and $T_P^{\female FF}$ is the proportion of those pupae successfully emerging.

$$T_{P(t)}^{\female FF} = \rho_{P(t)}^{\female FF} \delta_{p(t)}^{\female FF}$$

Equation S1.4(b)

Where $\rho_{P(t)}^{\female FF}$ is the survival probability of the pupae and $\delta_{p(t)}^{\female FF}$ is the pupal development probability. Virgin female mosquitoes may mate before or after an initial blood-feeding attempt. In the Unmated Female Module, we must therefore track the behavioural states of virgin females. We assume all female mosquitoes failing to mate still attempt to host-seek and blood-feed. The number of virgin females attempting to host-seek ($A_{Hv\,(t)}^{FF}$) is:

$$A_{Hv\,(t)}^{FF} = K_{H(t)}^{\female FF}\left(\left(1 - (s^{FF}Q)\right)A_{v(t)}^{FF}\right) + \left((1 - s^{FF})A_{Hv(t-1)}^{FF}\right)$$

Equation S1.4(c)

Q is the preference for mating before an initial attempt to blood-feed. Where Q = 1 is complete preference to attempt to mate before attempting to blood-feeding, and Q = 0 is the complete preference to mate after attempting to blood feed. Female mosquitoes successfully mate with a probability of s^{FF}, as mating success may be genotype dependent. Mating and host-seeking can occur on the same day. *Anopheles* mosquitoes generally swarm at dusk, with *Anopheles* mosquitoes then generally host-seeking and blood-feeding during the night. $K_H^{\female FF}$ is the proportion of virgin females which fail to mate and also fail to blood-feed, but survive to try again the next day:

$$K_{H(t)}^{\female FF} = \rho_H^{\female FF}\Bigg(\left(H\varepsilon_H^{\female FF} ZY(1 - f_{HI|net}^{\female FF})\, i_i^{\female FF}\right) + \left(H\varepsilon_H^{\female FF} Z(1-Y)(1 - f_{HI}^{\female FF})\right) + \left(H\varepsilon_H^{\female FF}(1-Z)(1 - f_{HO}^{\female FF})\right) + \left((1 - H)\varepsilon_A^{\female}(1 - f_A^{\female FF})\right) + \left(1 - \left(\left((1-H)\varepsilon_A^{\female}\right) + \left(H\varepsilon_H^{\female}\right)\right)\right)\Bigg)$$

Equation S1.4(d)

Where H is the proportion of host-seeking females attempting to find humans versus animals (anthropophily). And Z is the proportion of blood-feeds on humans which happen indoors (endophagy). This flexibility in biting behaviour allows the variation shown in mosquito populations to be considered[10]. $\varepsilon_A^{\female}$ is the probability of a host-seeking mosquito successfully finds an animal host. $\varepsilon_H^{\female}$ is the probability of a host-seeking mosquito successfully finds a human host. Y is the probability the indoor human host has and is using an ITN. $\rho_H^{\female FF}$ is the natural daily survival probability of host-seeking females given the genotype. $f_{HI|net}^{\female FF}$ is the probability a female mosquito feeds on a human host protected by an ITN. This calculated as estimated between the association between experimental hut survival and experimental hut feeding[11]. $f_{HI}^{\female FF}$ is the probability the female mosquito feeds on a human indoors that does not have an ITN. $f_{HO}^{\female FF}$ is the probability the female mosquito successfully feeds outdoors on a human host. $i_i^{\female FF}$ is the probability the mosquito survives the insecticide exposure given the resistance genotype. $\varepsilon_A^{\female FF}$ is the probability a female mosquito successfully encounters an animal host. $f_A^{\female FF}$ is the probability a female mosquito successfully feeds on an animal host.

Host-seeking females which successfully blood-feed move to the resting stage. The number of virgin resting female mosquitoes will be the number newly successfully blood-fed mosquitoes and the number still digesting the blood meal from prior successful feeding, and all these individuals once again failed to successfully mate with a male mosquito.

$$A_{Rv\,(t)}^{FF} = (1 - s^{FF})\left(\left(T_{H(t)}^{\female FF} A_{Hv(t-1)}^{FF}\right) + \left(K_{R(t)}^{\female FF} A_{R(t-1)}^{FF}\right)\right)$$

Equation S1.4(e)

$$T_{H(t)}^{♀FF} = \rho_{H(t)}^{♀FF}\left(\left(H\varepsilon_{H}^{♀FF}ZYf_{HI|net}^{♀FF}\,i_{i}^{♀FF}\right) + \left(H\varepsilon_{H}^{♀FF}Z(1-Y)f_{HI}^{♀FF}\right) + \left(H\varepsilon_{H}^{♀FF}(1-Z)f_{HO}^{♀FF}\right) + \left((1-H\,)\varepsilon_{A}^{♀}f_{A}^{♀FF}\right)\right)$$

Equation S1.4(f)

$$K_{R(t)}^{♀FF} = \left(\rho_{R}^{♀FF}\left(1-\ \delta_{R}^{♀FF}\right)\right)$$

Equation S1.4(g)

Once the blood meal has been successfully digested, the virgin females move to the oviposition state. Virgin females must successfully mate in order to lay viable egg; therefore females remain in this state until they successfully mate. The number in the oviposition state is the number which were in the oviposition state the previous day and have survived but still remain unmated, and the number of females who have now successfully digested the blood meal, and are now in the oviposition state who once again failed to mate.

$$A_{Ov\,(t)}^{FF} = (1-s^{FF})\left(\left(T_{R(t-1)}^{♀FF}A_{Rv(t-1)}^{FF}\right) + \left(K_{Ov(t-1)}^{♀FF}A_{Ov(t-1)}^{FF}\right)\right)$$

Equation S1.4(h)

The value of $K_{Ov}^{♀FF}$ is the daily survival probability of the female mosquitoes while in the resting state. This is because, until the mosquito has successfully mated she is unable to lay eggs, and the associated mortality risk associated with this.

$$K_{Ov(t)}^{♀FF} = \rho_{R(t)}^{♀FF}$$

Equation S1.4(i)

The value of $T_{R}^{♀FF}$ is the proportion of resting females which transition to the oviposition state, and have therefore successfully completed the digestion of the blood meal.

$$T_{R(t)}^{♀FF} = \rho_{R(t)}^{♀FF}\delta_{R(t)}^{♀FF}$$

Equation S1.4(j)

The value of $\rho_{Rv(t)}^{♀FF}$ is the daily survival probability.

1.5 Mating Module

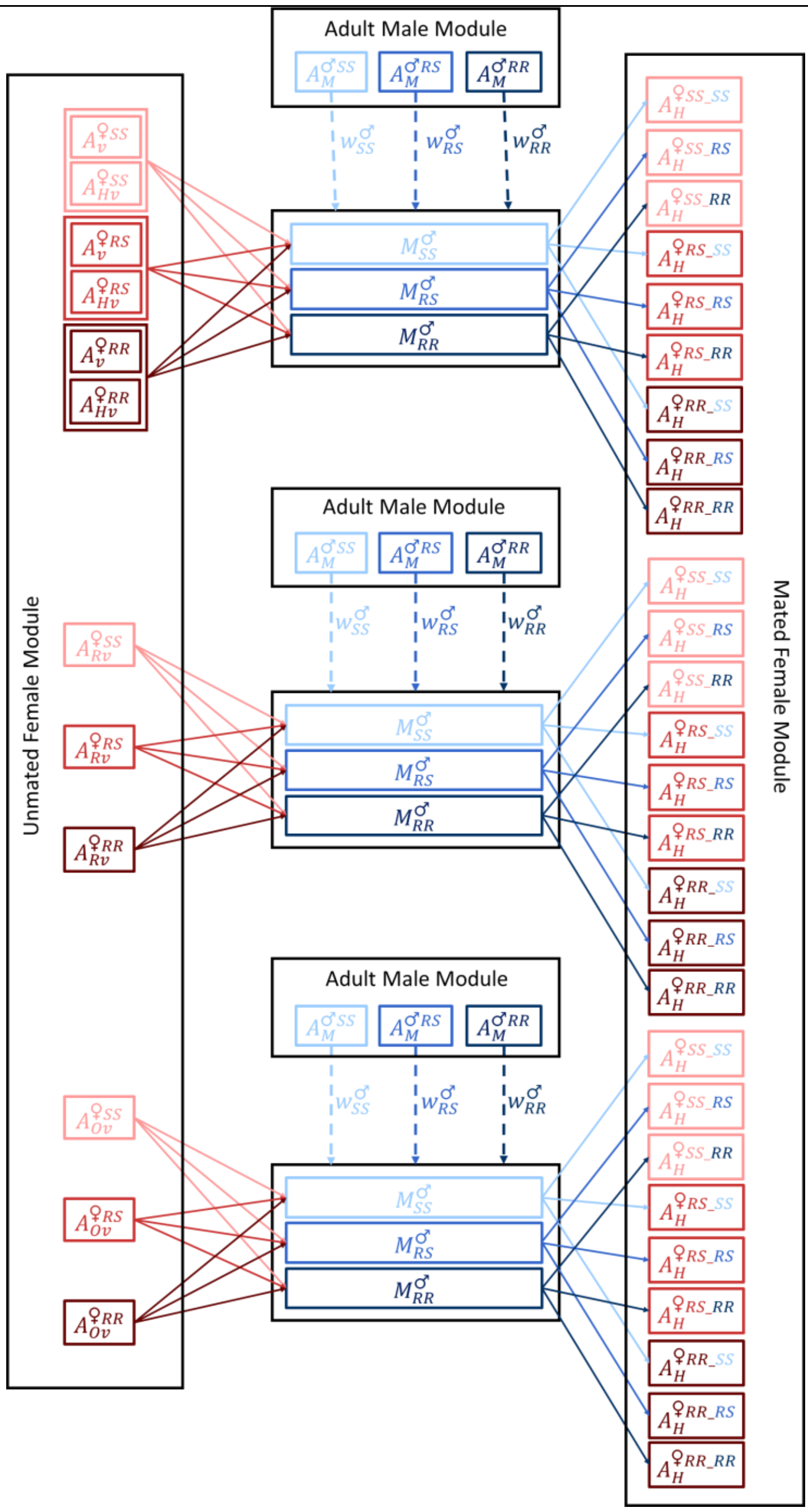

Fig F: Mating Module

In the Mating Module, unmated adult female mosquitoes can mate with mature adult males ($\boldsymbol{A_{\mathrm{M}}^{♂}}$) of any genotype. Male mating competitiveness may depend on genotype, as specified by $\boldsymbol{w_{\mathrm{MM}}^{♂}}$. Females that successfully mate transition to the Mated Female Module. The different genotypes and sex permutations are highlighted in different colours. ♀, ♂: Denote female and male sex, respectively. S, R: Represent susceptible and resistant alleles. Genotypes (e.g., SS, SR, RR) refer to allele permutations. $\boldsymbol{A_{\mathrm{M}}^{♂\mathrm{MM}}}$: the number of sexually mature adult male mosquitoes with genotype MM. $\mathbf{A}_{\boldsymbol{v}}^{♀\mathbf{FF}}$, $\mathbf{A}_{\boldsymbol{Hv}}^{♀\mathbf{FF}}$, $\mathbf{A}_{\boldsymbol{Rv}}^{♀\mathbf{FF}}$, $\mathbf{A}_{\boldsymbol{Ov}}^{♀\mathbf{FF}}$: the number of unmated females of genotype FF in virgin, host-seeking, resting, and oviposition state, respectively. $\mathbf{M}_{\mathbf{MM}}^{♂}$: proportion of matings for mature male mosquitoes with genotype MM. $\mathbf{A}_{\mathbf{H}}^{♀\mathbf{FF\_MM}}$, $\mathbf{A}_{\mathbf{R}}^{♀\mathbf{FF\_MM}}$, $\mathbf{A}_{\mathbf{O}}^{♀\mathbf{FF\_MM}}$: the number of mated females with genotype FF that mated with a male of genotype MM when they are in the host-seeking, resting, and oviposition state, respectively. (Example: if a female adult mosquito with genotype SS in host-seeking stage ($\boldsymbol{A_{Hv}^{♀SS}}$) mate with a mature male with genotype RR ($\mathbf{A}_{\mathbf{M}}^{♂\mathbf{RR}}$) she becomes $\boldsymbol{A_H^{♀SS\_RR}}$ and enters the Mated Female Module. $\boldsymbol{w_{\mathrm{MM}}^{♂}}$: is the mating competitiveness parameter of males with genotype MM.

A key part of the PopGEM model is to add mosquito mating (Fig F) into models of mosquito population dynamics. The number of mating pairings between males and females of different genotypes will be a function of both the mating competitiveness of the genotypes and the number of individuals of those genotypes.

We include a male mating competitiveness parameter ($w_{SS}^{♂}$, $w_{RS}^{♂}$ and $w_{RR}^{♂}$), which describes the mating competitiveness (relative to each genotype). This mating competitiveness accounts for competitiveness within swarms and also the ability to perform the swarming behaviour.

$$C_{MM(t)}^{♂} = A_{M(t)}^{♂MM} w_{MM}^{♂}$$

Equation S1.5(a)

This mating competitiveness needs to then be weighted by the number of males of each genotype to give the overall expected number of total matings by each genotype. These competitiveness-size values are then turned into proportions to give the expected proportion of the total matings by males of each genotype.

$$M_{MM(t)}^{♂} = \frac{C_{MM(t)}^{♂}}{\left(C_{SS(t)}^{♂} + C_{RS(t)}^{♂} + C_{RR(t)}^{♂}\right)}$$

Equation S1.5(b)

The values of $M_{SS}^{♂}$, $M_{RS}^{♂}$ and $M_{RR}^{♂}$ will be updated each day to account for changes in the population sizes of male mosquitoes. The values of $M_{SS}^{♂}$, $M_{RS}^{♂}$ and $M_{RR}^{♂}$ sum to one. We then calculate the number of female mosquitoes (of each genotype) which mate with each male genotype. Newly emerged virgin females which successfully mate immediately transition to the host-seeking state, meaning they can mate and host-seek on the same day.

$$A_{H\_new(t)}^{♀FF\_MM} = M_{MM(t)}^{♂} s_{FF}^{♀} \left(\left(A_{V(t)}^{♀FF} Q \right) + A_{H(t-1)}^{♀FF} \right)$$

Equation S1.5(c)

The value Q is the proportion of females which attempt to mate prior to host-seeking. The value $s_{FF}^{♀}$ is the mating success probability of female mosquitoes.

Mating by otherwise resting mosquitoes which newly mate will be:

$$A_{R\_new(t)}^{♀FF\_MM} = M_{MM(t)}^{♂} s_{FF}^{♀} A_{R(t-1)}^{♀FF}$$

Equation S1.5(d)

Mating by virgin females in the oviposition state which newly mate will be:

$$A_{O\_new(t)}^{♀FF\_MM} = M_{MM(t)}^{♂} s_{FF}^{♀} A_{O(t-1)}^{♀FF}$$

Equation S1.5(e)

1.6 Mated Female Module

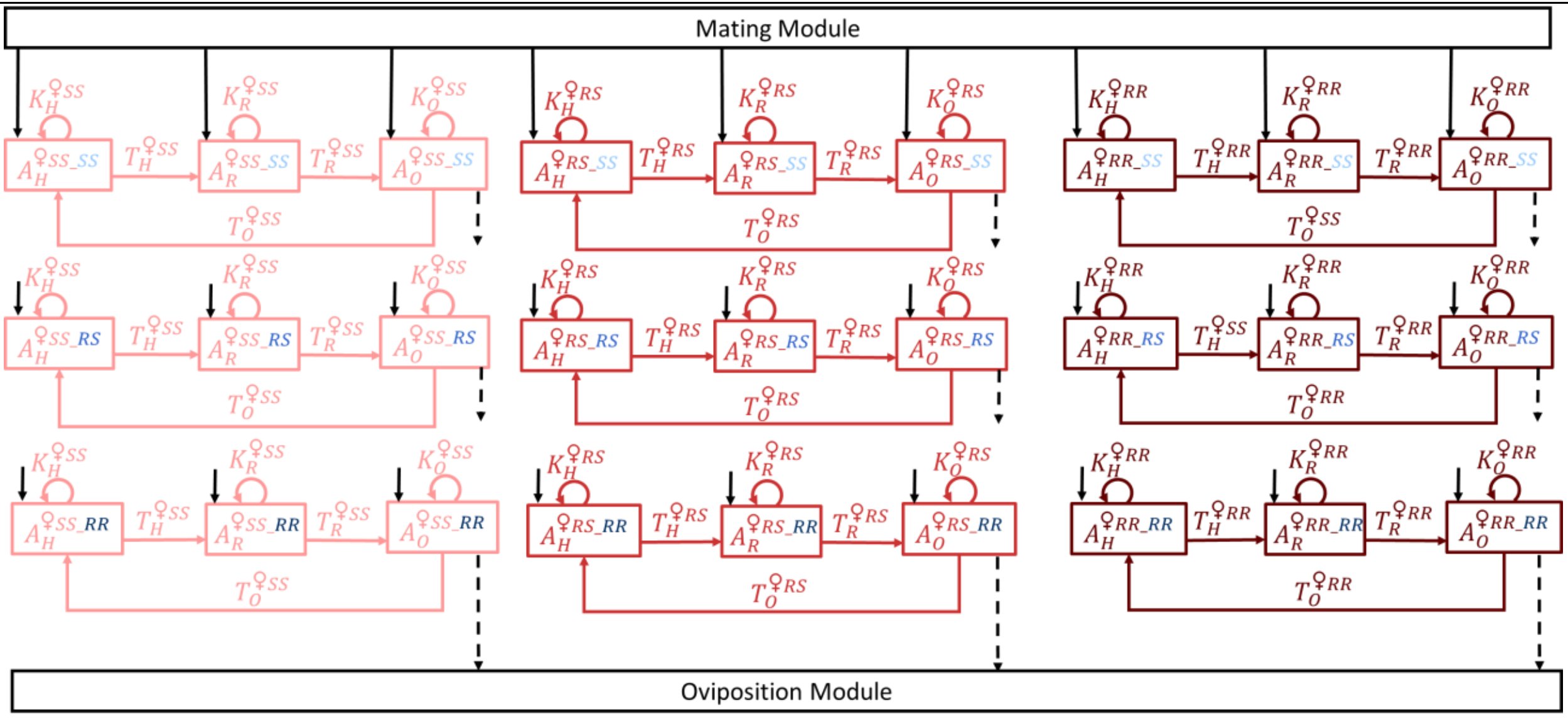

Fig G: Mated Female Module

Mated females (which come from the Mating Module, indicated by the solid black arrows) have one of nine possible mated genotypes. After mating, they transition through the host-seeking and blood-feeding ($\mathbf{A_H^{♀}}$), resting ($\mathbf{A_R^{♀}}$), and oviposition ($\mathbf{A_O^{♀}}$) stages. In the oviposition stage, females can lay eggs via interaction with the Oviposition Module (as indicated by the dashed black arrows). The different genotype permutations are highlighted in different colours. ♀: Denote female sex. S, R: Represent susceptible and resistant alleles. Genotypes (e.g., SS, SR, RR) refer to allele combinations. $\mathbf{A_H^{♀FF\_MM}}$, $\mathbf{A_R^{♀FF\_MM}}$, $\mathbf{A_O^{♀FF\_MM}}$: the number of mated females with genotype FF that mated with a male of genotype MM when they are in the host-seeking, resting, and oviposition state, respectively. $\mathbf{T_H^{♀FF}}$, $\mathbf{T_R^{♀FF}}$, $\mathbf{T_O^{♀FF}}$: Proportions of females with genotype FF transitioning from host-seek to resting state, from resting to oviposition state, and from oviposition to host-seek state, respectively. $\boldsymbol{K_Y^{♀FF}}$: proportions of females with genotype FF that remain in the current state Y (host-seeking, resting, oviposition) for one day.

The Mated Female Module (Fig G) is a modification of the Unmated Female Module, in that mating does not occur (female mosquitoes only mate once), and the mated females can lay eggs to complete the gonotrophic cycle, and so return to the host-seeking state to start the next gonotrophic cycle. In the Mated Female Module both the genotype of the female is tracked and the corresponding genotype of the male she mated with.

$$A_{H(t)}^{♀FF\_MM} = \left(K_{H(t-1)}^{♀FF}\left(A_{H(t-1)}^{♀FF\_MM} + A_{H\_new(t)}^{♀FF\_MM}\right)\right) + \left(T_{O(t-1)}^{♀FF}\left(A_{O(t)}^{♀FF\_MM} + A_{O\_new(t)}^{♀FF\_MM}\right)\right)$$

Equation S1.6(a)

$$A_{R(t)}^{♀FF\_MM} = \left(K_{R(t-1)}^{♀FF}\left(A_{R(t-1)}^{♀FF\_MM} + A_{R\_new(t)}^{♀FF\_MM}\right)\right) + \left(\left(A_{H(t-1)}^{♀FF\_MM} + A_{H\_new(t)}^{♀FF\_MM}\right)T_{H(t-1)}^{♀FF}\right)$$

Equation S1.6(b)

Females completing the digestion of the bloodmeal transition from the resting state to the oviposition state.

$$A_{O(t)}^{♀FF\_MM} = \left(T_{R(t-1)}^{♀FF}\left(A_{R(t-1)}^{♀FF\_MM} + A_{R\_new(t)}^{♀FF\_MM}\right)\right) + \left(\left(A_{O(t-1)}^{♀FF\_MM} + A_{O\_new(t)}^{♀FF\_MM}\right)K_{O(t-1)}^{♀FF}\right)$$

Equation S1.6(c)

Where $K_H^{♀FF}$, $T_H^{♀FF}$, $K_R^{♀FF}$ and $T_R^{♀FF}$ are calculated as previously described in equations S1.4(d), S1.4(f), S1.4(g) and S1.4(j) respectively.

$T_O^{♀FF}$ is the proportion of mated female mosquitoes in the oviposition state which successfully lay eggs and survive:

$$T_{O(t)}^{♀FF} = \rho_O^{♀FF}\ a_O^{♀FF}$$

Equation S1.6(d)

Where $a_O^{\female FF}$ is the probability of successfully ovipositing for the given genotype and $\rho_O^{\female FF}$ is the survival probability while in the oviposition stage for the given genotype.
$K_O^{\female FF}$ is the proportion of mated female mosquitoes in the oviposition state which fail to successfully oviposit while still surviving:

$$K_O^{\female FF} = \left(\rho_O^{\female FF}\left(1 - a_O^{\female FF}\right)\right)$$

Equation S1.6(e)

Once a female mosquito has laid eggs, she is able to start her next gonotrophic cycle, continuing this process until she eventually dies.

1.7 Blood-feeding Module

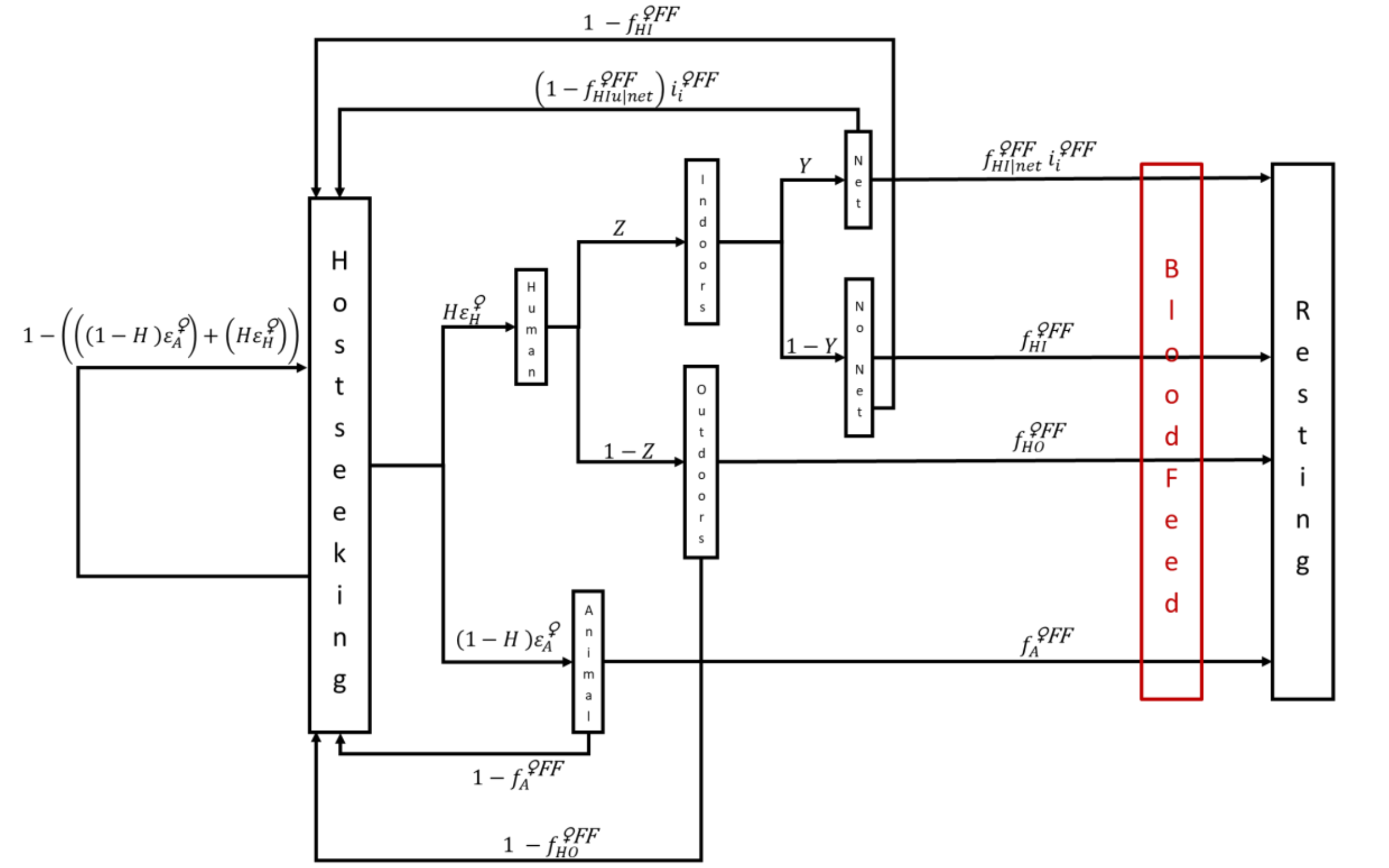

Fig H: Blood-Feeding Module

In the Blood-Feeding module female mosquitoes perform host-seeking (and can search for human or animal hosts), and humans can be either protected by an ITN or not. Blood-feeding success and survival when the human is using an ITN is dependent on the genotype of the female mosquito. From the host-seeking stage, the subset of female mosquitoes that actively pursue human hosts is represented by $H\varepsilon_H^{\female}$, where H is the degree of anthropophily, and $\varepsilon_H^{\female}$ is the daily probability a female mosquito successfully finds a human host. Among these, only a fraction attempt to feed indoors, which is accounted for by multiplying by Z, the probability that the human host is indoors during biting hours. Of those, another proportion is protected by ITNs, represented by multiplying again by Y, the probability that the host is using ITNs. At this point, the feeding and survival outcomes depend on two genotype-specific probabilities. The first, $f_{HI|net}^{\female FF}$, is the probability that a female mosquito with genotype FF successfully feeds on a host protected by a net. The second, $i_i^{\female FF}$, represents the probability that a mosquito with genotype FF survives exposure to insecticide. These two factors determine whether the mosquito dies from contact with ITNs or survives and transitions to the resting stage.

The blood-feeding module (Fig H) is used to describe the transition of adult female mosquitoes from the host-seeking to resting states in Equation S1,4(f), or host-seeking mosquitoes staying in the host-seeking state in equation S1.4(d).

1.8 Intervention Module

PopGEM allows the model to include (or not) the option of ITN retention and insecticide decay. The intervention module accounts for how interventions can change in coverage and efficacy over time. Coverage changes due to ITN retention, and efficacy declines due to insecticide decay.

We assume nets are retained following a Weibull distribution [12]. The exact values describing the shape of the Weibull distribution would be dependent on the specific net.

$$Y_{(t)} = Y\exp\left(-\left(\frac{tdeploy}{Halflife_{retention}}\right)^{shape_{net}}\right)\log(2)$$

Equation S1.7

The value of $Y_{(t)}$ therefore replaces the value of Y (the original deployed ITN coverage) in the equations. Where $tdeploy$ is the time (in days) since the last round of ITN deployments. $Halflife_{retention}$ is the half-life in days of the net retention, and $shape_{net}$ is a shape parameter which describes the shape of the Weibull distribution.

Likewise, we allow insecticides to decay over time, and as such their effectiveness at killing mosquitoes declines also. Given we only consider the efficacy (and not the concentration) we also consider a Weibull distribution; but note other assumptions could be made, as different insecticides may decay at different rates and this may also be affected by the physical net properties too.

$$i_{i(t)}^{♀FF} = i_i^{♀FF}\exp\left(-\left(\frac{tdeploy}{Halflife_i}\right)^{shape_i}\right)\log(2)$$

Equation 1.8(a)

And note the blood-feeding success ($f_{HI|net}^{♀FF}$) will also update according to the current survival to the insecticide. $Halflife_i$ is the half-life of the insecticide efficacy in days, and $shape_i$ is the shape parameter of the Weibull distribution. And the equivalent for male mosquitoes:

$$i_{i(t)}^{♂MM} = i_i^{♂MM}\exp\left(-\left(\frac{tdeploy}{Halflife_i}\right)^{shape_i}\right)\log(2)$$

Equation 1.8(b)

Appendix 2: State Variables and Parameters in PopGEM and Their Descriptions

The tables below list the state variables and parameters used in the PopGEM model across its modules. State variables are calculated internally through daily iterations of the model, while parameters are fixed constants based on standard assumptions in IR modelling or sourced from existing literatures. Parameters/state variables are included in the tables in the order where they are first introduced in the model equations.

Table A: State Variables and Parameters in Oviposition Module

**Oviposition Module**				
**Symbol**	**Description**	**Type**	**Value**	**Rational**
$F_{E(t)}^{♀FF\_MM}$	Total number of female eggs laid by females with the genotype FF which mated with males of the genotype MM on day t.	State variable	N/A	N/A

$F_{E(t)}^{\male FF\_MM}$	Total number of male eggs laid by females with the genotype FF which mated with males of the genotype MM on day t.	State variable	N/A	N/A
$A_{O(t)}^{\female FF\_MM}$	the total number of females with the genotype FF who mated with males with a genotype MM attempting to lay eggs on day t.	State variable	N/A	N/A
$\theta_{(t)}^{FF}$	The average number of eggs laid per female with the genotype FF. Can vary with climatic conditions (see Appendix 4).	State variable	N/A	N/A
Φ^{FF}	The sex-ratio of the eggs laid by females with the genotype FF.	Parameter	0·5	Standard assumption in mosquito population models.
$a_o^{\female FF}$	The daily oviposition success probability of females with the genotype FF.	Parameter	1	We assume all females which attempt to oviposit are successful.
$N_{E(t)}^{\female SS}$, $N_{E(t)}^{\female RS}$,	The number of female eggs laid of each genotype on day t.	State variable	N/A	N/A
$N_{E(t)}^{\male SS}$, $N_{E}^{\male RS}$, $N_{E(t)}^{\male RR}$	The number of male eggs laid of each genotype on day t.	State variable	N/A	N/A

Table B: State Variables and Parameters in Aquatic Module

**Aquatic Module**				
**Symbol**	**Description**	**Type**	**Value**	**Rational**
$E_{(t)}^{\female FF}$	The number of female eggs of genotype FF on day t.	State variable	N/A	N/A
$K_E^{\female FF}$	The proportion of female eggs of genotype FF from the previous day which have not hatched, but still remain alive.	State variable	N/A	N/A
$E_{(t)}^{\male MM}$	The number of male eggs of genotype MM on day t.	State variable	N/A	N/A
$K_E^{\male MM}$	The proportion of male eggs of genotype MM from the previous day which have not hatched, but still remain alive.	State variable	N/A	N/A
$\rho_{E(t)}^{\female FF}$	The daily survival probability of female eggs of genotype FF. Can depend on temperature (see Appendix 4).	Parameter	0·67	Estimate from [13]
$h_{E(t)}^{\female FF}$	Embryony, the hatch probability (1/number of days required to hatch) for female eggs of the genotype FF. Can depend on temperature (see Appendix 4).	Parameter	0·6756757	Estimate from [14]
$\rho_{E(t)}^{\male MM}$	the daily survival probability of male eggs of genotype MM, and can be dependent on temperature.	Parameter	0·67	Estimate from [13]
$h_{E(t)}^{\male MM}$	Embryony, hatch probability (1/number of days required to hatch) for male eggs of the genotype MM, and can be dependent on temperature.	Parameter	0·6756757	Estimate from [14]
$L1_{(t)}^{\female FF}$	The number of female L1 larvae of the specified genotype (FF) on day t.	State variable	N/A	N/A
$L1_{(t)}^{\male MM}$	The number of male L1 larvae of the specified genotype (MM) on day t.	State variable	N/A	N/A
$K_{L1}^{\female FF}$	The proportion of female L1 larvae of genotype FF who do not transition to L2, but survive.	State variable	N/A	N/A
$K_{L1}^{\male MM}$	The proportion of male L1 larvae of genotype MM who do not transition to L2, but survive.	State variable	N/A	N/A
$T_E^{\female FF}$	The proportion of female eggs of the genotype FF transitioning to L1 larvae.	State variable	N/A	N/A
$T_E^{\male MM}$	The proportion of male eggs of the genotype MM transitioning to L1 larvae.	State variable	N/A	N/A
$\rho_{L1(t)}^{\female FF}$	Daily survival probability of female L1 larvae given the genotype FF. Can depend on temperature (see Appendix 4) and density dependence.	State variable	N/A	N/A
$\delta_{L1(t)}^{\female FF}$	The daily growth probability of female L1 larvae of genotype FF. Can depend on temperature (see Appendix 4).	State variable	N/A	N/A
$\rho_{L1(t)}^{\male MM}$	Daily survival probability of male L1 larvae given the genotype MM. Can depend on temperature (see Appendix 4) and density dependence.	State variable	N/A	N/A
$\delta_{L1(t)}^{\male MM}$	The daily growth rate of male L1 larvae of genotype MM. Can depend on temperature (see Appendix 4).	State variable	N/A	N/A
$L2_{(t)}^{\female FF}$	The number of female L2 larvae of the specified genotype (FF) on day t.	State variable	N/A	N/A
$L2_{(t)}^{\male MM}$	The number of male L2 larvae of the specified genotype (MM) on day t.	State variable	N/A	N/A
$K_{L2}^{\female FF}$	The proportion of female L2 larvae of genotype FF who do not transition to L3, but survive.	State variable	N/A	N/A

$K_{L2}^{♂MM}$	The proportion of male L2 larvae of genotype MM who do not transition to L3, but survive.	State variable	N/A	N/A
$T_{L1}^{♀FF}$	The proportion of female L1 larvae of the genotype FF transitioning to L2 larvae.	State variable	N/A	N/A
$T_{L1}^{♂MM}$	The proportion of male L1 larvae of the genotype MM transitioning to L2 larvae.	State variable	N/A	N/A
$\rho_{L2(t)}^{♀FF}$	Daily survival probability of female L2 larvae given the genotype FF. Can depend on temperature (see Appendix 4) and density dependence.	State variable	N/A	N/A
$\rho_{L2(t)}^{♂MM}$	Daily survival probability of male L2 larvae given the genotype MM. Can depend on temperature (see Appendix 4) and density dependence.	State variable	N/A	N/A
$\delta_{L2(t)}^{♀FF}$	The daily growth rate of female L2 larvae of genotype FF, dependent on temperature.	State variable	N/A	N/A
$\delta_{L2(t)}^{♂MM}$	The daily growth rate of male L2 larvae of genotype MM. Can depend on temperature (see Appendix 4).	State variable	N/A	N/A
$L3_{(t)}^{♀FF}$	The number of female L3 larvae of the specified genotype (FF) on day t.	State variable	N/A	N/A
$L3_{(t)}^{♂MM}$	The number of male L3 larvae of the specified genotype (MM) on day t.	State variable	N/A	N/A
$K_{L3}^{♀FF}$	The proportion of female L3 larvae of genotype FF who do not transition to L4, but survive.	State variable	N/A	N/A
$K_{L3}^{♂MM}$	The proportion of male L3 larvae of genotype MM who do not transition to L4, but survive.	State variable	N/A	N/A
$T_{L2}^{♀FF}$	The proportion of female L2 larvae of the genotype FF transitioning to L3 larvae.	State variable	N/A	N/A
$T_{L2}^{♂MM}$	The proportion of male L2 larvae of the genotype MM transitioning to L3 larvae.	State variable	N/A	N/A
$\rho_{L3(t)}^{♀FF}$	Daily survival probability of female L3 larvae given the genotype FF. Can depend on temperature (see Appendix 4) and density dependence.	State variable	N/A	N/A
$\rho_{L3(t)}^{♂MM}$	Daily survival probability of male L3 larvae given the genotype MM. Can depend on temperature (see Appendix 4) and density dependence.	State variable	N/A	N/A
$\delta_{L3(t)}^{♀FF}$	The daily growth rate of female L3 larvae of genotype FF. Can depend on temperature (see Appendix 4).	State variable	N/A	N/A
$\delta_{L3(t)}^{♂MM}$	The daily growth rate of male L3 larvae of genotype MM, dependent on temperature.	State variable	N/A	N/A
$L4_{(t)}^{♀FF}$	The number of female L4 larvae of the specified genotype (FF) on day t.	State variable	N/A	N/A
$L4_{(t)}^{♂MM}$	The number of male L4 larvae of the specified genotype (MM) on day t.	State variable	N/A	N/A
$K_{L4}^{♀FF}$	The proportion of female L4 larvae of genotype FF who do not transition to pupae, but survive.	State variable	N/A	N/A
$K_{L4}^{♂MM}$	The proportion of male L4 larvae of genotype MM who do not transition to pupae, but survive.	State variable	N/A	N/A
$T_{L3}^{♀FF}$	The proportion of female L3 larvae of the genotype FF transitioning to L4 larvae.	State variable	N/A	N/A
$T_{L3}^{♂MM}$	The proportion of male L3 larvae of the genotype MM transitioning to L4 larvae.	State variable	N/A	N/A
$\rho_{L4(t)}^{♀FF}$	Daily survival probability of female L4 larvae given the genotype FF. Can depend on temperature (see Appendix 4) and density dependence.	State variable	N/A	N/A
$\rho_{L4(t)}^{♂MM}$	Daily survival probability of male L4 larvae given the genotype MM. Can depend on temperature (see Appendix 4) and density dependence.	State variable	N/A	N/A
$\delta_{L4(t)}^{♀FF}$	The daily growth rate of female L4 larvae of genotype FF. Can depend on temperature (see Appendix 4).	State variable	N/A	N/A
$\delta_{L4(t)}^{♂MM}$	The daily growth rate of male L4 larvae of genotype MM. Can depend on temperature (see Appendix 4).	State variable	N/A	N/A
$P_{(t)}^{♀FF}$	The number of female pupae of the specified genotype (FF) on day t.	State variable	N/A	N/A
$P_{(t)}^{♂MM}$	The number of male pupae of the specified genotype (MM) on day t.	State variable	N/A	N/A
$K_{P}^{♀FF}$	The proportion of female pupae of genotype FF who do not emerge as adults, but survive.	State variable	N/A	N/A
$K_{P}^{♂MM}$	The proportion of male pupae of genotype MM who do not emerge as adults, but survive.	State variable	N/A	N/A
$T_{L4}^{♀FF}$	The proportion of female L4 larvae of the genotype FF transitioning to pupae.	State variable	N/A	N/A
$T_{L4}^{♂MM}$	The proportion of male L4 larvae of the genotype MM transitioning to pupae.	State variable	N/A	N/A

$\rho_{P(t)}^{♀FF}$	Daily survival probability of female pupae given the genotype FF. Can depend on temperature (see Appendix 4) and density dependence.	State variable	N/A	N/A
$\rho_{P(t)}^{♂MM}$	Daily survival probability of male pupae given the genotype MM. Can depend on temperature (see Appendix 4) and density dependence.	State variable	N/A	N/A
$\delta_{P(t)}^{♀FF}$	The daily growth rate of female pupae of genotype FF. Can depend on temperature (see Appendix 4).	State variable	N/A	N/A
$\delta_{P(t)}^{♂MM}$	The daily growth rate of male pupae of genotype MM. Can depend on temperature (see Appendix 4).	State variable	N/A	N/A
R_{L1}	Total "resource unit" utilisation by all L1 larvae.	State variable	N/A	N/A
R_{L2}	Total "resource unit" utilisation by all L2 larvae.	State variable	N/A	N/A
R_{L3}	Total "resource unit" utilisation by all L3 larvae.	State variable	N/A	N/A
R_{L4}	Total "resource unit" utilisation by all L4 larvae.	State variable	N/A	N/A
R_P	Total "resource unit" utilisation by all pupae.	State variable	N/A	N/A
α_{L1}	Average "resource unit" utilisation by a single L1 larvae.	Parameter	0·2084118	Estimate from [15]. Scaled around L4.
α_{L2}	Average "resource unit" utilisation by a single L2 larvae.	Parameter	0·3179535	Estimate from [15]. Scaled around L4.
α_{L3}	Average "resource unit" utilisation by a single L3 larvae.	Parameter	0·5794099	Estimate from [15]. Scaled around L4.
α_{L4}	Average "resource unit" utilisation by a single L4 larvae.	Parameter	1	Estimate from [15]. Scaled around L4.
α_P	Average "resource unit" utilisation by a single pupae.	Parameter	0	Pupae usually not included in density dependence calculations[4]. By default, they are not included.
R_C	Total "resource unit" utilisation by all larvae and pupae.	State variable	N/A	N/A
$DDPR$	The value of the density dependent population regulation.	State variable	N/A	N/A
$R_{T(t)}$	The total "resource units" available on day t.	State variable	N/A	N/A
$\gamma_{L1(t)}^{♀FF}$	Temperature dependent survival probability for female L1 larvae with genotype FF.	State variable	N/A	N/A
$\gamma_{L1(t)}^{♂MM}$	Temperature dependent survival probability for male L1 larvae with genotype MM.	State variable	N/A	N/A
$\gamma_{L2(t)}^{♀FF}$	Temperature dependent survival probability for female L2 larvae with genotype FF.	State variable	N/A	N/A
$\gamma_{L2(t)}^{♂MM}$	Temperature dependent survival probability for male L2 larvae with genotype MM.	State variable	N/A	N/A
$\gamma_{L3(t)}^{♀FF}$	Temperature dependent survival probability for female L3 larvae with genotype FF.	State variable	N/A	N/A
$\gamma_{L3(t)}^{♂MM}$	Temperature dependent survival probability for male L3 larvae with genotype MM.	State variable	N/A	N/A
$\gamma_{L4(t)}^{♀FF}$	Temperature dependent survival probability for female L4 larvae with genotype FF.	State variable	N/A	N/A
$\gamma_{L4(t)}^{♂MM}$	Temperature dependent survival probability for male L4 larvae with genotype MM.	State variable	N/A	N/A
$\gamma_{P(t)}^{♀FF}$	Temperature dependent survival probability for female pupae with genotype FF.	State variable	N/A	N/A
$\gamma_{P(t)}^{♂MM}$	Temperature dependent survival probability for male pupae with genotype MM.	State variable	N/A	N/A

Table C: State Variables and Parameters in Adult Male Module

Adult Male Module				
**Symbol**	**Description**	**Type**	**Value**	**Rational**
$A_{I(t)}^{♂MM}$	The number of sexually immature adult males of the genotype MM on day t.	State variable	N/A	N/A
$K_{I}^{♂MM}$	The proportion of sexually immature males of surviving to the next day while remaining sexually immature.	State variable	N/A	N/A
$T_{P(t)}^{♂MM}$	The proportion of male pupae of the genotype MM emerging and becoming adult males.	State variable	N/A	N/A
$A_{M(t)}^{♂MM}$	The number of sexually mature adult males of the genotype MM on day t.	State variable	N/A	N/A
$K_{M(t)}^{♂MM}$	The proportion of sexually mature adult males of the genotype MM surviving to the next day.	State variable	N/A	N/A
$T_{M(t)}^{♂MM}$	The proportion of sexually immature males of genotype MM surviving and reaching sexual maturity.	State variable	N/A	N/A
$\varepsilon^{♂}$	The probability a male mosquito encounters the insecticide each day. We assume that this approximately equivalent to their house entry probabilities.	Parameter	0·933177*Z	Estimate from [16]. Evaluated as part of parameter space testing.
$\rho_{A}^{♂MM}$	The natural daily survival probability for male mosquitoes given the genotype. Can depend on temperature (see Appendix 4).	State variable	N/A	N/A
$\delta_{A}^{♂MM}$	Is the sexual maturation rate of the male mosquitoes given the genotype.	Parameter	1	Estimate from [17].
$i_{i}^{♂MM}$	Is the male insecticide survival probability given the genotype.	Parameter	$\left(\beta_1 i_{A}^{♀FF}\right) + \beta_2$	Appendix 3
β_1	Regression coefficient for male-female insecticide conversion.	Parameter	0·67533	Appendix 3 Evaluated as part of parameter space testing.
β_2	Regression intercept for male-female insecticide conversion.	Parameter	0·09425	Appendix 3

Table D: State Variables and Parameters in Unmated Female Module

Unmated Female Module				
**Symbol**	**Description**	**Type**	**Value**	**Rational**
$A_{V(t)}^{♀FF}$	The number of newly emerged virgin females of genotype FF.	State variable	N/A	N/A
$T_{P}^{♀FF}$	The proportion of female pupae of the genotype FF emerging and becoming adult females.	State variable	N/A	N/A
$A_{Hv\,(t)}^{FF}$	The number of host-seeking virgin females on day t of the genotype FF.	State variable	N/A	N/A
$\rho_{H}^{♀FF}$	The daily natural survival probability of host-seeking female mosquitoes.	State variable	N/A	N/A
H	The degree of anthropophily, and is therefore the proportion of host-seeking females which will attempt to feed on humans.	Parameter	0-1	Extends the full range from complete range from exclusive zoophily to exclusive anthropophily.
$\varepsilon_{A}^{♀}$	The probability of a zoophilic host-seeking mosquito successfully finding an animal host.	Parameter	0-1	Evaluated as part of parameter space testing.
Z	The probability of the human host being indoors.	Parameter	0-1	Evaluated as part of parameter space testing.
Y	The probability the human host has an ITN.	Parameter	0-1	Evaluated as part of parameter space testing.
$\varepsilon_{H}^{♀}$	The probability of an anthropophilic host-seeking mosquito successfully finding a human host.	Parameter	0-1	Evaluated as part of parameter space testing.
$A_{Rv\,(t)}^{FF}$	The number of resting virgin females on day t of genotype FF.	State variable	N/A	N/A
s^{FF}	The swarming and mating success probability of females of the genotype FF.	Parameter	0-1	Evaluated as part of parameter space testing.
Q	Biological preference to attempt to mate before an initial blood feed.	Parameter	0·49	Estimate from [9]. Evaluated as part of parameter space testing.

$T_{Hv}^{♀FF}$	The probability a virgin female mosquito transitions from the hostseeking to resting state given they have the genotype FF.	State variable	N/A	N/A
$K_{Rv}^{♀FF}$	The probability a female mosquito remains in the resting state given the genotype FF.	State variable	N/A	N/A
$f_{HI\|net}^{♀FF}$	The probability the female mosquito feeds on a human host protected by an ITN given the genotype.	Parameter	0-1	Evaluated as part of parameter space testing.
$f_{HI}^{♀FF}$	The probability the female mosquito feeds on a human indoors that does not have an ITN given the genotype.	Parameter	0-1	Evaluated as part of parameter space testing.
$f_{HO}^{♀FF}$	The probability the female mosquito successfully feeds outdoors on a human host given the genotype.	Parameter	0-1	Evaluated as part of parameter space testing.
$f_{A}^{♀FF}$	The probability a female mosquito successfully feeds on an animal host given the genotype.	Parameter	0-1	Evaluated as part of parameter space testing.
$i_{i}^{♀FF}$	The survival probability of a female when encountering insecticide i given the genotype.	Parameter	0-1	Evaluated as part of parameter space testing.
$\delta_{R}^{♀FF}$	The rate of egg maturation (1/gonotrophic cycle length).	State variable	N/A	N/A
$\rho_{R}^{♀FF}$	The natural daily survival probability while resting for adult female mosquitoes.	State variable	N/A	N/A
$A_{Ov\,(t)}^{FF}$	The number of virgin females of genotype FF on day t in the oviposition stage who cannot lay eggs until mated.	State variable	N/A	N/A
$T_{Rv}^{♀FF}$	The probability of transitioning from the resting stage to the oviposition stage.	State variable	N/A	N/A
$K_{Ov}^{♀FF}$	The probability a virgin female remains in the oviposition state given the genotype FF.	State variable	N/A	N/A

Table E: State Variables and Parameters in Mating Female Module

Mating Module				
**Symbol**	**Description**	**Type**	**Value**	**Rational**
$w_{SS}^{♂}$, $w_{RS}^{♂}$ and $w_{RR}^{♂}$	Relative mating competitiveness of the specified genotype.	Parameters	1	No fitness costs are included in the present simulations.
$C_{SS}^{♂}$, $C_{RS}^{♂}$, $C_{RR}^{♂}$	Mating competitiveness weighted population size of the male mosquito populations.	State variable	N/A	N/A
$C_{MM}^{♂}$	Mating competitiveness weighted population size of male mosquitoes with the genotype MM.	State variable	N/A	N/A
$M_{MM}^{♂}$	Proportion of matings by the specified male genotype.	State variable	N/A	N/A
$M_{SS}^{♂}$, $M_{RS}^{♂}$, $M_{RR}^{♂}$	Proportion of matings by each male genotype.	State variable	N/A	N/A

Table F: State Variables and Parameters in Mated Female Module

Mated Female Module				
**Symbol**	**Description**	**Type**	**Value**	**Rational**
$A_{H\_new(t)}^{♀FF\_MM}$	The number of newly mated females of genotype FF who mated with a male of genotype MM on day t who are in the hostseeking state.	State variable	N/A	N/A
$A_{R\_new(t)}^{♀FF\_MM}$	The number of newly mated females of genotype FF who mated with a male of genotype MM on day t who are in the resting state.	State variable	N/A	N/A
$A_{O\_new(t)}^{♀FF\_MM}$	The number of newly mated females of genotype FF who mated with a male of genotype MM on day t who are in the oviposition state.	State variable	N/A	N/A
$A_{H(t)}^{♀FF\_MM}$	The number of mated females of genotype FF who mated with a male of genotype MM on day t who are in the hostseeking state.	State variable	N/A	N/A
$A_{R(t)}^{♀FF\_MM}$	The number of mated females of genotype FF who mated with a male of genotype MM on day t who are in the resting state.	State variable	N/A	N/A
$A_{O(t)}^{♀FF\_MM}$	The number of mated females of genotype FF who mated with a male of genotype MM on day t who are in the oviposition state.	State variable	N/A	N/A
$K_{H}^{♀FF}$	The probability of a female mosquito remaining in the hostseeking state given the genotype FF.	State variable	N/A	N/A
$K_{R}^{♀FF}$	The probability of a female mosquito remaining in the resting state given the genotype FF.	State variable	N/A	N/A
$T_{O}^{♀FF}$	The probability a female mosquito transitions from the oviposition state back to the hostseeking state given they have the genotype FF.	State variable	N/A	N/A
$T_{H}^{♀FF}$	The probability a female mosquito transitions from the hostseeking to resting state given they have the genotype FF.	State variable	N/A	N/A
$T_{R}^{♀FF}$	the probability of transitioning from the resting stage to the oviposition stage.	State variable	N/A	N/A
$\rho_{O}^{♀FF}$	The probability of surviving while in the oviposition state for a female mosquito with the genotype FF.	State variable	N/A	N/A

Appendix 3: Parameter Estimation

The parameters listed below were used in two sensitivity analyses assessing: (1) the impact of PYR-ITNs on IR and malaria vector control, and (2) the impact of NGM-ITNs on the same outcomes. For clarity, the following symbols are used throughout the table: ♀ and ♂ denote female and male mosquitoes, respectively; FF and MM represent female and male genotype pairs, which may be RR, SS, or RS; E refers to eggs; L1 through L4 indicate larval stages 1 to 4; and P denotes the pupal stage. Behavioural states are abbreviated as follows: O for oviposition, R for resting, and H for host-seeking. Virgin behavioural states are represented as Ov (ovipositing virgin), Rv (resting virgin), and Hv (host-seeking virgin). Location-specific host-seeking behaviour is indicated by HO for outdoor and HI for indoor. *t* denotes time, expressed as the nth day of the simulation.

Table G: State Variables and Parameters Used in the Sensitivity Analysis

Name	Type	Value	Rational
Starting population values (stage-state-sex-genotype)	State variable	Calculated for each randomly sampled parameter combination.	The steady state equilibrium value in the absence of interventions is used to determine the starting number, which are distributed between the genotypes as specified by the starting resistance frequency ratio.
Egg batch size	Parameter	187 female^{-1}	Estimates from [18] with modification to the maximum temperature through [19].
Egg sex ratio	Parameter	0·5	Default assumption of most models.
Probability successful oviposition	Parameter	1 day^{-1}	Assume all females successfully oviposit because the availability of larval habitats is high due to population equilibrium
Egg survival probability	Parameter	0·9888291 day^{-1}	Assumed same as for L1 larvae.
L1 survival probability.	Parameter	0·9888291 day^{-1}	Survival probability from[13] under the optimal temperature conditions as calculated from [19]. This is the maximum survival probability excluding density dependence.
L2 survival probability.	Parameter	0·9005225 day^{-1}	Survival probability from[13] under the optimal temperature conditions as calculated from [19]. This is the maximum survival probability excluding density dependence.
L3 survival probability.	Parameter	0·8522618 day^{-1}	Survival probability from[13] under the optimal temperature conditions as calculated from [19]. This is the maximum survival probability excluding density dependence.
L4 survival probability.	Parameter	0·7834648 day^{-1}	Survival probability from[13] under the optimal temperature conditions as calculated from [19]. This is the maximum survival probability excluding density dependence.
Pupae survival probability.	Parameter	0·6879704 day^{-1}	Survival probability from[13] under the optimal temperature conditions as calculated from [19]. This is the maximum survival probability excluding density dependence.
Egg hatch probability	Parameter	0·7479223 day^{-1}	Hatch probabilities from[13] under the optimal temperature conditions as calculated from [19].
L1 to L2 transition probability	Parameter	0·6201677 day^{-1}	Growth from [13] under the optimal temperature conditions as calculated from [19].
L2 to L3 transition probability	Parameter	0·4853487 day^{-1}	Growth from [13] under the optimal temperature conditions as calculated from [19].
L3 to L4 transition probability	Parameter	0·9302516 day^{-1}	Growth from [13] under the optimal temperature conditions as calculated from [19].
L4 to pupae transition probability	Parameter	0·6566482 day^{-1}	Growth from [13] under the optimal temperature conditions as calculated from [19].
Pupae to adult transition probability	Parameter	0·7973585 day^{-1}	Growth from [13] under the optimal temperature conditions as calculated from [19].
L1 resource usage	Parameter	0·2084118 day^{-1}	Size from [15] and scaled around L4.
L2 resource usage	Parameter	0·3179535 day^{-1}	Size from [15] and scaled around L4.
L3 resource usage	Parameter	0·5794099 day^{-1}	Size from [15] and scaled around L4.
L4 resource usage	Parameter	1 day^{-1}	Size from [15] and scaled around L4.
Pupae resource usage	Parameter	0 day^{-1}	Assumed to not impact density dependence, standard assumption of other models[11]
Probability host-seeking success human	Parameter	1 day^{-1}	Assume all host-seeking females will find a host.

Probability host-seeking success animal	Parameter	1 day^{-1}	Assume all host-seeking females will find a host.
Gonotrophic cycle length	Parameter	3 days	Standard assumed gonotrophic cycle length
Male sexual maturation probability	Parameter	1 day^{-1}	The rotation of Terminalia requires 24 hours[7].
Indoor feeding probability no ITN	Parameter	0·86	Estimate from[20] uses data pre the emergence of high levels of resistance.

Calibrating the Male Mosquito Survival Probabilities:

The measurement of insecticide survival for male mosquitoes is frequently not recorded, especially in experimental hut trials. But more simply their survival is also rarely recorded in standardised bioassays. Using data from[21] we fit a linear model between the paired survival of male and females with the same genetic background. Where $i_i^{♀FF}$ is the survival probability of female mosquitoes with the specified genotype to the insecticide, and therefore $i_i^{♂MM}$ is the survival probability of male mosquitoes with the corresponding genotype.

$$i_i^{♂MM} = \left(\beta_1 i_i^{♀FF}\right) + \beta_2$$

Equation S3.1

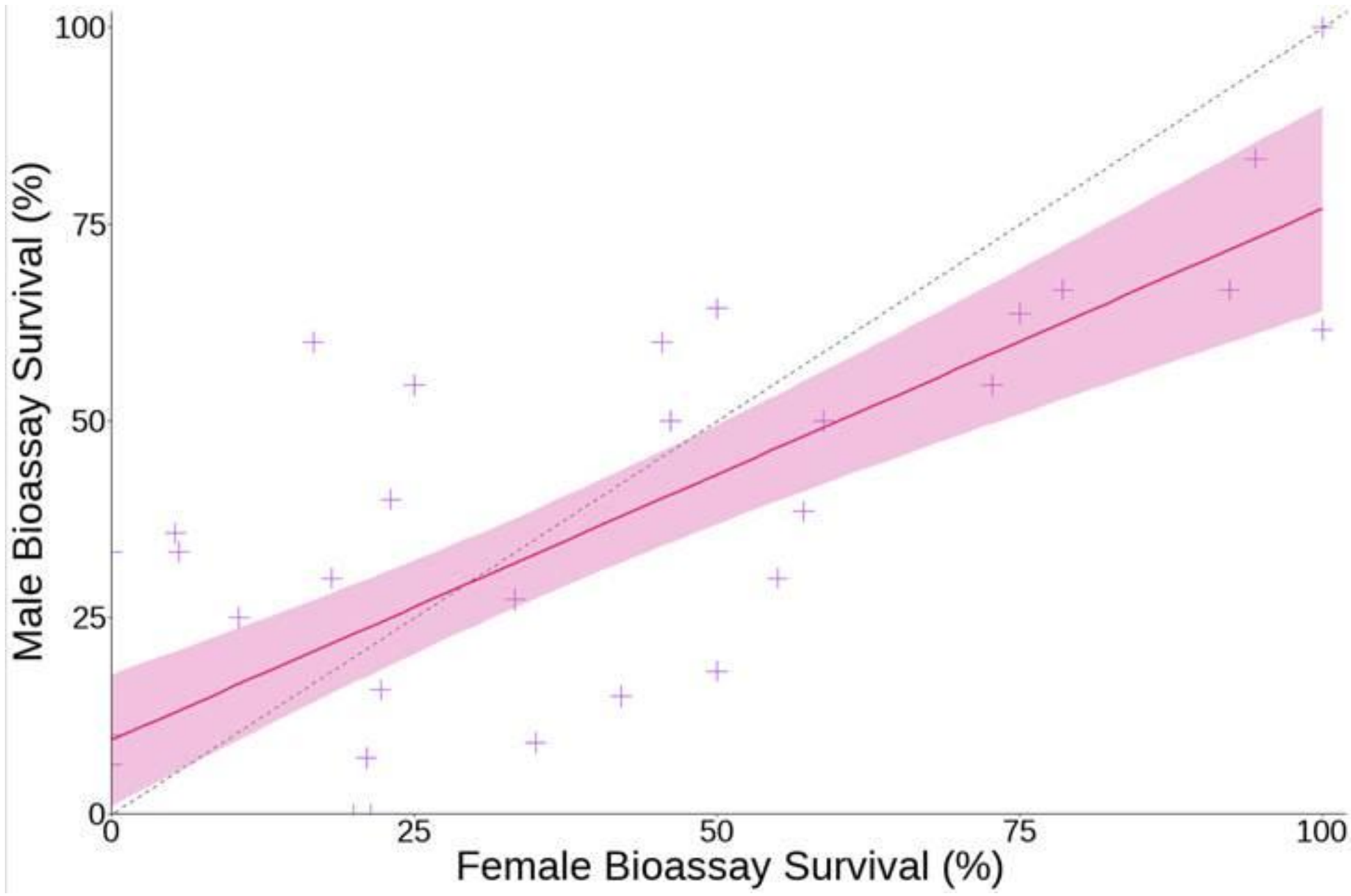

Fig I: Linear model of the relationship between female and male bioassay survival. The pink shaded area is the 95% confidence interval. The dashed line is the 1:1 ratio if there was no difference between female and male bioassay survival.

Table H: Output for the linear model between female and male bioassay survival.

Parameter	Estimate	Upper 95% CI	Lower 95% CI
β_1	0.67533	0.8543562	0.49630446
β_2	0.09425	0.1781747	0.01032074

Setting up Initial Simulation Conditions for Simulations:

The setting up of the simulations requires two steps. Step 1 is to identify a suitable carrying capacity value, which gives viable mosquito populations for most parameter set permutations. Step 2 involves obtaining the equilibrium point for the carrying given the parameter set permutations.

Step 1: Calibration of Carrying Capacity:

In the absence of climate module (Appendix 4), the model simulations use a fixed carrying capacity, where carrying capacity concerns the larval habitat. We define a viable mosquito population as being one where the equilibrium number of adult female mosquitoes (regardless of behavioural state) is between 10,000 and 1,000,000 when there are not vector control interventions deployed. These values were selected as being at the very extremes of mosquito population sizes. The calibration simulations included only the SS genotype. The calibration simulations were initialised with 10,000 female SS pupae and 10,000 male SS pupae. Simulations were run for 10 years where the model would reach steady state equilibrium.

Using a Latin hyperspace sampling approach from the lhs package [22], 5000 random parameter sets were generated (using values from Table 1 in the main manuscript). These were run with an initial a default carrying capacity of R_T being 10^6, 10^7, 10^8, 10^9 or 10^{10} (initial ad-hoc simulations indicated this was a suitable range of values to scan).

At the steady-state equilibrium (after 10 years), the total adult female population size was extracted (unmated and mated). Populations were defined as being viable (within the target population size range) or unviable (outside the target population size range). The carrying capacity which gave then highest percentage of viable populations was then identified, this was identified to be 10^7 giving viable populations in ~68% of the simulations. Further refined simulations using R_T values from $5\text{x}10^6$ to $3\text{x}10^7$ (with $5\text{x}10^6$ intervals) were run using a Latin hypercube of 5,000 parameter sets. From these simulations it was identified that the R_T value which gave the highest proportion of viable simulations was: $R_T = 2\text{x}10^7$, which gave viable populations in ~90% of populations and this value of R_T was used in the main simulations.

Step 2: Identify equilibrium point for carrying capacity and parameter values.

Having identified a suitable carrying capacity ($R_T = 2\text{x}10^7$), we use this carrying capacity over a larger number of random parameter sets. A sample of 100,000 parameter values were sampled (Table 1 including the parameter values insecticide resistance survival, ITN feeding probability and ITN coverage) using a Latin hyperspace sampling[22]. However, for these simulations the ITN coverage was then set to zero to switch off vector control. These 100,000 parameter sets were run for 10 years to generate the steady-state equilibrium values. The steady-state equilibrium values are therefore the population size values (and corresponding vectorial capacity) of the simulations in the absence of vector control.

Where population sizes were outside the viable population size, these were excluded and not replaced. The steady-state equilibrium values were then used to initiate the simulations when vector control (ITNs) was included (with or without resistance being able to evolve). For the simulations where resistance is absent (all individuals are SS), the initialization values are the values extracted at from the steady state equilibrium. Populations were categorised as viable or unviable. Viable populations were defied as having an adult female population size at steady-state in

the absence of interventions between 10,000 and 1,000,000. Unviable populations were not used in the simulations including interventions. Out of the 100,000 no intervention simulations run 90.73% gave viable populations.

From the steady state equilibrium, the number of: Eggs, L1 to L4 larval instars, Pupae, Adult males (immature, mature), Adult Females (unmated: Hostseeking, Resting, Oviposition) and Adult Females (mated: Hostseeking, Resting, Oviposition) were extracted. These values were distributed in Hardy-Weinberg equilibrium ($S^2 + 2RS + R^2 = 1$), where the initial R allele frequency was set to 10^{-6}, to allow for the inclusion of insecticide resistance. A similar process can be done for the two-gene simulations (Simulation Set 2), but instead calculates the initial genotype probabilities when two genes are present.

Appendix 4: Climate Module

4.1 Impact of Climatic Conditions:

The purpose of this section is to demonstrate how this coupled population dynamics and population genetics model can be extended to consider the impact of important environmental variables. We note that climatic conditions are play a key role of the regulation of mosquito populations. Increased rainfall will increase the carrying capacity of larval habits, leading to correspondingly larger larval and adult population sizes. Temperature will also play a role across multiple different life stages. Increased temperature gives faster larval growth rates (up to a point), and reduced temperatures give slower larval growth rates. Temperature also impacts the length of gonotrophic cycles. Of course, temperature will also impact the rate of evaporation (and therefore reducing the larval carrying capacity). Future extensions to the model will therefore need to include the impact of climatic conditions, however, for simplicity this functionality is not used in the presented simulations in the main manuscript. This is because the focus of this model was the inclusion of the dynamics of insecticide resistance genes in population dynamics models. Temperature would be expected to impact daily growth rates, such that $\delta_{L2(t)}^{♂MM}$ for example, would depend on temperature. Rainfall would impact R_T, which defines the total carrying capacity of the larval population. Temperature would also impact the adult mosquitoes. We only use the temperature aspect of the climatic module to parameterise our simulations, but this is included as a fixed daily temperature.

4.2 Carrying Capacity:

It is worth considering how the larval habitats of mosquitoes are likely to be affected by rainfall. This will likely also depend on the type of larval habit also. Larval habitats can be separated into two distinct groups. The first is permanent water bodies, which even during the dry season maintain a level of water to support mosquito populations. These will be impacted by rainfall by increasing the size of these permanent water bodies. There are then the temporary water bodies, such as puddles, which will only exist during the rainy season and are therefore entirely dependent on rainfall. The overall carrying capacity ($R_{T(t)}$) of all the larval habitats is therefore the sum of carrying capacities of both the temporary and permanent water bodies.

$$R_{T(t)} = r_1\left(\sum_{d=1}^{dmax} r_{f(t-d)}\right) + \left(r_2\left(\sum_{d=1}^{dmax} r_{f(t-d)}\right)R_P\right) + R_P$$

Equation S4.2.1

R_P is therefore the minimum carrying capacity of the larval habitats even after long periods without any rainfall as a result of permanent water bodies. r_1 is the factor which converts rainfall making small temporary into a carrying capacity. r_2 is the factor which converts rainfall impacting the size of permanent water bodies into carrying capacity. Larval habitats are generally categorised as permanent or temporary. By separating our larval habitat into two distinct groups (permanent and temporary), this will make any further extensions to larval source management more realistic, as it is generally only the "few, fixed and findable" (permanent) that are able to be realistically controlled. Note, in the presented simulations we consider only using a fixed carrying capacity, and the methodology presented here is to demonstrate the potential to incorporate a time-varying carrying capacity.

4.3 Impact of Temperature on Mosquito Biological Parameters

Temperature will impact the growth rates of mosquito in their egg, larval and pupal stages. We model the daily larval development rates and aquatic survival rates using thermal response curves as fit from [19]. These thermal curves were calculated based on laboratory data. For some biological parameters a Briere function is used, such that the response to temperature is unimodal:

$$d_r^T = c_d Temp_{(t)}\left(Temp_{(t)} - Temp_{crit}^{min}\right) * \left(\left(Temp_{crit}^{max} - Temp_{(t)}\right)^{\frac{1}{2}}\right)$$

Equation S4.3.1(a)

For other biological parameters a quadratic function is more biologically appropriate:

$$d_r^T = c_s\left(\left(c_q * (Temp_{(t)})\right) + \left(c_r * Temp_{(t)}\right)\right)$$

Equation S4.3.1(b)

The critical minimum temperature is the temperature at which the biological process can no longer be performed. The critical maximum temperature is the temperature above which the biological process can no longer be performed. Where c_c, c_s, c_q and c_r are modelled fitted constants[19], $T_{(t)}$ is the current temperature, and T_{crit}^{min} and T_{crit}^{max} are the critical minimum and maximum temperatures respectfully. We normalise this relationship such that:

$$D_r^T = \frac{d_r^T}{\max(d_r^T)}$$

Equation S4.3.2

This is done to allow different genotypes to have different baseline parameter values, but then allowing them to still be equally affected by temperature.

For larval development the Briere function was used, these modelled estimates were obtained from[19], such that c_d = 0.000111, T_{crit}^{min}= 14.7, T_{crit}^{max} = 34. For female mosquito egg laying batch size, this is modelled using the quadratic function, such that c_s = −97.7, c_q = −0.153, and c_r = 8.61. For egg, larvae and pupal survival, this is modelled using the quadratic function, again these modelled estimates we obtained from[19], such that c_s = −4.77, c_q = −0.00924, and c_r = 0.453. For adult daily survival, this is modelled using the quadratic function, such that c_s = 0.522, c_q = −0.000828, and c_r = 0.0367. The value of D_r^T is used to modulate the growth rates of the larval mosquitoes such that:

$$\delta_{L1(t)}^{♀FF} = g_{L1}^{♀FF} D_r^T$$

Equation S4.3.3(a♀)

$$\delta_{L2(t)}^{♀FF} = g_{L2}^{♀FF} D_r^T$$

Equation S4.3.3(b♀)

$$\delta_{L3(t)}^{♀FF} = g_{L3}^{♀FF} D_r^T$$

Equation S4.3.3(c♀)

$$\delta_{L4(t)}^{♀FF} = g_{L4}^{♀FF} D_r^T$$

Equation S4.3.3(d♀)

$$\delta_{P(t)}^{♀FF} = g_{P}^{♀FF} D_r^T$$

Equation S4.3.3(e♀)

$$\delta_{L1(t)}^{♂MM} = g_{L1}^{♂MM} D_r^T$$

Equation S4.3.3(a♂)

$$\delta_{L2(t)}^{♂MM} = g_{L2}^{♂MM} D_r^T$$

Equation S4.3.3(b♂)

$$\delta_{L3(t)}^{♂MM} = g_{L3}^{♂MM} D_r^T$$

Equation S4.3.3(c♂)

$$\delta_{L4(t)}^{♂MM} = g_{L4}^{♂MM} D_r^T$$

Equation S4.3.3(d♂)

$$\delta_{P(t)}^{♂MM} = g_{P}^{♂MM} D_r^T$$

Equation S4.3.3(e♂)

Where $g_{L4}^{♀FF}$ would be the maximum growth rate of the mosquito larvae. The value of $\delta_{L1(t)}^{♂MM}$ is returned the aquatic module equations.
The value of D_r^T is used to modulate the egg batch sizes:

$$\theta_{(t)}^{FF} = D_r^T \vartheta^{FF}$$

Equation S4.3.4

ϑ^{FF} is the average maximum number of eggs female mosquitoes with the corresponding genotype lay per gonotrophic cycle.
As this value gives probability of surviving from egg to adult, it must be converted to a daily survival probability.

$$\gamma_{L1(t)}^{♀FF} = \kappa_{L1}^{♀FF} D_r^T$$
Equation S4.3.5(a♀)

$$\gamma_{L2(t)}^{♀FF} = \kappa_{L2}^{♀FF} D_r^T$$
Equation S4.3.5(b♀)

$$\gamma_{L3(t)}^{♀FF} = \kappa_{L3}^{♀FF} D_r^T$$
Equation S4.3.5(c♀)

$$\gamma_{L4(t)}^{♀FF} = \kappa_{L4}^{♀FF} D_r^T$$
Equation S4.3.5(d♀)

$$\gamma_{P(t)}^{♀FF} = \kappa_{P}^{♀FF} D_r^T$$
`
Equation S4.3.5(e♀)

$$\gamma_{L1(t)}^{♂MM} = \kappa_{L1}^{♂MM} D_r^T$$
Equation S4.3.5(a♂)

$$\gamma_{L2(t)}^{♂MM} = \kappa_{L2}^{♂MM} D_r^T$$
Equation S4.3.5(b♂)

$$\gamma_{L3(t)}^{♂MM} = \kappa_{L3}^{♂MM} D_r^T$$
Equation S4.3.5(c♂)

$$\gamma_{L4(t)}^{♂MM} = \kappa_{L4}^{♂MM} D_r^T$$
Equation S4.3.5(d♂)

$$\gamma_{P(t)}^{♂MM} = \kappa_{P}^{♂MM} D_r^T$$
Equation S4.3.5(e♂)

Daily adult natural survival probability:

$$\rho_{H}^{♀FF} = \kappa_{H}^{♀FF} D_r^T$$
Equation S4.3.6(a♀)

$$\rho_{R}^{♀FF} = \kappa_{R}^{♀FF} D_r^T$$
Equation S4.3.6(b♀)

$$\rho_{O}^{♀FF} = \kappa_{O}^{♀FF} D_r^T$$
Equation S4.3.6(c♀)

$$\rho_{A}^{♂MM} = \kappa_{A}^{♂MM} D_r^T$$
Equation S4.3.6(d♂)

Again, it should be noted in the presented simulations, we assume a constant fixed temperature, but use the thermal curves to calibrate all parameter estimates to be implemented at the same temperature.

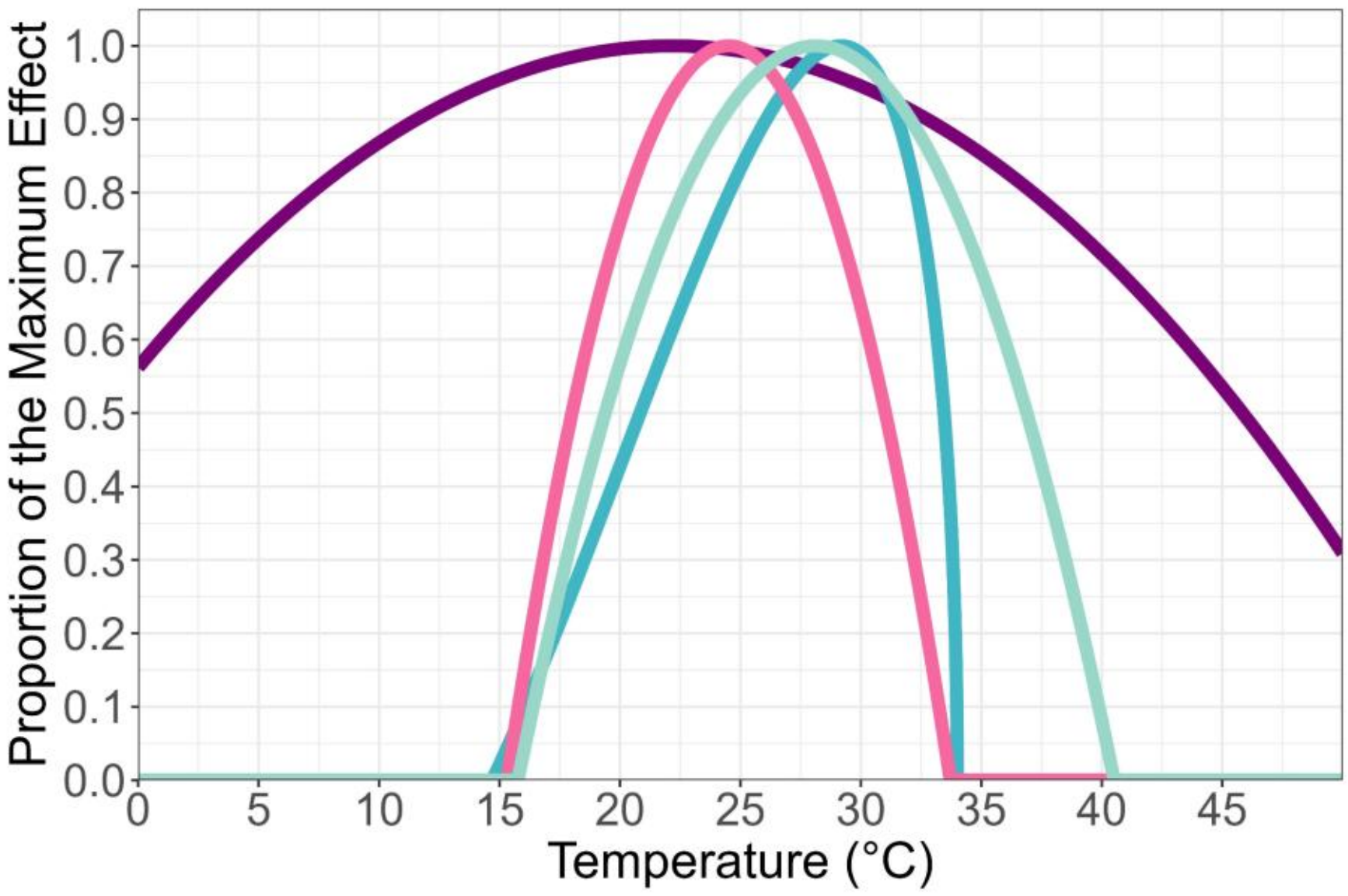

Fig J: Temperature Response Curves for Climate. Purple = adult daily survival probability; pink = larval daily survival probability; green = egg production; blue = larval growth rate. The proportion of the maximum effect value is then used to modify the probabilities of the respective parameter value.

Table I: Climate Module

Symbol	Description	Value	Rationale
r_1	A factor which converts rainfall which makes small temporary pools into the carrying capacity.	Not implemented	N/A
r_2	A factor which converts rainfall impacting the size of permanent water bodies into carrying capacity	Not implemented	N/A
$dmax$	The number of days of preceding rain which is used to calculate the carrying capacity.	Not implemented	N/A
$r_{f(t-d)}$	The amount of rainfall on day t minus d.	Not implemented	N/A
R_P	the minimum carrying capacity of the larval habitats even after long periods without any rainfall as a result of permanent water bodies	Not implemented	N/A
d_r^T	Thermal response modifier.	Internally calculated	See Equation S4.3.1
$Temp_{(t)}$	Temperature on day t.	Internal input.	N/A
c_d	Shape parameter of the Briere function.	For Larval development: 0·000111	From [19]
$Temp_{crit}^{min}$	Critical minimum temperature.	For Larval development: 14.7	From [19]
$Temp_{crit}^{max}$	Critical maximum temperature.	For Larval development: 34	From [19]
c_s	Constant of the quadratic thermal response curve.	For female mosquito egg laying batch size: -97·7<br><br>For egg, larvae and pupal survival: -4·77<br><br>For adult daily survival: 0·522	From [19]
c_q	Quadratic shape parameter of the quadratic thermal response curve.	For female mosquito egg laying batch size: -0·153	From [19]

		For egg, larvae and pupal survival: -0·00924<br><br>For adult daily survival: -0·000828	
c_r	Shape parameter of the quadratic thermal response curve.	For female mosquito egg laying batch size: 8·61<br><br>For egg, larvae and pupal survival: 0·453<br><br>For adult daily survival: 0·0367	From [19]
D_r^T	Normalised thermal response modifier	Internally calculated (0 - 1)	N/A
$g_{L1}^{♀FF}$	Maximum growth rate of female L1 larvae with the genotype FF.	0·6201677	From [13]
$g_{L2}^{♀FF}$	Maximum growth rate of female L2 larvae with the genotype FF.	0·4853487	From [13]
$g_{L3}^{♀FF}$	Maximum growth rate of female L3 larvae with the genotype FF.	0·9302516	From [13]
$g_{L4}^{♀FF}$	Maximum growth rate of female L4 larvae with the genotype FF.	0·6566482	From [13]
$g_{P}^{♀FF}$	Maximum development rate of female pupae with the genotype FF.	0·7973585	From [13]
$g_{L1}^{♂MM}$	Maximum growth rate of male L1 larvae with the genotype MM.	0·6201677	From [13]
$g_{L2}^{♂MM}$	Maximum growth rate of male L2 larvae with the genotype MM.	0·4853487	From [13]
$g_{L3}^{♂MM}$	Maximum growth rate of male L3 larvae with the genotype MM.	0·9302516	From [13]
$g_{L4}^{♂MM}$	Maximum growth rate of male L4 larvae with the genotype MM.	0·6566482	From [13]
$g_{P}^{♂MM}$	Maximum development rate of male pupae with the genotype MM.	0·7973585	From [13]
ϑ^{FF}	Maximum number of eggs produced by female mosquitoes of the genotype FF.	187	From [18] with modification to the maximum temperature through [19]
$\kappa_{L1}^{♀FF}$	Maximum daily survival rate of female L1 larvae with the genotype FF.	0·9888291	From [13]
$\kappa_{L2}^{♀FF}$	Maximum daily survival rate of female L2 larvae with the genotype FF.	0·9005225	From [13]
$\kappa_{L3}^{♀FF}$	Maximum daily survival rate of female L3 larvae with the genotype FF.	0·8522618	From [13]
$\kappa_{L4}^{♀FF}$	Maximum daily survival rate of female L2 larvae with the genotype FF.	0·7834648	From [13]
$\kappa_{P}^{♀FF}$	Maximum daily survival rate of female pupae with the genotype FF.	0·6879704	From [13]
$\kappa_{L1}^{♂MM}$	Maximum daily survival rate of male L1 larvae with the genotype MM.	0·9888291	From [13]
$\kappa_{L2}^{♂MM}$	Maximum daily survival rate of male L2 larvae with the genotype MM.	0·9005225	From [13]

$\kappa_{L3}^{♂MM}$	Maximum daily survival rate of male L2 larvae with the genotype MM.	0·8522618	From [13]
$\kappa_{L4}^{♂MM}$	Maximum daily survival rate of male L2 larvae with the genotype MM.	0·7834648	From [13]
$\kappa_{P}^{♂MM}$	Maximum daily survival rate of male pupae with the genotype MM.	0·6879704	From [13]
$\kappa_{H}^{♀FF}$	Daily natural survival probability while in the hostseeking state.	0·73	From [23]
$\kappa_{R}^{♀FF}$	Daily natural survival probability while in the resting state.	0·73	From [23]
$\kappa_{O}^{♀FF}$	Daily natural survival probability while in the oviposition state.	0·73	From [23]
$\kappa_{A}^{♂MM}$	Daily natural survival probability of adult mosquitoes	0·73	From [23]

Appendix 5: Extension to a two and three gene system:

We further demonstrate the extension of PopGEM modelling framework to account for multiple genes (up to a maximum of three). This therefore means that resistance to three separate insecticides can be tracked, and/or the role of three separate genes for the resistance to a single insecticide can also be explored (as is the case with polygenic resistance). By including 3 genes in the model, this leads to 27 possible genotypes, and therefore 729 mated genotypes. This inevitably increases model computational complexity. As a result, we do not include linkage, as including genetic linkage increases the computation resources required by the model, which is already high due to the number of genotypes, stages and states being tracked. In the presented simulations in the main manuscript (e.g., Simulation Set 2), we only use the two gene extension.

In this model extension we further consider that insecticides can be deployed as larvicides (not explored), insecticide treated nets (ITNs) or indoor residual spraying (IRS) (again not explored); which may all be used as constituent components of any IRM strategy. The ITNs, IRS and larvicides can each be deployed as mixture formulations. Note, a maximum of three different insecticides can be deployed in any one simulation, therefore for example if a mixture ITN was deployed only a single-insecticide formulation IRS or larvicide could be additionally deployed.

While we present the mathematical equations for the three gene model, these equations can easily be modified to a two gene model by replacing any instance of *FF1FF2FF3* with *FF1FF2*, and likewise *MM1MM2MM3* with *MM1MM2*. The model code is separately maintained for the two and three gene models, as running simulations with three genes (when only two are needed) unnecessarily increases the computational requirements. The descriptions of the equations are largely similar to those in the single gene version, except now track all three genes.

We do not use the IRS and larvicide capability in the presented manuscript, nevertheless we believe it is important to demonstrate the technical extension of the PopGEM model to be able to incorporate alternative vector control tools. We also do not use the three gene model, our reasoning for this is the need to focus the current discussion on the evaluation of next-generation mixture ITNs which is the most immediately practical IRM tool available to national malaria control programs.

5.1 Update to the Oviposition Module

The total number of female eggs laid by all ovipositing females with the mated genotype *FF1FF2FF3_MM1MM2MM3* is:

$$F_{E(t)}^{♀FF1FF2FF3\_MM1MM2MM3} = A_{O(t)}^{♀FF1FF2FF3\_MM1MM2MM3} \theta_{(t)}^{FF1FF2FF3} \Phi^{FF1FF2FF3} a_o^{♀FF1FF2FF3}$$

Equation S5.1.1(♀)

And the equivalent for male eggs:

$$F_{E(t)}^{♂FF1FF2FF3\_MM1MM2MM3} = A_{O(t)}^{♀FF1FF2FF3\_MM1MM2MM3} \theta_{(t)}^{FF1FF2FF3} (1-\Phi^{FF1FF2FF3}) a_o^{♀FF1FF2FF3}$$

Equation S5.1.1(♂)

The number of female and male eggs laid in total for each of the 27 genotypes is therefore:

$$N_{E(t)}^{♀FF1FF2FF3} = \sum \left(F_{E(t)}^{♀FF1FF2FF\_MM1MM2MM3} * E_{FF1FF2FF3}^{FF1FF2FF3\_MM1MM2MM3}\right)$$

Equation S5.1.2(♀)

$$N_{E(t)}^{♂MM1MM2MM3} = \sum \left(F_{E(t)}^{♀FF1FF2FF\_MM1MM2MM3} * E_{FF1FF2FF3}^{FF1FF2FF3\_MM1MM2MM3}\right)$$

Equation S5.1.2(♂)

Where $E_{FF1FF2FF3}^{FF1FF2FF3\_MM1MM2MM3}$ is the proportion of eggs which have the genotype *FF1FF2FF3* given they are laid by females with the mated genotype of *FF1FF2FF3_MM1MM2MM3* (Fig K for the two gene version, and Figure Fig L for the three gene version):

$$E_{FF1FF2FF3}^{FF1FF2FF3\_MM1MM2MM3} = G_{FF1}^{FF1MM1} * G_{FF2}^{FF2MM2} * G_{FF3}^{FF3MM3}$$

Equation S5.1.3(♀)

$$E_{MM1MM2MM3}^{FF1FF2FF3\_MM1MM2MM3} = G_{MM1}^{FF1MM1} * G_{MM2}^{FF2MM2} * G_{MM3}^{FF3MM3}$$

Equation S5.1.3(♂)

The values of G_{FF1}^{FF1MM1}, G_{FF2}^{FF2MM2}, G_{FF3}^{FF3MM3}, G_{MM1}^{FF1MM1}, G_{MM2}^{FF2MM2}, G_{MM3}^{FF3MM3} are calculated following classical Mendelian inheritance.

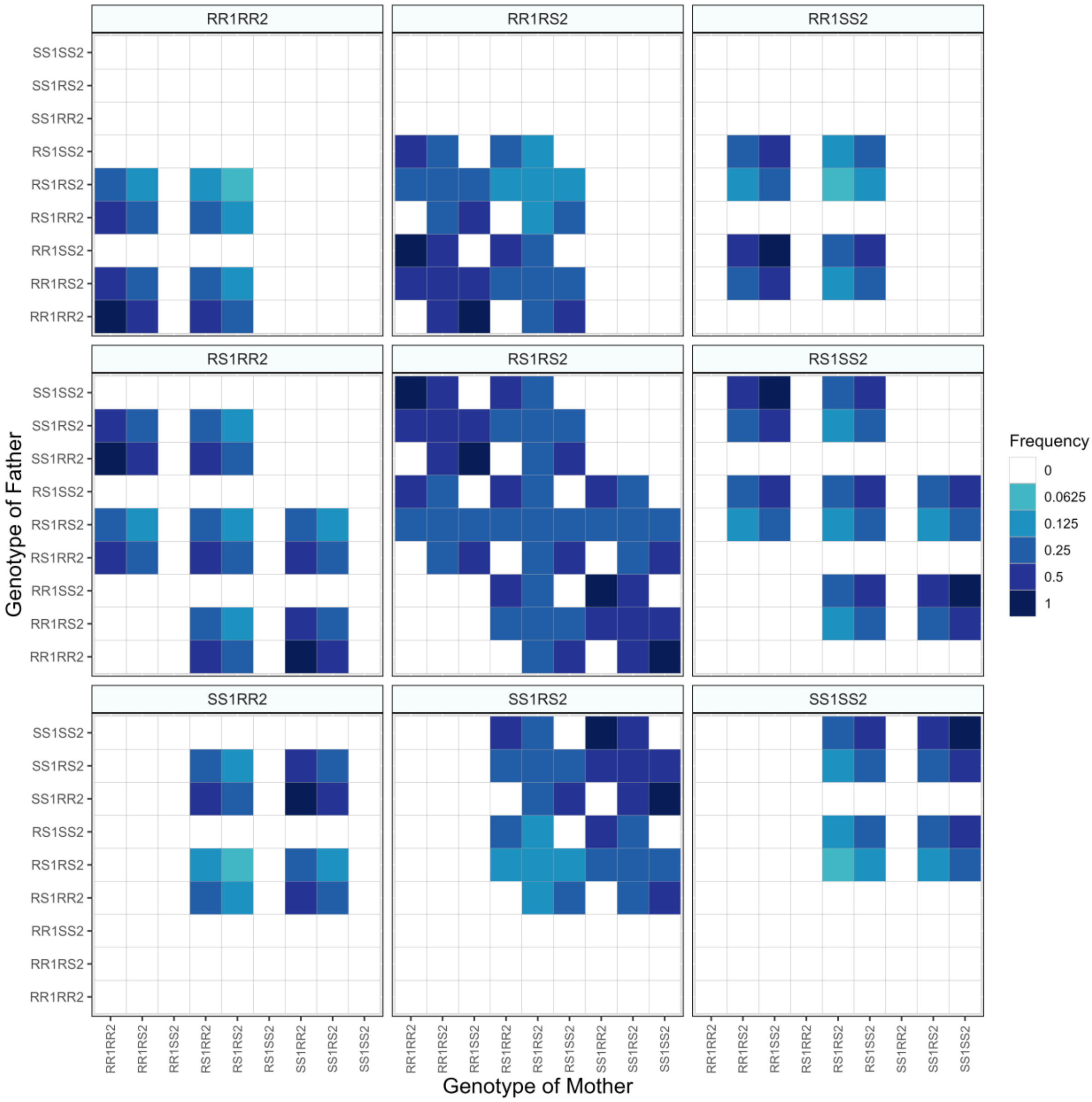

Fig K: Egg Genotype given Parental Genotypes for Two Genes. The title of each panel is the genotype of the eggs laid. The x axis is the genotype of the mother, and the y axis is the genotype of the father. The colour fill indicates the proportion of the eggs of the corresponding mating that produce the genotype of the specified egg as detailed in the panel title. Where the darker the blue colour the higher the proportion of the eggs. Egg genotypes not produced by a specific mating pair are filled in white.

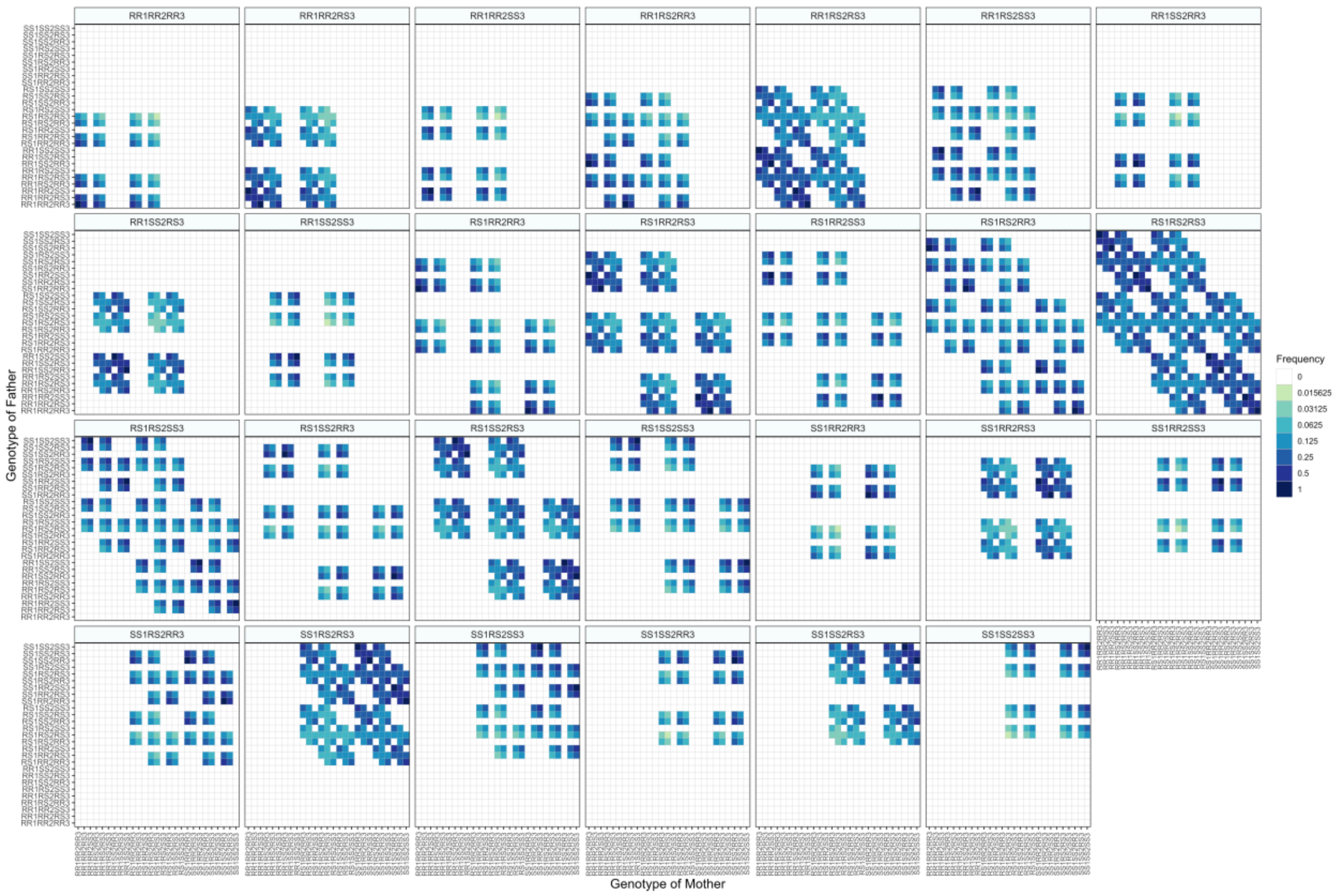

Fig L: Egg Genotype given Parental Genotypes for Three Genes. The title of each panel is the genotype of the eggs laid. The x axis is the genotype of the mother, and the y axis is the genotype of the father. The colour fill indicates the proportion of the eggs of the corresponding mating that produce the genotype of the specified egg as detailed in the panel title. Where the darker the blue colour the higher the proportion of the eggs. Egg genotypes not produced by a specific mating pair are filled in white.

5.2 Update to the Aquatic Module

Next, we update the aquatic module. We also consider that larvicides can be deployed. Which have coverage ($Y_{larvicide}$) of the total larval habitats.

The probability a female egg remains as a female egg to the next day (does not die or hatch) is:

$$K_{E(t)}^{♀FF1FF2FF3} = (\rho_{E(t)}^{♀FF1FF2FF3}\ (1 - h_{E(t)}^{♀FF1FF2FF3})\ i_{larvicide:1}^{♀FF1FF2FF3}\ i_{larvicide:2}^{♀FF1FF2FF3}\ Y_{larvicide}) + \ (\rho_{E(t)}^{♀FF1FF2FF3}\ (1 - h_{E(t)}^{FF1FF2FF3})\ (1 - Y_{larvicide}))$$

Equation S5.2.1(♀)

And for male eggs:

$$K_{E(t)}^{♂MM1MM2MM3} = (\rho_{E(t)}^{♂MM1MM2MM3}\ (1 - h_{E(t)}^{♂MM1MM2MM3})\ i_{larvicide:1}^{♂MM1MM2MM3} i_{larvicide:2}^{♂MM1MM2MM3}\ Y_{larvicide}) + \ (\rho_{E(t)}^{♂MM1MM2MM3}\ (1 - h_{E(t)}^{♂MM1MM2MM3})\ (1 - Y_{larvicide}))$$

Equation S5.2.1(♂)

Where $\rho_{E(t)}^{♀FF1FF2FF3}$ and $\rho_{E(t)}^{♂MM1MM2MM3}$ are the natural survival probability of eggs (which can be temperature dependent, see Climate Module, Appendix 4), $h_{E(t)}^{♀FF1FF2FF3}$ and $h_{E(t)}^{♂MM1MM2MM3}$ are the hatch probability (can be temperature dependent, see Climate Module, Appendix 4). $Y_{larvicide}$ is the coverage of the larvicide, defined as the proportion of the larval habitats treated. The values $i_{larvicide:1}^{♀FF1FF2FF3}$, $i_{larvicide:2}^{♀FF1FF2FF3}$ and $i_{larvicide:1}^{♂MM1MM2MM3}$, $i_{larvicide:2}^{♂MM1MM2MM3}$ are the survival probability to the larvicides given the genotypes. Note, if no larvicide is deployed these values would all be set to 1. If only a single-ingredient formulation is used then $i_{larvicide:2}^{♀FF1FF2FF3}$ and $i_{larvicide:2}^{♂MM1MM2MM3}$ are set to 1. Note, as not all larvicides are ovicidal, if the larvicide is not ovicidal then $i_{larvicide:1}^{♀FF1FF2FF3}$, $i_{larvicide:2}^{♀FF1FF2FF3}$ and $i_{larvicide:1}^{♂MM1MM2MM3}$, $i_{larvicide:2}^{♂MM1MM2MM3}$ set to 1 for all genotypes when targeting eggs.

The daily probability a female egg given the genotype FF1FF2FF3 transitions from egg to L1 is calculated as:

$$T_{E(t)}^{\female FF1FF2FF3} = \left(\rho_{E(t)}^{\female FF1FF2FF3}\ h_{E(t)}^{\female FF1FF2FF3}\ i_{larvicide:1}^{\female FF1FF2FF3} i_{larvicide:2}^{\female FF1FF2FF3}\ Y_{larvicide}\right) + (\rho_{E(t)}^{\female FF1FF2FF3}\ h_{E(t)}^{\female FF1FF2FF3}\ (1 - Y_{larvicide}))$$

Equation S5.2.2(♀)

The daily probability a male egg given the genotype MM1MM2MM3 transitions from egg to L1 is calculated as:

$$T_{E(t)}^{\male MM1MM2MM3} = \left(\rho_{E(t)}^{\male MM1MM2MM3}\ h_{E(t)}^{\male MM1MM2MM3}\ i_{larvicide:1}^{\male MM1MM2MM3} i_{larvicide:2}^{\male MM1MM2MM3}\ Y_{larvicide}\right) + (\rho_{E(t)}^{\male MM1MM2MM3}\ h_{E(t)}^{\male MM1MM2MM3}\ (1 - Y_{larvicide}))$$

Equation S5.2.2(♂)

The daily probability L1 larvae remain as L1 larvae for female and males given the genotypes is:

$$K_{L1(t)}^{\female FF1FF2FF3} = ((1 - \delta_{L1(t)}^{\female FF1FF2FF3})\ \rho_{L1(t)}^{\female FF1FF2FF3}\ i_{larvicide:1}^{\female FF1FF2FF3} i_{larvicide:2}^{\female FF1FF2FF3}\ Y_{larvicide}) + ((1 - \delta_{L1(t)}^{\female FF1FF2FF3}) * \rho_{L1(t)}^{\female FF1FF2FF3}\ (1 - Y_{larvicide}))$$

Equation S5.2.3(♀)

$$K_{L1(t)}^{\male MM1MM2MM3} = ((1 - \delta_{L1(t)}^{\male MM1MM2MM3})\ \rho_{L1(t)}^{\male MM1MM2MM3}\ i_{larvicide:1}^{\male MM1MM2MM3} i_{larvicide:2}^{\male MM1MM2MM3}\ Y_{larvicide}) + ((1 - \delta_{L1(t)}^{\male MM1MM2MM3}) * \rho_{L1(t)}^{\male MM1MM2MM3}\ (1 - Y_{larvicide}))$$

Equation S5.2.3(♂)

The daily probability L1 larvae transition to L2 larvae for female and males given the genotypes is:

$$T_{L1(t)}^{\female FF1FF2FF3} = (\rho_{L1(t)}^{\female FF1FF2FF3} \delta_{L1(t)}^{\female FF1FF2FF3}\ i_{larvicide:1}^{\female FF1FF2FF3} i_{larvicide:2}^{\female FF1FF2FF3} Y_{larvicide}) + (\rho_{L1(t)}^{\female FF1FF2FF3}\ \delta_{L1(t)}^{\female FF1FF2FF3}(1 - Y_{larvicide}))$$

Equation S5.2.4(♀)

$$T_{L1(t)}^{\male MM1MM2MM3} = (\rho_{L1(t)}^{\male MM1MM2MM3} \delta_{L1(t)}^{\male MM1MM2MM3} i_{larvicide:1}^{\male MM1MM2MM3} i_{larvicide:2}^{\male MM1MM2MM3} Y_{larvicide}) + (\rho_{L1(t)}^{\male MM1MM2MM3}\ \delta_{L1(t)}^{\male MM1MM2MM3}(1 - Y_{larvicide}))$$

Equation S5.2.4(♂)

The daily probability L2 larvae remain as L2 larvae for female and males given the genotypes is:

$$K_{L2(t)}^{\female FF1FF2FF3} = ((1 - \delta_{L2(t)}^{\female FF1FF2FF3})\ \rho_{L2(t)}^{\female FF1FF2FF3}\ i_{larvicide:1}^{\female FF1FF2FF3} i_{larvicide:2}^{\female FF1FF2FF3}\ Y_{larvicide}) + ((1 - \delta_{L2(t)}^{\female FF1FF2FF3}) * \rho_{L2(t)}^{\female FF1FF2FF3}\ (1 - Y_{larvicide}))$$

Equation S5.2.5(♀)

$$K_{L2(t)}^{\male MM1MM2MM3} = ((1 - \delta_{L2(t)}^{\male MM1MM2MM3})\ \rho_{L2(t)}^{\male MM1MM2MM3}\ i_{larvicide:1}^{\male MM1MM2MM3} i_{larvicide:2}^{\male MM1MM2MM3}\ Y_{larvicide}) + ((1 - \delta_{L2(t)}^{\male MM1MM2MM3}) * \rho_{L2(t)}^{\male MM1MM2MM3}\ (1 - Y_{larvicide}))$$

Equation S5.2.5(♂)

The daily probability L2 larvae transition to L3 larvae for female and males given the genotypes is:

$$T_{L2(t)}^{\female FF1FF2FF3} = (\rho_{L2(t)}^{\female FF1FF2FF3} \delta_{L2(t)}^{\female FF1FF2FF3}\ i_{larvicide:1}^{\female FF1FF2FF3} i_{larvicide:2}^{\female FF1FF2FF3} Y_{larvicide}) + (\rho_{L2(t)}^{\female FF1FF2FF3}\ \delta_{L2(t)}^{\female FF1FF2FF3}(1 - Y_{larvicide}))$$

Equation S5.2.6(♀)

$$T_{L2(t)}^{\male MM1MM2MM3} = (\rho_{L2(t)}^{\male MM1MM2MM3} \delta_{L2(t)}^{\male MM1MM2MM3} i_{larvicide:1}^{\male MM1MM2MM3} i_{larvicide:2}^{\male MM1MM2MM3} Y_{larvicide}) + (\rho_{L2(t)}^{\male MM1MM2MM3}\ \delta_{L2(t)}^{\male MM1MM2MM3}(1 - Y_{larvicide}))$$

Equation S5.2.6(♂)

The daily probability L3 larvae remain as L3 larvae for female and males given the genotypes is:

$$K_{L3(t)}^{♀FF1FF2FF3} = ((1 - \delta_{L3(t)}^{♀FF1FF2FF3})\, \rho_{L3(t)}^{♀FF1FF2FF3}\, i_{larvicide:1}^{♀FF1FF2FF3} i_{larvicide:2}^{♀FF1FF2FF3}\, Y_{larvicide}) + ((1 - \delta_{L3(t)}^{♀FF1FF2FF3}) * \rho_{L3(t)}^{♀FF1FF2FF3}\, (1 - Y_{larvicide}))$$

Equation S5.2.7(♀)

$$K_{L3(t)}^{♂MM1MM2MM3} = ((1 - \delta_{L3(t)}^{♂MM1MM2MM3})\, \rho_{L3(t)}^{♂MM1MM2MM3}\, i_{larvicide:1}^{♂MM1MM2MM3} i_{larvicide:2}^{♂MM1MM2MM3}\, Y_{larvicide}) + ((1 - \delta_{L3(t)}^{♂MM1MM2MM3}) * \rho_{L3(t)}^{♂MM1MM2MM3}\, (1 - Y_{larvicide}))$$

Equation S5.2.7(♂)

The daily probability L3 larvae transition to L4 larvae for female and males given the genotypes is:

$$T_{L3(t)}^{♀FF1FF2FF3} = (\rho_{L3(t)}^{♀FF1FF2FF3} \delta_{L3(t)}^{♀FF1FF2FF3} i_{larvicide:1}^{♀FF1FF2FF3} i_{larvicide:2}^{♀FF1FF2FF3} Y_{larvicide}) + (\rho_{L3(t)}^{♀FF1FF2FF3}\, \delta_{L3(t)}^{♀FF1FF2FF3} (1 - Y_{larvicide}))$$

Equation S5.2.8(♀)

$$T_{L3(t)}^{♂MM1MM2MM3} = (\rho_{L3(t)}^{♂MM1MM2MM3} \delta_{L3(t)}^{♂MM1MM2MM3} i_{larvicide:1}^{♂MM1MM2MM3} i_{larvicide:2}^{♂MM1MM2MM3} Y_{larvicide}) + (\rho_{L3(t)}^{♂MM1MM2MM3}\, \delta_{L3(t)}^{♂MM1MM2MM3} (1 - Y_{larvicide}))$$

Equation S5.2.8(♂)

The daily probability L4 larvae remain as L4 larvae for female and males given the genotypes is:

$$K_{L4(t)}^{♀FF1FF2FF3} = ((1 - \delta_{L4(t)}^{♀FF1FF2FF3})\, \rho_{L4(t)}^{♀FF1FF2FF3}\, i_{larvicide:1}^{♀FF1FF2FF3} i_{larvicide:2}^{♀FF1FF2FF3}\, Y_{larvicide}) + ((1 - \delta_{L4(t)}^{♀FF1FF2FF3}) * \rho_{L4(t)}^{♀FF1FF2FF3}\, (1 - Y_{larvicide}))$$

Equation S5.2.9(♀)

$$K_{L4(t)}^{♂MM1MM2MM3} = ((1 - \delta_{L4(t)}^{♂MM1MM2MM3})\, \rho_{L4(t)}^{♂MM1MM2MM3}\, i_{larvicide:1}^{♂MM1MM2MM3} i_{larvicide:2}^{♂MM1MM2MM3}\, Y_{larvicide}) + ((1 - \delta_{L4(t)}^{♂MM1MM2MM3}) * \rho_{L4(t)}^{♂MM1MM2MM3}\, (1 - Y_{larvicide}))$$

Equation S5.2.9(♂)

The daily probability L4 larvae transition to pupae for female and males given the genotypes is:

$$T_{L4(t)}^{♀FF1FF2FF3} = (\rho_{L4(t)}^{♀FF1FF2FF3} \delta_{L4(t)}^{♀FF1FF2FF3} i_{larvicide:1}^{♀FF1FF2FF3} i_{larvicide:2}^{♀FF1FF2FF3} Y_{larvicide}) + (\rho_{L4(t)}^{♀FF1FF2FF3}\, \delta_{L4(t)}^{♀FF1FF2FF3} (1 - Y_{larvicide}))$$

Equation S5.2.10(♀)

$$T_{L4(t)}^{♂MM1MM2MM3} = (\rho_{L4(t)}^{♂MM1MM2MM3} \delta_{L4(t)}^{♂MM1MM2MM3} i_{larvicide:1}^{♂MM1MM2MM3} i_{larvicide:2}^{♂MM1MM2MM3} Y_{larvicide}) + (\rho_{L4(t)}^{♂MM1MM2MM3}\, \delta_{L4(t)}^{♂MM1MM2MM3} (1 - Y_{larvicide}))$$

Equation S5.2.10(♂)

The daily probability pupae remain as pupae for female and males given the genotypes is:

$$K_{P(t)}^{♀FF1FF2FF3} = ((1 - \delta_{P(t)}^{♀FF1FF2FF3})\, \rho_{P(t)}^{♀FF1FF2FF3}\, i_{larvicide:1}^{♀FF1FF2FF3} i_{larvicide:2}^{♀FF1FF2FF3}\, Y_{larvicide}) + ((1 - \delta_{P(t)}^{♀FF1FF2FF3}) * \rho_{P(t)}^{♀FF1FF2FF3}\, (1 - Y_{larvicide}))$$

Equation S5.2.11(♀)

$$K_{P(t)}^{♂MM1MM2MM3} = ((1 - \delta_{P(t)}^{♂MM1MM2MM3})\, \rho_{P(t)}^{♂MM1MM2MM3}\, i_{larvicide:1}^{♂MM1MM2MM3} i_{larvicide:2}^{♂MM1MM2MM3}\, Y_{larvicide}) + ((1 - \delta_{P(t)}^{♂MM1MM2MM3}) * \rho_{P(t)}^{♂MM1MM2MM3}\, (1 - Y_{larvicide}))$$

Equation S5.2.11(♂)

Note, not all larvicides are ovicidal or pupacidal. In these situations, the $i_{larvicide}^{♀FF1FF2FF3}$ for the particular stage (egg or pupae) can be set at 1 such that the larvicide does not apply additional mortality to these stages. This is because many larvicides require ingestion by the larvae to become toxic.
The density dependent population regulation equations require extending to include all the possible genotype permutations. For resource usage by each larval instar state:

$$R_{L1(t)} = \alpha_{L1}\left(\sum L1_{(t-1)}^{♀FF1FF2FF3} + \sum L1_{(t-1)}^{♂MM1MM2MM3}\right)$$

Equation S5.2.12 (a)

$$R_{L1(t)} = \alpha_{L2}\left(\sum L2_{(t-1)}^{♀FF1FF2FF3} + \sum L2_{(t-1)}^{♂MM1MM2MM3}\right)$$

Equation S5.2.12 (b)

$$R_{L1(t)} = \alpha_{L3}\left(\sum L3_{(t-1)}^{♀FF1FF2FF3} + \sum L3_{(t-1)}^{♂MM1MM2MM3}\right)$$

Equation S5.2.12(c)

$$R_{L4(t)} = \alpha_{L4}\left(\sum L4_{(t-1)}^{♀FF1FF2FF3} + \sum L1_{(t-1)}^{♂MM1MM2MM3}\right)$$

Equation S5.2.12(d)

$$R_{P(t)} = \alpha_{P}\left(\sum P_{(t-1)}^{♀FF1FF2FF3} + \sum P_{(t-1)}^{♂MM1MM2MM3}\right)$$

Equation S5.2.12(e)

These values of total resource usage by each aquatic stage are then passed to equation S1.2.8(f) to subsequently calculate DDPR as previously detailed in equation S1.2.9 in Appendix 1.

5.3 Update to the Adult Male Module

The updated adult male module further considers how male mosquitoes may encounter ITNs and IRS if and when they enter houses.

The number of immature (denoted by the subscript I) adult male mosquitoes is calculated as previously in equation S1.3(a), but now tracking with the three genes:

$$A_{I(t)}^{♂MM1MM2MM3} = \left(A_{I(t-1)}^{♂MM1MM2MM3} K_{I(t)}^{♂MM1MM2MM3}\right) + \left(P_{(t-1)}^{♂MM1MM2MM3} T_{P(t)}^{♂MM1MM2MM3}\right)$$

Equation S5.3.1(♂)

Where $K_{I(t)}^{♂MM1MM2MM3}$ describes the daily probability that a a sexually immature male with genotype MM1MM2MM3 remains as a sexually immature male. And $T_{P(t)}^{♂MM1MM2MM3}$ describes the emergence probability of the pupae which emerge as sexually immature males. The calculation of

$$T_{P(t)}^{♂MM1MM2MM3} = (\rho_{P(t)}^{♂MM1MM2MM3} \delta_{P(t)}^{♂F♂MM1MM2MM3} i_{larvicide:1}^{♂MM1MM2MM3} i_{larvicide:2}^{♂MM1MM2MM3} Y_{larvicide}) + (\rho_{P(t)}^{♂MM1MM2MM3} \delta_{P(t)}^{♂MM1MM2MM3} (1 - Y_{larvicide}))$$

Equation S5.3.2(♂)

Note, if the larvicide is non-pupacidal, then $i_{larvicide}^{♂FF1FF2FF3}$ is set to 1 for the pupae.

Next, we must account for the ways in which adult male mosquitoes may encounter insecticides deployed as ITNs and IRS. This first depends on the innate daily house entry probability of male mosquitoes ($endophily_{♂}$), the proportion of houses treated with ITNs (Y_{ITN}), IRS (Y_{IRS}) or both ITNs and IRS ($Y_{ITN+IRS}$).

The daily probability an adult male mosquito with genotype MM1MM2MM3 encounters and survives contact with an ITN (either singe insecticide, or mixture) is:

$$ITN_{♂}^{MM1MM2MM3} = (endophily_{♂}\, \varepsilon_{ITN|ITN}^{♂}\, Y_{ITN}\, i_{ITN:1}^{♂MM1MM2MM3} i_{ITN:2}^{♂MM1MM2MM3}) + (endophily_{♂}\ Y_{ITN+IRS}\ \varepsilon_{ITN|ITN+IRS}^{♂}\, i_{ITN:1}^{♂MM1MM2MM3} i_{ITN:2}^{♂MM1MM2MM3})$$

Equation S5.3.3(♂)

The parameter $\varepsilon_{ITN|ITN}^{♂}$ is the probability that a male mosquito encounters the ITN considering it went inside a house with an ITN. The parameter $\varepsilon_{ITN|ITN+IRS}^{♂}$ is the probability that a male mosquito encounters the ITN considering it went inside a house with both an ITN and IRS. Because different insecticides have different repellent properties and that there is spatial separation between the ITN and IRS, it is important to consider the implication that even when both are present, they both may not be contacted.

The daily probability an adult male mosquito with genotype MM1MM2MM3 encounters and survives contact with an IRS (either singe insecticide, or mixture) is:

$$IRS_{♂}^{MM1MM2MM3} = (endophily_{♂}\ \varepsilon_{IRS|IRS}^{♂}\ Y_{IRS}\ i_{IRS:1}^{♂MM1MM2MM3} i_{IRS:2}^{♂MM1MM2MM3}) + (endophily_{♂}\ Y_{IRS}\ \varepsilon_{IRS|ITN+IRS}^{♂} i_{IRS:1}^{♂MM1MM2MM3} i_{IRS:2}^{♂MM1MM2MM3})$$

Equation S5.3.4(♂)

The daily probability an adult male mosquito with genotype MM1MM2MM3 encounters and survives contact with both an ITN and IRS (either singe insecticide, or mixture) is:

$$ITN\_IRS_{♂}^{MM1MM2MM3} = (endophily_{♂}\ \varepsilon_{ITN+IRS|ITN+IRS}^{♂}\ Y_{ITN+IRS} i_{IRS:1}^{♂MM1MM2MM3} i_{IRS:2}^{♂MM1MM2MM3} i_{ITN:1}^{♂MM1MM2MM3} i_{ITN:2}^{♂MM1MM2MM3})$$

Equation S5.3.5(♂)

The daily probability an adult male mosquito with genotype MM1MM2MM3 does not encounter insecticides is:

$$Avoids_{♂}^{MM1MM2MM3} = (1 - endophily_{♂}) + (endophily_{♂} \left(1 - (Y_{ITN} + Y_{IRS} + Y_{ITN+IRS})\right)) + (endophily_{♂}(1 - \varepsilon_{ITN|ITN}^{♂})\, Y_{ITN}) + (endophily_{♂}\ (1 - \varepsilon_{IRS|IRS}^{♂})\ Y_{IRS}) + (endophily_{♂} * Y_{ITN+IRS} * (1 - (\varepsilon_{ITN|ITN+IRS}^{♂} + \varepsilon_{IRS|ITN+IRS}^{♂} + \varepsilon_{ITN+IRS|ITN+IRS}^{♂})))$$

Equation S5.3.6(♂)

The daily probability a sexually immature adult male mosquito with genotype MM1MM2MM3 remains as a sexually immature adult male mosquito is:

$$K_{I(t)}^{♂MM1MM2MM3} = (Avoids_{♂}^{MM1MM2MM3} + ITN_{♂}^{MM1MM2MM3} + IRS_{♂}^{MM1MM2MM3} + ITN\_IRS_{♂}^{MM1MM2MM3}) * (\rho_{A}^{♂MM1MM2MM3}) * (1 - \delta_{A}^{♂MM1MM2MM3})$$

Equation S5.3.7(♂)

The daily probability a sexually immature adult male mosquito with genotype MM1MM2MM3 transitions to become a sexually mature adult male mosquito is:

$$T_{M(t)}^{♂MM1MM2MM3} = (Avoids_{♂}^{MM1MM2MM3} + ITN_{♂}^{MM1MM2MM3} + IRS_{♂}^{MM1MM2MM3} + ITN\_IRS_{♂}^{MM1MM2MM3}) * (\rho_{A}^{♂MM1MM2MM3}) * \delta_{A}^{♂MM1MM2MM3}$$

Equation S5.3.8(♂)

The daily probability a sexually mature adult male mosquito with genotype MM1MM2MM3 survives and remains as a sexually mature adult male mosquito is:

$$K_{M(t)}^{♂MM1MM2MM3} = (Avoids_{♂}^{MM1MM2MM3} + ITN_{♂}^{MM1MM2MM3} + IRS_{♂}^{MM1MM2MM3} + ITN\_IRS_{♂}^{MM1MM2MM3}) * (\rho_{A}^{♂MM1MM2MM3})$$

Equation S5.3.9(♂)

The number of sexually mature (denoted by the M subscript) male mosquitoes with genotype MM1MM2MM3 on a given day is therefore the number of sexually mature males which remain as so, plus the number of sexually immature males which survive and become sexually mature.

$$A_{M(t)}^{♂MM1MM2MM3} = \left(A_{M(t-1)}^{♂MM1MM2MM3} K_{M(t)}^{♂MM1MM2MM3}\right) + \left(A_{I(t-1)}^{♂MM1MM2MM£} T_{M(t)}^{♂MM1MM2MM3}\right)$$
Equation S5.3.10(♂)

5.4 Update to the Unmated Female Model

The number of newly emerging virgin (denoted by the v subscript) adult female mosquitoes with genotype FF1FF2FF3 is therefore the number of newly emerged females from pupae:

$$A_{v(t)}^{♀FF1FF2FF3} = P_{(t-1)}^{♀FF1FF2FF3} T_{P(t)}^{♀FF1FF2FF3}$$
Equation S5.4.1(♀)

The daily probability of emergence is therefore:

$$T_{P(t)}^{♀FF1FF2FF3} = (\rho_{P(t)}^{♀FF1FF2FF3} \delta_{P(t)}^{♀FF1FF2FF3} i_{larvicide}^{♀FF1FF2FF3} Y_{larvicide}) + (\rho_{P(t)}^{♀FF1FF2FF3} \delta_{P(t)}^{♀FF1FF2FF3} (1 - Y_{larvicide}))$$
Equation S5.4.2(♀)

Which considers the impact of density dependent survival (see Section 1.2.2. in Appendix 1), larvicidal interventions (as detailed in section 5.2 of Appendix 5) and temperature dependent survival rates (see climate module, Appendix 4).

The number of virgin host seeking females of genotype FF1FF2FF3 is therefore the number of virgin females from the previous day which survived having still yet mated, and the number of newly emerged females which have yet to mate:

$$A_{Hv\,(t)}^{FF1FF2FF3} = K_{H(t)}^{♀FF1FF2FF3} \left((1-(sQ))A_{v(t)}^{FF1FF2FF3}\right) + \left((1-s)A_{Hv(t-1)}^{FF1FF2FF3}\right)$$
Equation S5.4.3(♀)

The number of blood-fed and resting (denoted by the subscript R) virgin females of genotype FF1FF2FF3 is therefore the number of host-seeking females which remain unmated having now successfully blood-fed and survived, or having previously blood-fed and remain digesting the bloodmeal:

$$A_{Rv\,(t)}^{FF1FF2FF3} = (1-s)\left(\left(T_{H(t)}^{♀FF1FF2FF3} A_{Hv(t-1)}^{FF1FF2FF3}\right) + \left(K_{R(t)}^{♀FF1FF2FF3} A_{R(t-1)}^{FF1FF2FF3}\right)\right)$$
Equation S5.4.4(♀)

The number of virgin adult females in the oviposition state (denoted by the subscript O) are therefore the number of as yet unmated resting females who transition to the oviposition state, or those who were already in the oviposition state and remain.

$$A_{Ov\,(t)}^{FF1FF2FF3} = (1-s)\left(\left(T_{R(t)}^{♀FF1FF2FF3} A_{Rv(t-1)}^{FF1FF2FF3}\right) + \left(K_{Ov(t)}^{♀FF1FF2FF3FF} A_{Ov(t-1)}^{FF1FF2FF3}\right)\right)$$
Equation S5.4.5(♀)

Importantly, virgin females in the oviposition state must mate before they can lay eggs and transition back to the host-seeking state to start the next gonotrophic cycle. For simplicity female mating/swarming success (s) is the same for all genotypes, but we do note that this could be a fitness cost associated with insecticide resistance. When looking at the importance of this parameter, the single gene model can, in the first instance, be used to assess the importance of this parameter.

The daily probability a virgin host-seeking female mosquito with the genotype FF1FF2FF3 remains in their current state is:

$$K_{H(t)}^{♀FF1FF2FF3} = \rho_{H(t)}^{♀F1FF2FF3}\Bigg(\Big(H\varepsilon_H^{♀}ZY_{ITN}(1 - f_{HI|net}^{♀FF1FF2FF3})\, i_{ITN:1}^{♀FF1FF2FF3} i_{ITN:2}^{♀FF1FF2FF3}\Big) + \Big(H\varepsilon_H^{♀}Z(1 - Y_{ITN})(1 - f_{HI}^{♀FF1FF2FF3})\Big) + \Big(H\varepsilon_H^{♀}(1 - Z)(1 - f_{HO}^{♀})\Big) + \Big((1 - H)\varepsilon_A^{♀}(1 - f_A^{♀})\Big) + \Big(1 - \Big(\Big((1 - H)\varepsilon_A^{♀}\Big) + \Big(H\varepsilon_H^{♀}\Big)\Big)\Big)\Bigg)$$

Equation S5.4.6(♀)

The value $f_{HI|net}^{♀FF1FF2FF3}$ is the probability of successfully blood-feeding when the human host is using an ITN (single insecticide or mixture) and is dependent on the genotype of the mosquito. $i_{ITN:1}^{♀FF1FF2FF3}$ and $i_{ITN:2}^{♀FF1FF2FF3}$ are the insecticide survival probabilities to the insecticides used in the ITN, of which there may be one or two insecticides.

The daily probability a virgin host-seeking female mosquito with the genotype FF1FF2FF3 transitions to the resting state is:

$$T_{H(t)}^{♀FF1FF2FF3} = \rho_{H(t)}^{♀F1FF2FF3}\Big(\Big(H\varepsilon_H^{♀}ZY_{ITN} f_{HI|net}^{♀FF1FF2FF3}\, i_{ITN:1}^{♀FF1FF2FF3} i_{ITN:2}^{♀FF1FF2FF3}\Big) + \Big(H\varepsilon_H^{♀}Z(1 - Y_{ITN})f_{HI}^{♀}\Big) + \Big(H\varepsilon_H^{♀FF}(1 - Z)f_{HO}^{♀}\Big) + \Big((1 - H)\varepsilon_A^{♀} f_A^{♀F}\Big)\Big)$$

Equation S5.4.7(♀)

The daily probability a virgin resting female mosquito with the genotype FF1FF2FF3 remains in the resting state is:

$$K_{R(t)}^{♀FF1FF2FF3} = \Big(\Big(\rho_R^{♀FF1FF2FF3}\Big(1 - \delta_R^{♀}\Big)\Big) * (1 - ((Y_{ITN} + Y_{ITN+IRS})endophily_{♀})\Big) + \Big(\Big(\rho_R^{♀FF1FF2FF3}\Big(1 - \delta_R^{♀}\Big)\Big)((Y_{ITN} + Y_{ITN+IRS})endophily_{♀})\, i_{IRS:1}^{♀FF1FF2FF3} i_{IRS:2}^{♀FF1FF2FF3}\Big)$$

Equation S5.4.8(♀)

This includes the capability to include insecticides deployed as an IRS, which may be solo formulations or mixtures. The value $endophily_{♀}$ is the daily indoor resting probability. The values $i_{IRS:1}^{♀FF1FF2FF3}$ and $i_{IRS:2}^{♀FF1FF2FF3}$ and the survival probabilities to the insecticides deployed as IRS and depend on the resistance genotypes.

The daily probability a virgin resting female mosquito with the genotype FF1FF2FF3 transitions to the resting state is:

$$T_{R(t)}^{♀FF1FF2FF3} = \Big(\Big(\rho_{Rv(t)}^{♀FF1FF2FF3}\delta_{R(t)}^{♀FF1FF2FF3}\Big) * (1 - ((Y_{ITN} + Y_{ITN+IRS})endophily_{♀})\Big) + \Big(\Big(\rho_{Rv(t)}^{♀FF1FF2FF3}\delta_{R(t)}^{♀FF1FF2FF3}\Big)((Y_{ITN} + Y_{ITN+IRS})endophily_{♀})\, i_{IRS:1}^{♀FF1FF2FF3} i_{IRS:2}^{♀FF1FF2FF3}\Big)$$

Equation S5.4.9(♀)

The daily probability a virgin female mosquito in the oviposition with the genotype FF1FF2FF3 remains in the oviposition state is:

$$K_{Ov(t)}^{♀FF1FF2FF3} = \rho_{O(t)}^{♀FF1FF2FF3}$$

Equation S5.4.10(♀)

Note, the mating probability is included in equation S5.3.4(♀).

5.5 Update to the Mating Model
We further update the mating model to include all genotypes in the three gene model.

The value $w^{♂}_{MM1MM2MM3}$ is the individual mating competitiveness of male mosquitoes with the genotype MM1MM2MM3. This is then weighted by the number of male mosquitoes with the specified genotype:

$$C^{♂}_{MM1MM2MM3(t)} = A^{♂MM1MM2MM3}_{M(t)} w^{♂}_{MM1MM2MM3}$$

Equation S5.5.1(♂)

The proportion of all matings which males of the genotype MM1MM2MM3 is therefore their own population size weighted competitiveness, divided the sum for all genotypes:

$$M^{♂}_{MM1MM2MM3(t)} = \frac{C^{♂}_{MM1MM2MM3(t)}}{\sum C^{♂}_{MM1MM2MM3(t)}}$$

Equation S5.5.2(♂)

The number of newly mated female host-seeking females with the genotype FF1FF2FF3 who mate with males with the genotype MM1MM2MM3 is therefore:

$$A^{♀FF1FF2FF3\_MM1MM2MM3}_{H_{new(t)}} = M^{♂}_{MM1MM2MM3(t)} s \left(\left(A^{♀FF1FF2FF3}_{V(t)} Q \right) + A^{♀FF1FF2FF3}_{Hv(t-1)} \right)$$

Equation S5.5.3(♀)

The number of newly mated female resting females with the genotype FF1FF2FF3 who mate with males with the genotype MM1MM2MM3 is therefore:

$$A^{♀FF1FF2FF3\_MM1MM2MM3}_{R\_new(t)} = M^{♂}_{MM1MM2MM3(t)} s A^{♀FF1FF2FF3}_{Rv(t-1)}$$

Equation S5.5.4(♀)

The number of newly mated female host-seeking females with the genotype FF1FF2FF3 who mate with males with the genotype MM1MM2MM3 is therefore:

$$A^{♀FF1FF2FF3\_MM1MM2MM3}_{O\_new(t)} = M^{♂}_{MM1MM2MM3(t)} s A^{♀FF1FF2FF3}_{Ov(t-1)}$$

Equation S5.5.5(♀)

The newly mated $A^{♀FF1FF2FF3\_MM1MM2MM3}_{H_{new(t)}}$, $A^{♀FF1FF2FF3\_MM1MM2MM3}_{R\_new(t)}$ and $A^{♀FF1FF2FF3\_MM1MM2MM3}_{O\_new(t)}$ female mosquitoes now enter the mated female module.

5.6 Update to the Mated Female Module

The number of mated female host-seeking (denoted by the H subscript) with the genotype FF1FF2FF3 who mated with males with the genotype MM1MM2MM3 are:

$$\begin{aligned} A^{♀FF1FF2FF3\_MM1MM2MM3}_{H(t)} &= \left(K^{♀FF1FF2FF3}_{H(t)} \left(A^{♀FF1FF2FF3\_MM1MM2MM3}_{H(t-1)} + A^{♀FF1FF2FF3\_MM1MM2MM3}_{H\_new(t)} \right) \right) \\ &+ \left(T^{♀FF1FF2FF3}_{O(t)} \left(A^{♀FF1FF2FF3\_MM1MM2MM3}_{O(t)} + A^{♀FF1FF2FF3\_MM1MM2MM3}_{O\_new(t)} \right) \right) \end{aligned}$$

Equation S5.6.1(♀)

Which is the number remaining as host-seeking, and the number which successfully completed oviposition and can now start their next gonotrophic cycle. Note, $K^{♀FF1FF2FF3}_{H(t)}$, $T^{♀FF1FF2FF3}_{H(t)}$, $K^{♀FF1FF2FF3}_{R(t)}$ and $K^{♀FF1FF2FF3}_{R(t)}$ are calculated in Equation S5.4.6(♀), Equation S5.4.7(♀), Equation S5.4.8(♀) and Equation S5.4.9(♀) respectively.

The daily probability of a mated ovipositing female remaining in the oviposition state is:

$$K^{♀FFFF1FF2FF3}_{O(t)} = \left(\rho^{♀FF1FF2FF3}_{O(t)} \left(1 - a^{♀}_{O} \right) \right)$$

Equation S5.6.2(♀)

The daily probability of a mated ovipositing female transiting to the host-seeking state to start her next gonotrophic cycle is:

$$T_{O(t)}^{♀FF1FF2FF3} = \rho_{O(t)}^{♀FF1FF2FF3}\, a_O^{♀}$$

Equation S5.6.3(♀)

Where $a_O^{♀}$ is the daily probability of successful oviposition.

The number of mated female mosquitoes in the resting state is therefore the number of resting females which remain in the resting state, and those host-seeking mosquitoes which successfully blood-fed.

$$A_{R(t)}^{♀FF1FF2FF3\_MM1MM2MM3} = \left(K_{R(t)}^{♀FF1FF2FF3}\left(A_{R(t-1)}^{♀FF1FF2FF3\_MM1MM2MM3} + A_{R\_new(t)}^{♀FF1FF2FF3\_MM1MM2MM3}\right)\right) + \left(\left(A_{H(t-1)}^{♀FF1FF2FF3\_MM1MM2MM3} + A_{H\_new(t)}^{♀FF1FF2FF3\_MM1MM2MM3}\right) T_{H(t)}^{♀FF1FF2FF3}\right)$$

Equation S5.6.4(♀)

The number of mated female mosquitoes in the oviposition state is therefore the number of resting females which transition to the oviposition state, and those ovipositing females which remain in the ovipositing state having failed to lay eggs:

$$A_{O(t)}^{♀FF1FF2FF3\_MM1MM2MM3} = \left(T_{R}^{♀FF1FF2FF3}\left(A_{R(t-1)}^{♀FF1FF2FF3\_MM1MM2MM3} + A_{R\_new(t)}^{♀FF1FF2FF3\_MM1MM2MM3}\right)\right) + \left(\left(A_{O(t-1)}^{♀FF1FF2FF3\_MM1MM2MM3} + A_{O\_new(t)}^{♀FF1FF2FF3\_MM1MM2MM3}\right) K_{O(t)}^{♀FF1FF2FF3}\right)$$

Equation S5.6.5(♀)

5.7 Defining insecticide survival in the two and three gene model

Resistance to each insecticide is able to be encoded for by each of the genes, therefore allowing cross resistance. Cross resistance is frequently highlighted as a key concern for insecticide resistance management, and its inclusion in mathematical models has been shown to be important[24,25] (albeit often lacking[26]). Many models have made claims regarding how cross resistance will impact IRM, without either explicitly including a methodology for cross resistance in the model or failing to adequately detail how cross resistance is included in models. We can therefore consider each gene can impact the resistance phenotype to any insecticide. Here we detail how cross resistance for a three gene model can be included in the model. We consider that each gene is able to provide some resistance phenotype (survival) to each insecticide, and that these genes work multiplicatively.

The survival probability to each insecticide is therefore:

$$i_i = 1 - \left((1 - genotype_{1i}) * (1 - genotype_{2i}) * (1 - genotype_{3i})\right)$$

Equation S5.7.1

$$i_j = 1 - \left(\left(1 - genotype_{1j}\right) * \left(1 - genotype_{2j}\right) * (1 - genotype_{3j})\right)$$

Equation S5.7.2

$$i_j = 1 - \left((1 - genotype_{1k}) * (1 - genotype_{2k}) * (1 - genotype_{3k})\right)$$

Equation S5.7.3

Where i_i is the survival probability to insecticide i. $genotype_{1i}$ is the survival probability to insecticide i as a result of the genotype of gene 1, $genotype_{2i}$ is the survival probability to insecticide i as a result of the genotype of gene 2 and $genotype_{3i}$ is the survival probability to insecticide i as a result of the genotype of gene 3. Note, for a two gene simulation, the $genotype_3$ portion of the equation is removed. Where the genotype will be one of SS, RS or RR. Examples (using arbitrary values) are given in Fig M (two gene) and Fig N (three gene) to help communicate the cross resistance survival calculation.

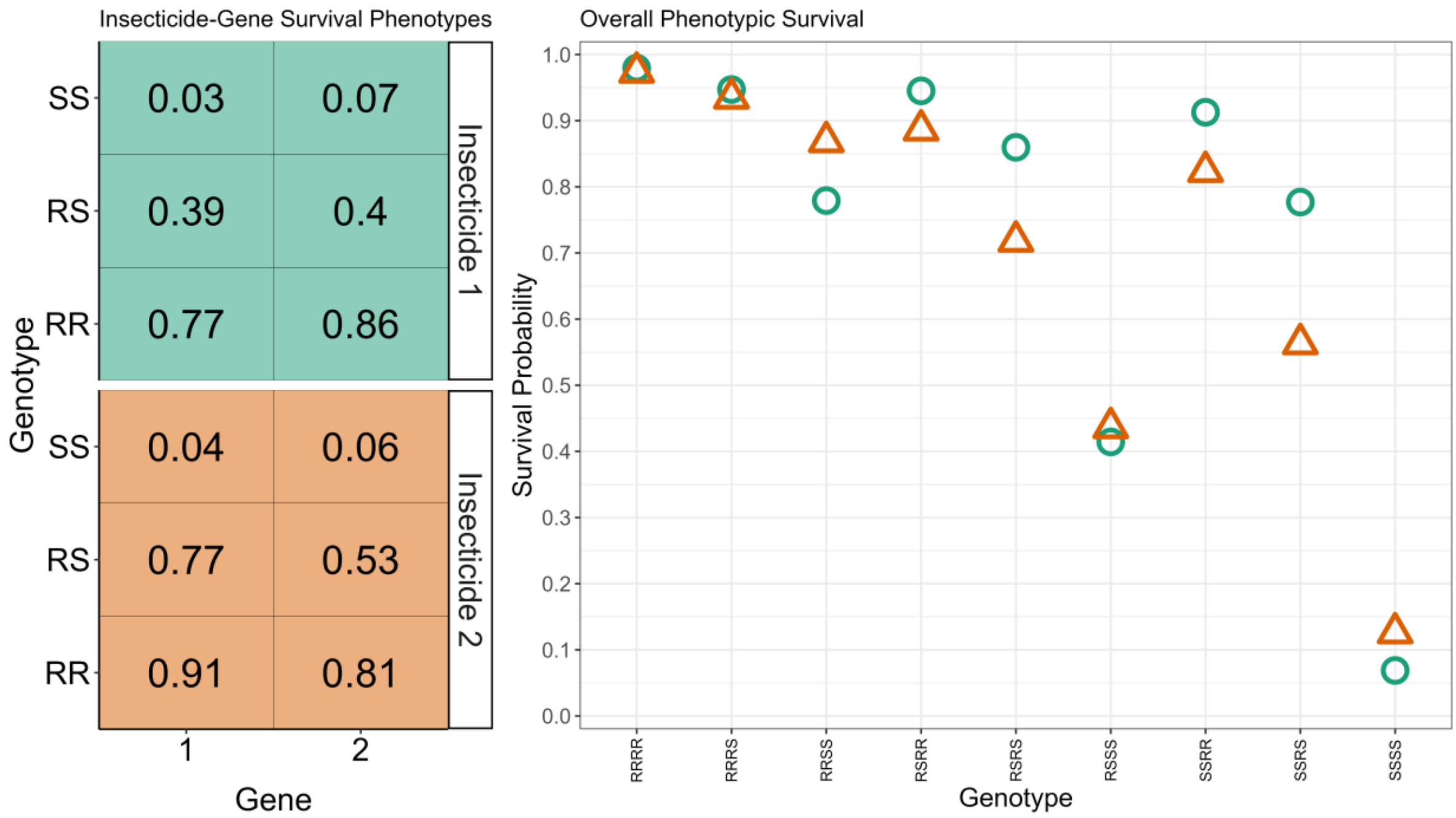

Fig M: Example survival probability across susceptible–resistant genotypes of the two gene model under two insecticide treatments. This plot contains an example of the survival probabilities for a set of two genes to two insecticides. The left coloured panels indicate the survival probability to an insecticide (specified on the right y-axis label), each gene (x axis) and corresponding genotype (y-axis) has. The scatterplot then combines these individual level survivals using equations S5.7.1 to S5.7.2. The survival to each insecticide (as noted by the colour) is then mapped for all three genes. Note, this is an example plot only for illustrative purposes, and the phenotypic values were generated randomly. Note, cross resistance was not explored in the presented simulations.

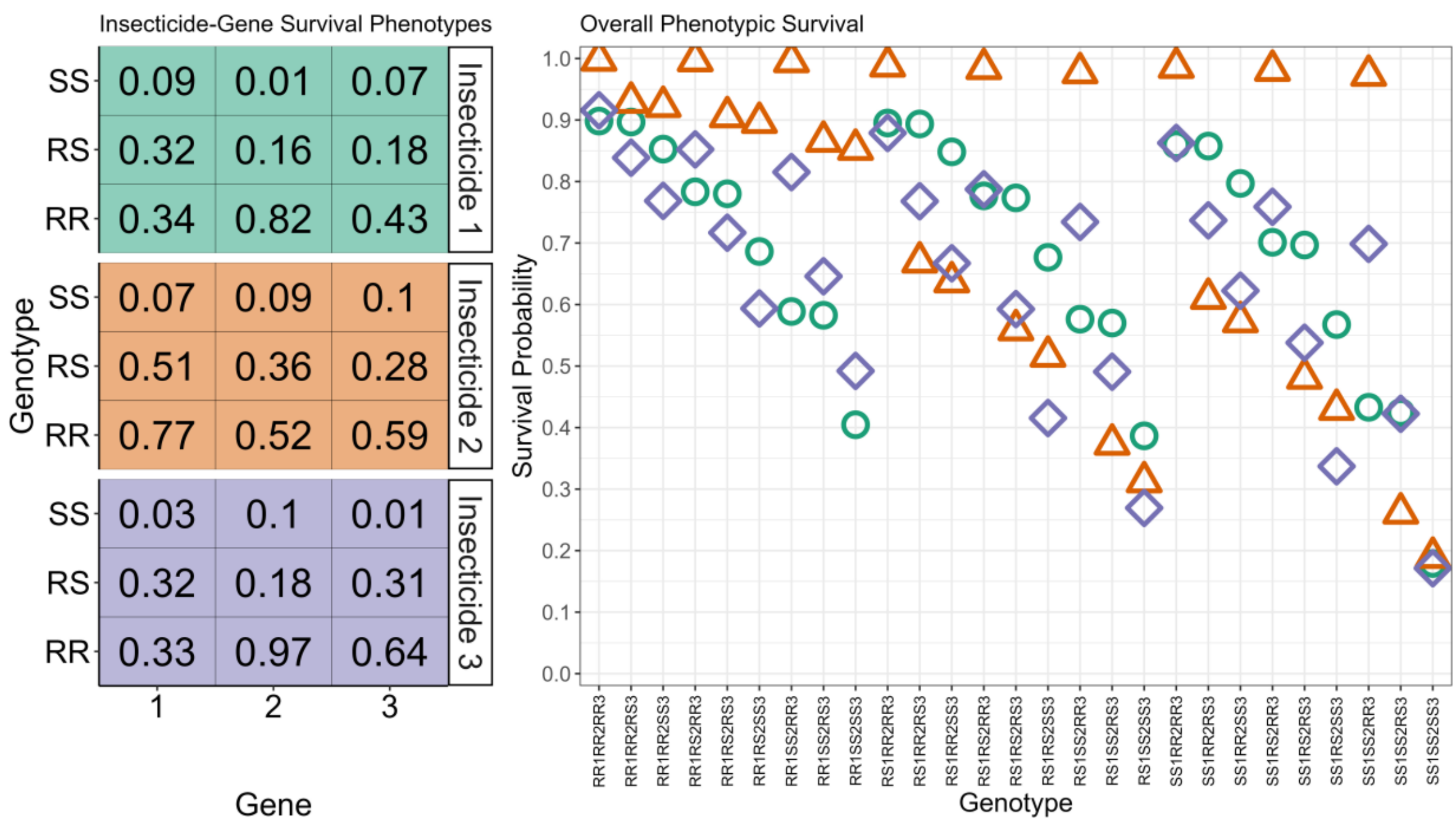

Fig N: Example survival probability across susceptible–resistant genotypes of the three gene model under three insecticide treatments. This plot contains an example of the survival probabilities for a set of three genes to three insecticides. The left coloured panels indicate the survival probability to an insecticide (specified on the right y-axis label), each gene (x axis) and corresponding genotype (y-axis) has. The scatterplot then combines these individual level survivals using equations S5.7.1 to S5.7.3. The survival to each insecticide (as noted by the

colour) is then mapped for all three genes. Note, this is an example plot only for illustrative purposes, and the phenotypic values were generated randomly. Note, cross resistance was not explored in the presented simulations.

Defining fitness costs associated with resistance in the two and three gene model
We must also account for fitness costs associated with insecticide resistance.
Therefore, using a fully susceptible individual (SS1SS2, two gene or SS1SS2SS3, three gene) as the baseline, we can describe the fitness in relation to these individuals.
For the two gene model:

$$fitness_b = genotype_{1b} * genotype_{2b}$$

Equation S5.7.1

For the three gene model:

$$fitness_b = genotype_{1b} * genotype_{2b} * genotype_{3b}$$

Equation S5.7.2

Where SS genes would be expected to have no impact on fitness cost. Therefore, SS1SS2SS3 would have a relative fitness of 1. Fitness costs for any particular life history trait can be removed by setting the genotype fitnesses all to 1. Where $fitness_b$ is the relative fitness of the overall genotype for the biological parameter b. $genotype_{1b} * genotype_{2b} * genotype_{3b}$ are the relative fitness of genotypes for the biological parameter b, and can be specified uniquely for the biological parameters of: Egg batch sizes, Larval growth, Larval survival, Male mating fitness, Natural adult survival (male and female). Examples of the implementation of this for a two gene (Fig O) and three gene (Fig P) system are presented using random values. It must be noted; fitness costs were not explored in the simulations presented in the main manuscript.

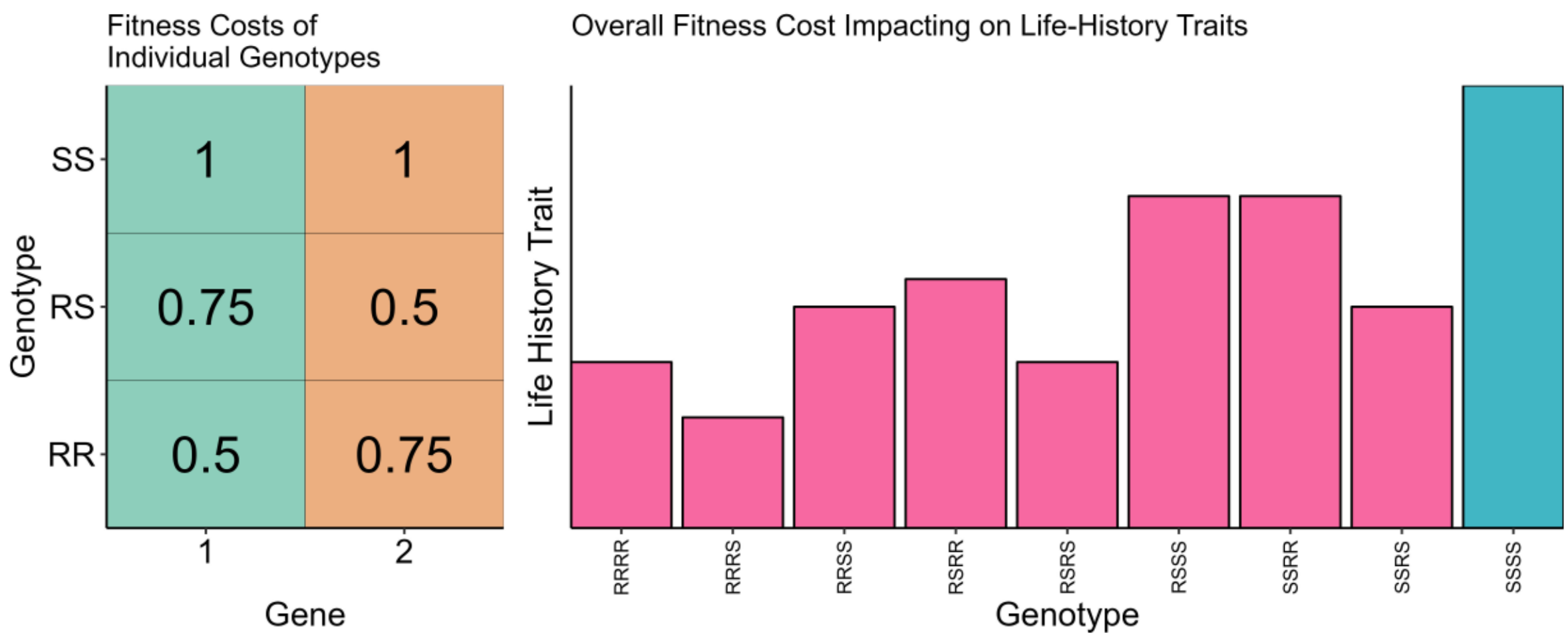

Fig O: Overall fitness cost in two gene model genotypes on life-history traits. The left-hand plot indicates the fitness costs associated with specific genes, with the values indicating the proportion of the fitness which is retained. These are then combined (detailed in equation S5.7.1), to give the overall fitness of the whole genotype for a specific life-history trait. Note, the values given in this plot are used only for illustrative purposes.

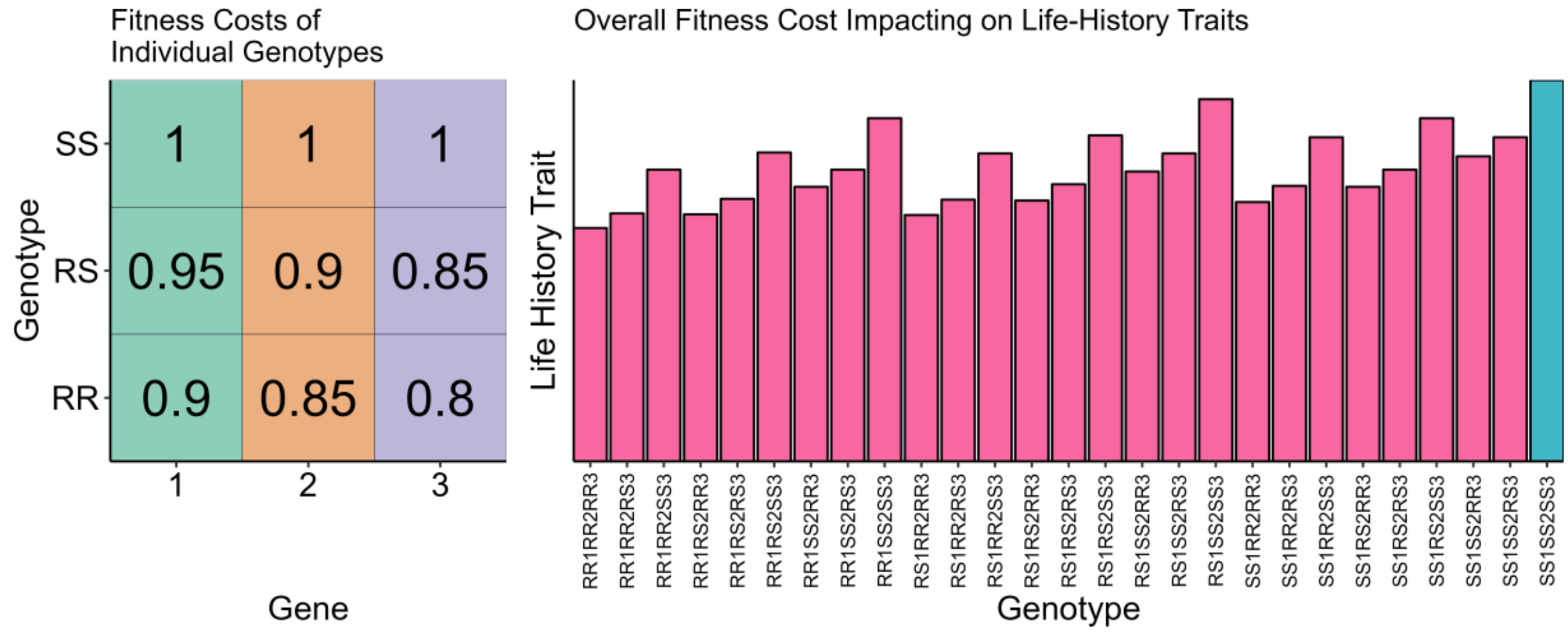

Fig P: Overall fitness cost in three gene model genotypes on life-history traits. The left-hand plot indicates the fitness costs associated with specific genes, with the values indicating the proportion of the fitness which is retained. These are then combined (detailed in equation S5.7.2), to give the overall fitness of the whole genotype for a specific life-history trait. Note, the values given in this plot are used only for illustrative purposes.

Table J: Parameter and State Variables in the two/three gene model update

Symbol	Description	Type	Value(s)	Rationale
$i_{larvicide:1}^{♀FF1FF2FF3}$	The survival to the larvicide of female larvae to the insecticide, which is set as the first larvicide, given the genotype.	Parameter	0-1	Covers full survival range. Note, not explored in the presented simulations.
$i_{larvicide:2}^{♀FF1FF2FF3}$	The survival to the larvicide of female larvae to the insecticide, which is set as the second larvicide, given the genotype.	Parameter	0-1	Covers full survival range. Note, not explored in the presented simulations.
$Y_{larvicide}$	Proportion of the larval habitats which receive the larvicide treatment.	Parameter	0-1	Covers full coverage range. Note, not explored in the presented simulations.
$i_{larvicide:1}^{♂MM1MM2MM3}$	The survival to the larvicide of male larvae to the insecticide, which is set as the first larvicide, given the genotype.	Parameter	0-1	Covers full survival range. Note, not explored in the presented simulations.
$i_{larvicide:2}^{♂MM1MM2MM3}$	The survival to the larvicide of male larvae to the insecticide, which is set as the second larvicide, given the genotype.	Parameter	0-1	Covers full survival range. Note, not explored in the presented simulations.
$E_{FF1FF2FF3}^{FF1FF2FF3\_MM}$	The proportion of female eggs with the genotype FF1FF2FF3 laid by female mosquitoes with the genotype FF1FF2FF3 which mated with male mosquitoes with the genotype MM1MM2MM3.	State Variable	Internally calculated.	N/A
$E_{MM1MM2MM3}^{FF1FF2FF3\_MM}$	The proportion of male eggs with the genotype MM1MM2MM3 laid by female mosquitoes with the genotype	State Variable	Internally calculated.	N/A

	FF1FF2FF3 which mated with male mosquitoes with the genotype MM1MM2MM3.			
G_{FF1}^{FF1MM1}	The proportion of eggs from the FF1MM1 cross which would give the target genotype FF1, as calculated using Hardy-Weinberg.	State Variable	Internally calculated.	N/A
G_{FF2}^{FF2MM2}	The proportion of eggs from the FF2MM2 cross which would give the target genotype FF2, as calculated using Hardy-Weinberg.	State Variable	Internally calculated.	N/A
G_{FF3}^{FF3MM3}	The proportion of eggs from the FF3MM3 cross which would give the target genotype FF3, as calculated using Hardy-Weinberg.	State Variable	Internally calculated.	N/A
G_{MM1}^{FF1MM1}	The proportion of eggs from the FF1MM1 cross which would give the target genotype MM1, as calculated using Hardy-Weinberg.	State Variable	Internally calculated.	N/A
G_{MM2}^{FF2MM2}	The proportion of eggs from the FF1MM1 cross which would give the target genotype MM2, as calculated using Hardy-Weinberg.	State Variable	Internally calculated.	N/A
G_{MM3}^{FF3MM3}	The proportion of eggs from the FF1MM1 cross which would give the target genotype MM3, as calculated using Hardy-Weinberg.	State Variable	Internally calculated.	N/A
$ITN_{♂}^{MM1MM2M}$	Daily probability an adult male mosquito with genotype MM1MM2MM3 encounters and survives contact with an ITN	State Variable	0-1	Covers full range of survival probabilities and is dependent on genotype.
$endophily_{♂}$	The daily probability a male mosquito goes indoors	Parameter	0-1	Covers full range of values
$\varepsilon_{ITN\|ITN}^{♂}$	The probability that when a male mosquito enters a house containing only an ITN it encounters the ITN.	Parameter	0-1	Covers full range of values
Y_{ITN}	The proportion of houses with ITNs only.	Parameter	0-1	Covers full range of values
$i_{ITN:1}^{♂MM1MM2MM3}$	The survival probability to the first insecticide in the ITN for male mosquitoes.	Parameter	0-1	Covers full range of values
$i_{ITN:2}^{♂MM1MM2MM3}$	The survival probability to the second insecticide in the ITN for male mosquitoes.	Parameter	0-1	Covers full range of values
$Y_{ITN+IRS}$	The proportion of houses with both ITNs and IRS	Parameter	0-1	Covers full range of values
$\varepsilon_{ITN\|ITN+IRS}^{♂}$	The probability that when a male mosquito enters a house containing both an ITN and IRS it encounters only the ITN.	Parameter	0-1	Covers full range of values
$\varepsilon_{IRS\|ITN+IRS}^{♂}$	The probability that when a male mosquito enters a house containing both an ITN and IRS it encounters only the IRS.	Parameter	0-1	Covers full range of values
$\varepsilon_{IRS\|IRS}^{♂}$	The probability that when a male mosquito enters a house containing only IRS it encounters only the IRS.	Parameter	0-1	Covers full range of values
Y_{IRS}	The proportion of houses with IRS only	Parameter	0-1	Covers full range of values

$i_{IRS:1}^{♂MM1MM2MM3}$	The survival probability to the first insecticide in the IRS for male mosquitoes.	Parameter	0-1	Covers full range of values
$i_{IRS:2}^{♂MM1MM2MM3}$	The survival probability to the first insecticide in the IRS for male mosquitoes.	Parameter	0-1	Covers full range of values
$Avoids_{♂}^{MM1MM}$	Daily probability an adult male mosquito avoids all insecticides.	State Variable	0-1	Covers full range of values
$i_{ITN:1}^{♀FF1FF2FF3}$	The survival probability to the first insecticide in the ITN for female mosquitoes.	Parameter	0-1	Covers full range of values
$i_{ITN:2}^{♀FF1FF2FF3}$	The survival probability to the second insecticide in the ITN for female mosquitoes.	Parameter	0-1	Covers full range of values
$endophily_{♀}$	Daily probability of female mosquitoes resting indoors.	Parameter	0-1	Covers full range of values
$genotype_{1i}$	Survival to insecticide i given by the first gene.	Parameter	0-1	Covers full range of values
$genotype_{2i}$	Survival to insecticide i given by the second gene.	Parameter	0-1	Covers full range of values
$genotype_{3i}$	Survival to insecticide i given by the third gene.	Parameter	0-1	Covers full range of values
$genotype_{1j}$	Survival to insecticide j given by the first gene.	Parameter	0-1	Covers full range of values
$genotype_{2j}$	Survival to insecticide j given by the second gene.	Parameter	0-1	Covers full range of values
$genotype_{3j}$	Survival to insecticide j given by the third gene.	Parameter	0-1	Covers full range of values
$genotype_{1k}$	Survival to insecticide k given by the first gene.	Parameter	0-1	Covers full range of values
$genotype_{2k}$	Survival to insecticide k given by the second gene.	Parameter	0-1	Covers full range of values
$genotype_{3k}$	Survival to insecticide k given by the third gene.	Parameter	0-1	Covers full range of values
$fitness_{b}$	Fitness of the genotype when considering life-history parameter b.	Parameter	0-1	Covers full range of values
$genotype_{1b}$	Fitness cost for life-history parameter b by the first gene.	Parameter	0-1	Covers full range of values
$genotype_{2b}$	Fitness cost for life-history parameter b by the second gene.	Parameter	0-1	Covers full range of values
$genotype_{3b}$	Fitness cost for life-history parameter b by the third gene.	Parameter	0-1	Covers full range of values

Appendix 6: Vectorial Capacity and Vector Control Loss (%)

Vectorial capacity is a commonly used metric to quantify the transmission potential of a mosquito population.

The standard equation for vectorial capacity[27] is:

$$V = \frac{ma^2}{-ln(p)} p^n$$

Equation S6.1

Where, m is the ratio of mosquitoes to humans (mosquito density); p is the probability of the mosquito surviving one day; a is the proportion of mosquitoes that feed on humans each day; and n is the number of days from infection to infectiousness in the mosquito (extrinsic incubation period).
The ratio of mosquitoes to humans (m) is calculated as:

$$m = \frac{M}{N}$$

Equation S6.2

Where M is the number of mosquitoes and N is the number of humans (by default we set $N = 1000$ unless otherwise stated).

To assess the impact that different resistance genotypes have on vectorial capacity, we can calculate the vectorial capacity separately for each genotype. This is especially important as each genotype would have different daily survival probabilities based on their ability to survive insecticide contact.

$$V = \sum V_{FF1FF2FF3}$$

Equation S6.3

We can also allow for SS, RS and RR to have different vector competencies (albeit not explored in the presented simulations). It has been found that resistant mosquitoes are less capable of harbouring the malaria parasite. Most models which have looked at the interaction between insecticide resistance and vector control have not included this as a potential beneficial side-product of insecticide resistance. For completeness we therefore include this option in our model. The vectorial capacity for a single genotype is therefore:

$$V_{FF1FF2FF3} = \frac{m_{FF1FF2FF3}(a_{FF1FF2FF3})^2}{-ln(p_{FF1FF2FF3})} p_{FF1FF2FF3}{}^{n_{FF1FF2FF3}}$$

Equation S6.4

The number of female mosquitoes of each genotype (FF1FF2FF3) at time point t is therefore:

$$M_{FF1FF2FF3} = A_{Hv\,(t)}^{FF1FF2FF3} + A_{Rv\,(t)}^{FF1FF2FF3} + A_{Ov\,(t)}^{FF1FF2FF3} + A_{H\,(t)}^{FF1FF2FF3} + A_{R\,(t)}^{FF1FF2FF3} + A_{O\,(t)}^{FFFF1FF2FF3}$$

Equation S6.5

$m_{FF1FF2FF3}$ is the the ratio of mosquitoes (with the specified genotype) to humans (mosquito genotype density)

$$m_{FF1FF2FF3} = \frac{M_{FF1FF2FF3}}{N}$$

Equation S6.6

$a_{FF1FF2FF3}$ is the human biting probability (and is therefore the proportion of mosquitoes that successfully feed on humans each day), that is it is the success probability of feeding, and is calculated as following:

$$a_{FF1FF2FF3} = \left(\left(\left(H\varepsilon_H^{♀} ZY f_{HI|net}^{♀FF1FF2FF3} i_i^{♀FF1FF2FF3}\right) + \left(H\varepsilon_H^{♀}(1-Y) f_{HI}^{♀FF1FF2FF3}\right) + \left(H\varepsilon_H^{♀}(1-Z) f_{HO}^{♀FF1FF2FF3}\right)\right) * \left(A_{Hv(t)}^{♀FF1FF2FF3} + \sum A_{H(t)}^{♀FF1FF2FF3\_MM1MM2MM3}\right)\right) / M_{FF1FF2FF3}$$

Equation S6.7

This is the proportion that transition from the host-seeking to resting, but only if they fed on the human and not on an alternative animal host.

To calculate the probability of an adult mosquito of genotype *FF1FF2FF3* surviving one day ($p_{FF1FF2FF3}$), we must average survival across all the behavioural states.

$$\bar{\rho}_H^{♀FF1FF2FF3} = \rho_H^{♀FF1FF2FF3} \left(\begin{array}{c} \left(H\varepsilon_H^{♀FF} \left(\left(ZY\, i_i^{♀FF1FF2FF3} \right) + \left(Z(1-Y) \right) + (1-Z) \right) \right) + \\ \left((1-H)\varepsilon_A^{♀} \right) + \\ \left(1 - \left(\left((1-H)\varepsilon_A^{♀} \right) + \left(H\varepsilon_H^{♀} \right) \right) \right) \end{array} \right)$$

Equation S6.8

$$Total\_A_{H(t)}^{♀FF1FF2FF3} = A_{Hv\,(t)}^{FF} + \sum A_{H(t)}^{♀FF1FF2FF3\_MM1MM2MM3}$$

Equation S6.9

$$Total\_A_{R(t)}^{♀FF} = A_{Rv\,(t)}^{FF} + A_{Ov\,(t)}^{FF} + \sum A_{R(t)}^{♀FF1FF2FF3\_MM1MM2MM3}$$

Equation S6.10

$$Total\_A_{O(t)}^{♀FF} = \left(A_{O(t)}^{♀FF\_SS} + A_{O(t)}^{♀FF\_RS} + A_{O(t)}^{♀FF\_RR} \right)$$

Equation S6.11

$$\bar{\rho}_R^{♀FF1FF2FF3} = \rho_R^{♀FF1FF2FF3} \left((endophily * irs.coverage * irs.survival) + (endophily * (1 - irs.coveraege) + (1 - endophily) \right)$$

Equation S6.13

$$\bar{\rho}_O^{♀FF1FF2FF3} = \rho_O^{♀FF1FF2FF3}$$

Equation S6.14

$$p_{FFFF1FF2FF3} = \frac{\left(\left(Total\_A_{H(t)}^{♀FF1FF2FF3} * \bar{\rho}_H^{♀FF1FF2FF3} \right) + \left(Total\_A_{R(t)}^{♀FF} * \bar{\rho}_R^{♀FF1FF2FF3} \right) + \left(Total\_A_{O(t)}^{♀FF} * \bar{\rho}_O^{♀FF1FF2FF3} \right) \right)}{M_{FF}}$$

Equation S6.15

Vector control loss (%) quantifies the reduction in the effectiveness of ITNs in controlling malaria transmission by vectors.

$$Vector\ Control\ Loss(\%) = \frac{(VC_{No\,Int}^{t} - VC_t^{Int}) - (VC_t^{No\,Int} - VC_t^{Res})}{(VC_t^{No\,Int} - VC_t^{Int})} * 100$$

Equation S6.16

$VC_t^{No\,Int}$ is the vectorial capacity at time t in base simulation, representing the maximum expected vectorial capacity in the absence of interventions. VC_t^{Int} is the vectorial capacity at time t for ITNs when IR is not included in the model, serving as an upper benchmark for potential control. VC_t^{Res} is the vectorial capacity at time t for ITNs when resistance is included. Both VC_t^{Int} and VC_t^{Res} are calculated using randomly drawn ITN coverage values for each parameter set.

Table K: Vectorial Capacity

Symbol	Description	Type	Value	Rational
V	The total vectorial capacity of the mosquito population.	State variable	N/A	N/A
$m_{FF1FF2FF3}$	the ratio of mosquitoes with the specified genotype to humans (mosquito genotype density)	State variable	N/A	N/A
$a_{FF1FF2FF3}$	The proportion of mosquitoes which feed on humans each day.	State variable	N/A	N/A
p	probability of a mosquito surviving one day	State variable	N/A	N/A
$n_{FF1FF2FF3}$	The extrinsic incubation period of the parasite in the mosquito with the specified genotype. This is the number of days from infection to infectiousness.	Parameter	10	Default assumed value.
$M_{FF1FF2FF3}$	The number of mosquitoes at with the specified genotype	State variable	N/A	N/A
N	The human population size.	Parameter	1000	Default Value to Scale the Vectorial Capacity
$V_{FF1FF2FF3}$	The vectorial capacity for a given genotype.	State variable	N/A	N/A
$m_{FF1FF2FF3}$	The ratio of mosquitoes to humans (mosquito density) for the specified genotype.	State variable	N/A	N/A
$a_{FF1FF2FF3}$	The proportion of mosquitoes which feed on humans each day given the mosquito genotype.	State variable	N/A	N/A
$p_{FFFF1FF2FF3}$	probability of a mosquito surviving one day for a given genotype.	State variable	N/A	N/A

Appendix 7: Additional Sensitivity Analysis

Appendix 7 includes additional sensitivity analysis for the simulations presented in the main manuscript text. Fig Q, Fig R and Fig S concern Simulation Set 1 (Impact of PYR-ITNs on IR and Malaria Vector Control) and Fig T concerns Simulation Set 2 (Impact of NGM-ITNs on IR and Malaria Vector Control). These additional sensitivity analysis help to increase the mechanistic processes underlying the impact of interventions on the evolution of insecticide resistance evolution and its subsequent impact on malaria vector control.

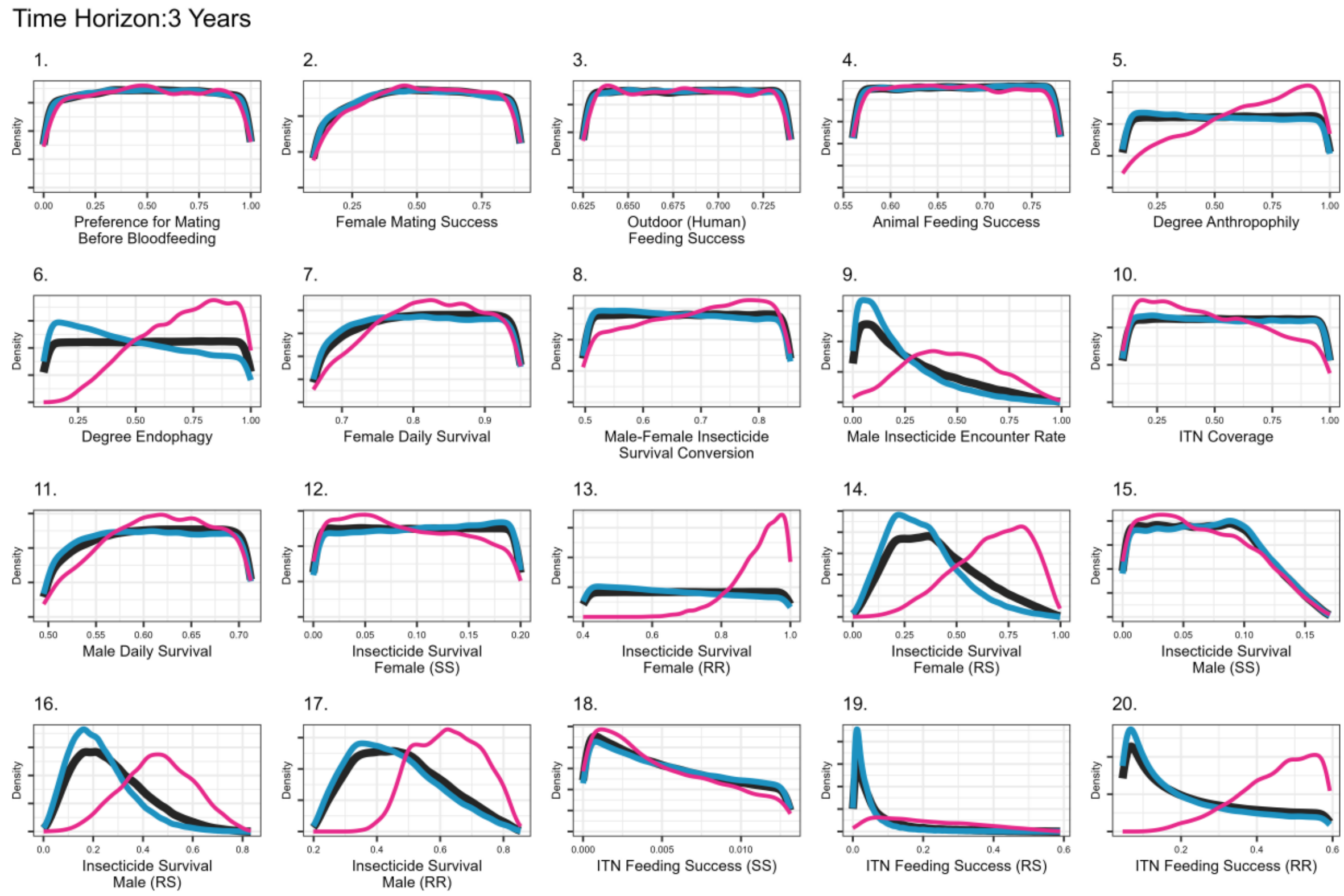

Fig Q: Distribution of the parameter values among all PYR-ITNs simulations, in simulations that strongly impact the vectorial capacity, or simulations that did not impact the vectorial after 3 years The black line is the distribution of all the parameter values used in the simulations (after exclusion of parameter sets which gave non-viable populations). The blue line is the distribution of the parameter values in simulations which had less than 1% reduction in control as a result of insecticide resistance at the 3-year time horizon, these are therefore simulations where resistance either did not reach high levels, or if resistance did reach high levels then this did not impact control. The pink line is the distribution of the parameter values where the vector control loss was greater than 50%. This was an arbitrary threshold and chosen to identify simulations where vector control was severely compromised by resistance. The blue line is the distribution of the parameter values where the vector vector control loss was less than 1% and is therefore the simulations where vector control loss was not noticeably compromised. The black line is therefore the expected distribution of the parameters, and if the lines (blue or pink) are below, then those values are under-represented (occur less frequently than expected), and if those lines are above, they are over-represented (occur more frequently than expected).

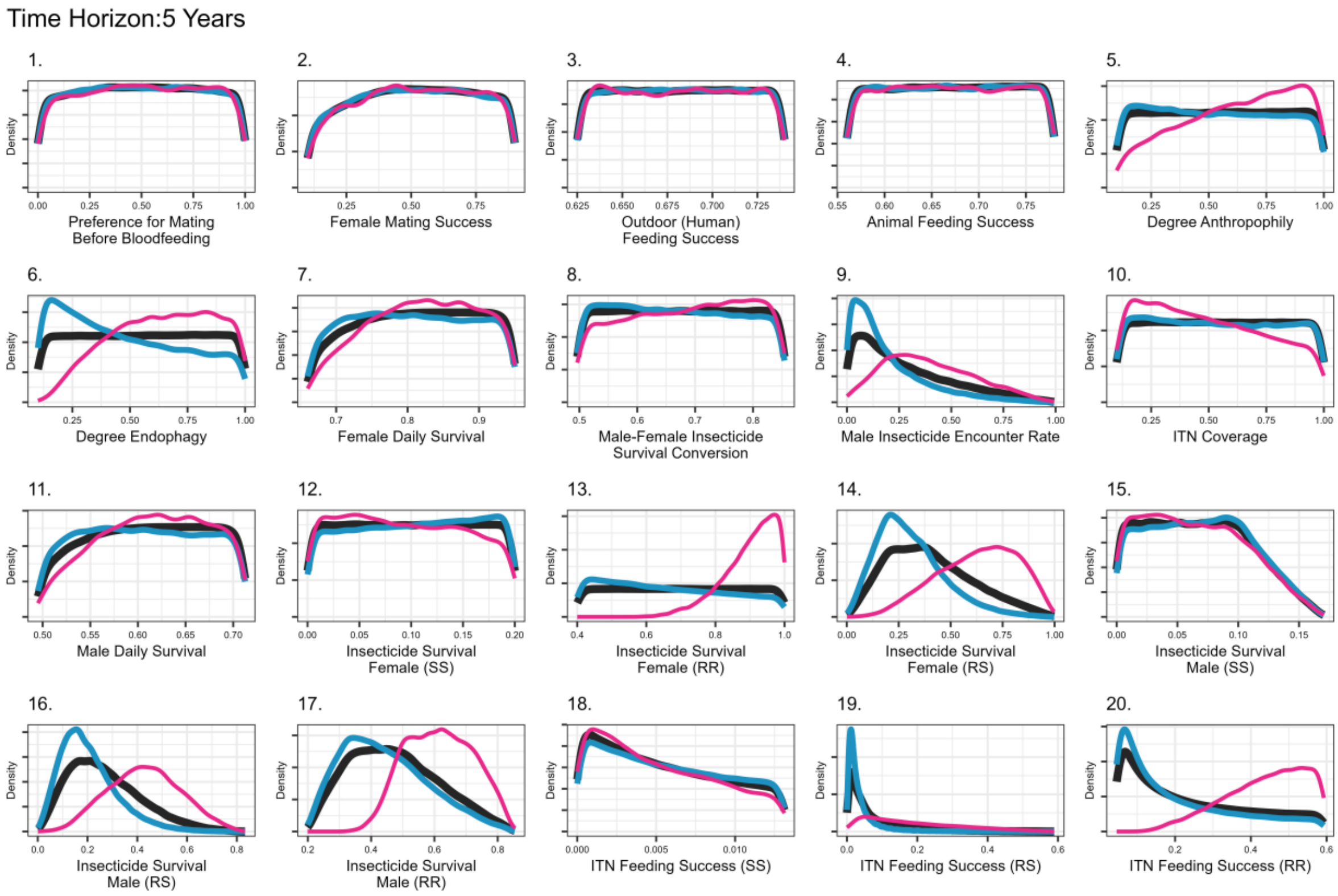

Fig R: Distribution of the parameter values among all PYR-ITNs simulations, in simulations that strongly impact the vectorial capacity, or simulations that did not impact the vectorial after 5 years. The black line is the distribution of all the parameter values used in the simulations (after exclusion of parameter sets which gave non-viable populations). The blue line is the distribution of the parameter values in simulations which had less than 1% reduction in control as a result of insecticide resistance at the 3-year time horizon, these are therefore simulations where resistance either did not reach high levels, or if resistance did reach high levels then this did not impact control. The pink line is the distribution of the parameter values where the vector control loss was greater than 50%. This was an arbitrary threshold and chosen to identify simulations where vector control was severely compromised by resistance. The black line is therefore the expected distribution of the parameters, and if the lines (blue or pink) are below, then those values are under-represented (occur less frequently than expected), and if those lines are above, they are over-represented (occur more frequently than expected).

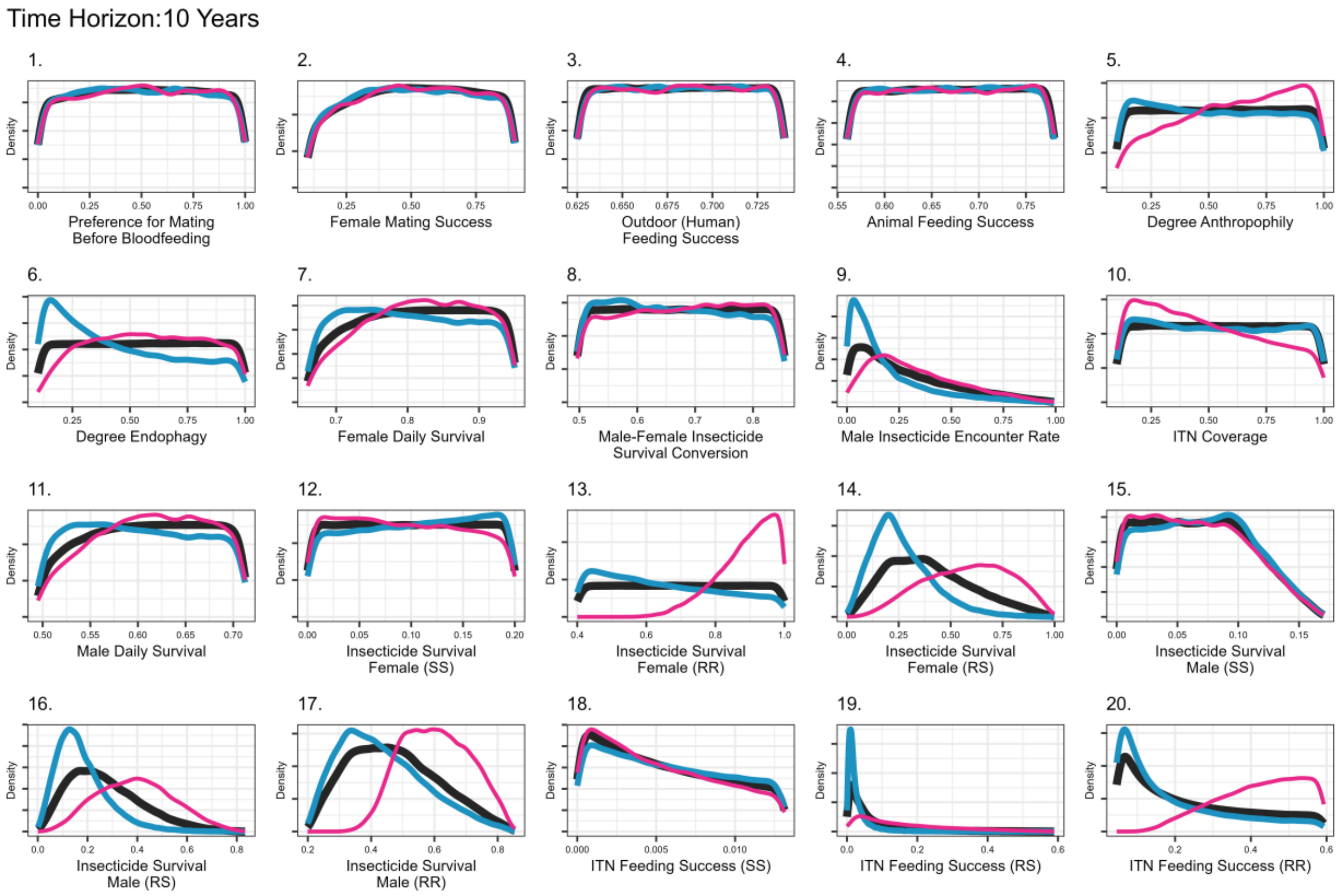

Fig S: Distribution of the parameter values among all PYR-ITNs simulations, in simulations that strongly impact the vectorial capacity, or simulations that did not impact the vectorial after 10 years The black line is the distribution of all the parameter values used in the simulations (after exclusion of parameter sets which gave non-viable populations). The blue line is the distribution of the parameter values in simulations which had less than 1% reduction in control as a result of insecticide resistance at the 10-year time horizon, these are therefore simulations where resistance either did not reach high levels, or if resistance did reach high levels then this did not impact control. The pink line is the distribution of the parameter values where the vector control loss was greater than 50%. This was an arbitrary threshold and chosen to identify simulations where vector control was severely compromised by resistance. The black line is therefore the expected distribution of the parameters, and if the lines (blue or pink) are below, then those values are under-represented (occur less frequently than expected), and if those lines are above, they are over-represented (occur more frequently than expected).

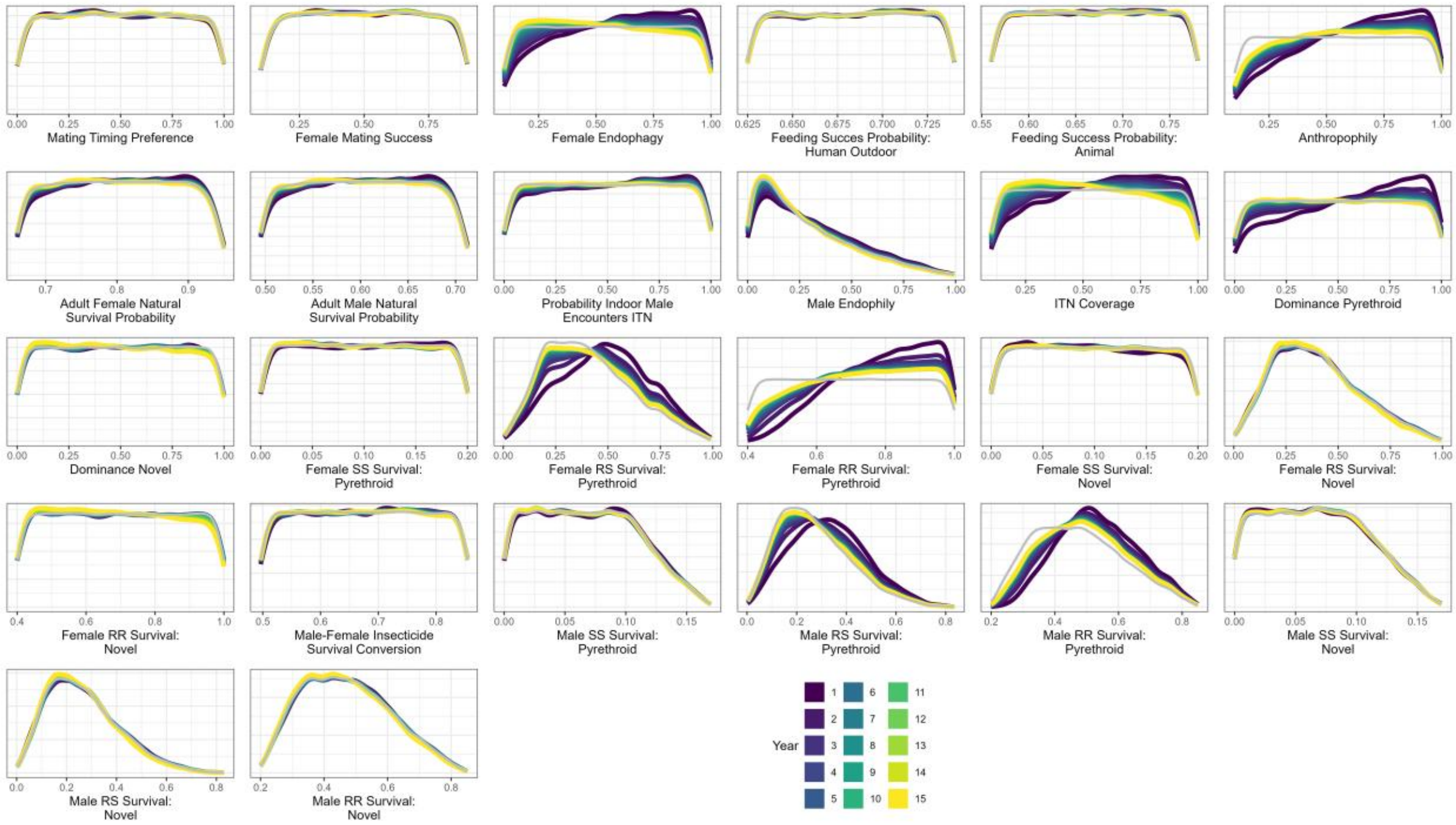

Fig T: Parameter Distributions for Simulations where the NGM-ITN at 70% relative coverage outperforms the PYR-ITN in reducing vectorial capacity. This figure compares the parameter distributions for all simulations (grey line). The grey line shows the distribution of parameter values across all simulations. Coloured lines, ranging from purple to yellow, represent subsets of simulations at each yearly time horizon where the NGM-ITN achieved greater suppression of vectorial capacity than the PYR-ITN. The NGM-ITN at relative coverage of 70% was chosen as the comparator because it highlights a moderate reduction in coverage. Coloured lines above the grey line indicate over-represented parameter values; lines below indicate under-representation.

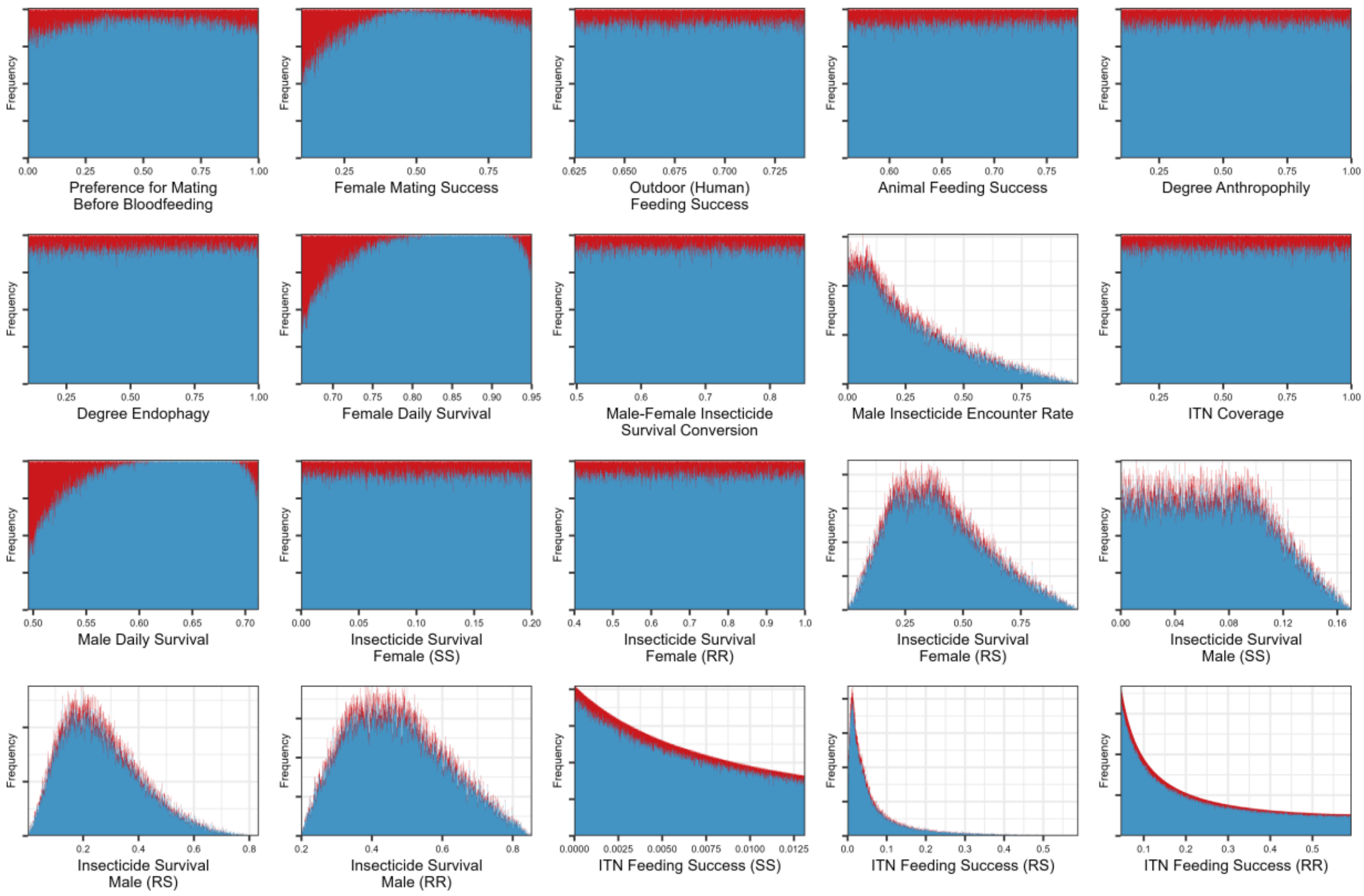

Fig U: Impact of Parameter Values on Population Viability. The use of Latin Hypercube Sampling means that some parameter permutations can give non-viable mosquito populations (either unrealistically high or unrealistically low), these were excluded from the analysis. The blue area indicates the viable simulations, and the red area indicates the non-viable simulations.

It is additionally possible to extract the expected age distribution from PopGEM (Fig V). This is a useful additional surrogate measure of the transmission potential of mosquito populations, as only older female mosquitoes are capable of transmitting *Plasmodium*, having been first infected then surviving through the extrinsic incubation period. Initiating simulations only with mated host-seeking females who we force to not lay eggs such that egg batch size = 0 but still they can still complete the oviposition behaviour to then start their next gonotrophic cycle. By performing short (30 day) simulations seeded with 100,000 mosquitoes (day 1) we can then calculate the number of each genotype surviving each day (Fig V). Additionally, this highlights how the mean population survival is dependent on the genotype composition, which is expected to change with mosquito age.

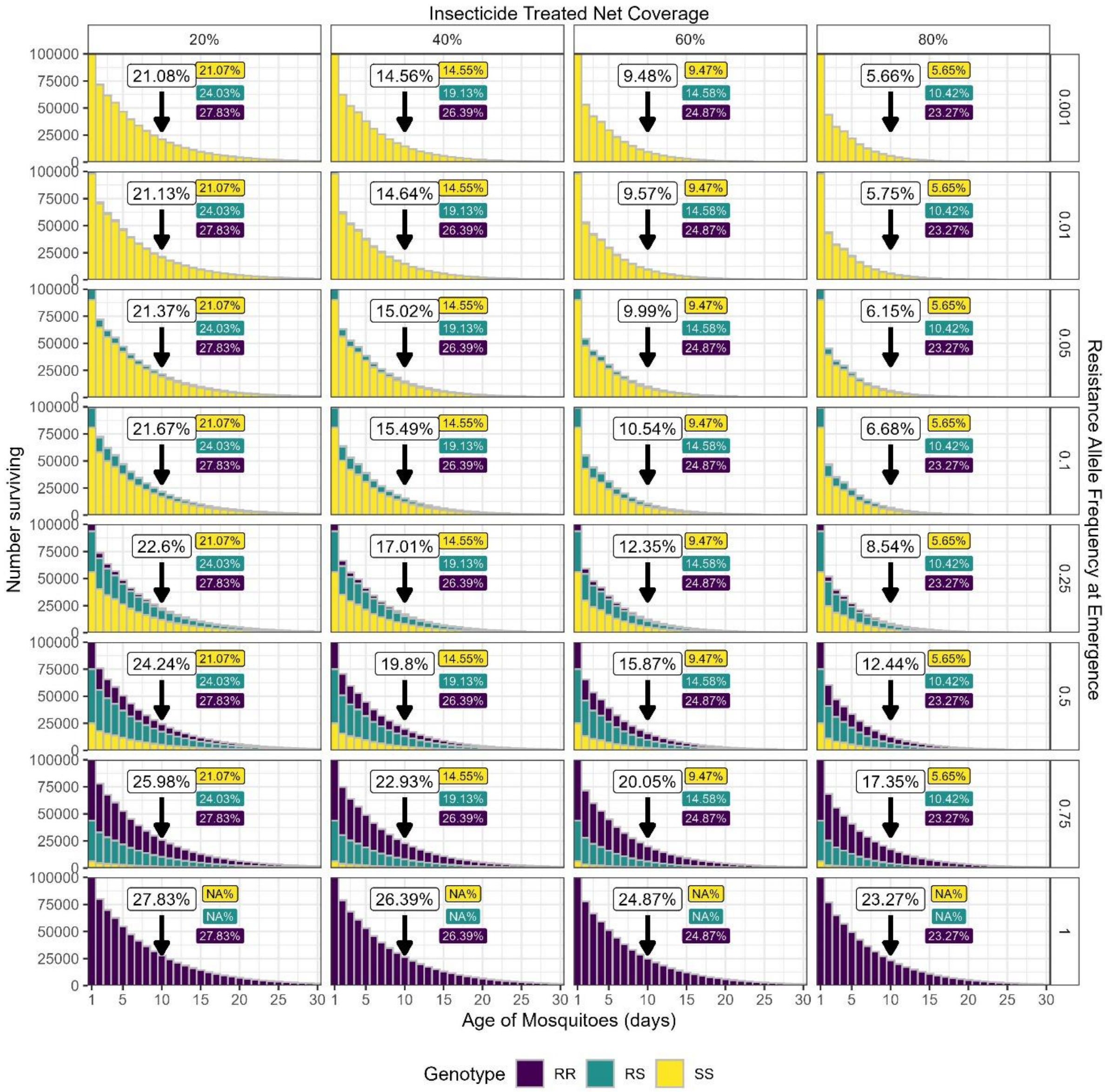

Fig V: Age distributions of female mosquitoes under different insecticide treated net coverages and with different resistance allele frequencies. The text labels in each panel indicate the percentage of female mosquitoes expected to survive to be 10 days old, where the colour labels are for the individual genotypes, and the white label is the overall percentage for the whole population. In this example set, anthropophagy = 0.8, endophagy = 0.8 and the natural survival probability was 0.9. The survival phenotypes to the ITN for the SS, RS and RR genotypes were 0.1, 0.5 and 0.9 respectively to the deployed ITN.

References

1 Chitnis N, Smith T, Steketee R. A mathematical model for the dynamics of malaria in mosquitoes feeding on a heterogeneous host population. *J Biol Dyn* 2008; **2**: 259–85.

2 White MT, Griffin JT, Churcher TS, Ferguson NM, Basáñez M-G, Ghani AC. Modelling the impact of vector control interventions on Anopheles gambiae population dynamics. *Parasites Vectors* 2011; **4**: 153.

3 Wickham H. testthat: Get Started with Testing. *The R Journal* 2011; **3**: 5.

4 Walker M, Robert MA, Childs LM. The importance of density dependence in juvenile mosquito development and survival: A model-based investigation. *Ecological Modelling* 2021; **440**: 109357.

5 Barbosa S, Kay K, Chitnis N, Hastings IM. Modelling the impact of insecticide-based control interventions on the evolution of insecticide resistance and disease transmission. *Parasites Vectors* 2018; **11**: 482.

6 Hancock PA, Godfray HCJ. Application of the lumped age-class technique to studying the dynamics of malaria-mosquito-human interactions. *Malar J* 2007; **6**: 98.

7 Clements AN. The Biology of Mosquitoes. In: Sensory Reception and Behaviour. Wallingford: Cabi, 1999.

8 Touré YT, Tripet F, Dolo G, Lanzaro GC. Frequency of multiple inseminations in field-collected anopheles gambiae females revealed by DNA analysis of transferred sperm. *Am J Trop Med Hyg* 2003; **68**: 1–5.

9 Charlwood JD, Pinto J, Sousa CA, Ferreira C, Petrarca V. 'A mate or a meal' – Pre-gravid behaviour of female Anopheles gambiae from the islands of São Tomé and Príncipe, West Africa. *Malaria Journal* 2003.

10 Takken W, Charlwood D, Lindsay SW. The behaviour of adult Anopheles gambiae, sub-Saharan Africa's principal malaria vector, and its relevance to malaria control: a review. *Malar J* 2024; **23**: 161.

11 Nash RK, Lambert B, N'Guessan R, *et al.* Systematic review of the entomological impact of insecticide-treated nets evaluated using experimental hut trials in Africa. *Current Research in Parasitology & Vector-Borne Diseases* 2021; **1**: 100047.

12 Champagne C, Lemant J, Assenga A, *et al.* Cascades of effectiveness of next-generation insecticide-treated nets against malaria, from entomological trials to real-life conditions. *Nat Commun* 2025; **16**: 11162.

13 Batume C, Akol AnneM, Mukwaya LG, Birungi J, Kayondo JK. Life-history attributes of juvenile Anopheles gambiae s.s. in central Uganda; implications for malaria control interventions. *Medical and Veterinary Entomology* 2022; **36**: 212:222.

14 Olayemi IK, Ande AT. Life table analysis of Anopheles gambiae (Diptera: Culicidae) in relation to malaria transmission. *J VECTOR BORNE DIS* 2009.

15 Christiansen-Jucht CD, Parham PE, Saddler A, Koella JC, Basáñez M-G. Larval and adult environmental temperatures influence the adult reproductive traits of Anopheles gambiae s.s. *Parasites Vectors* 2015; **8**: 456.

16 Müller GC. A novel window entry/exit trap for the study of endophilic behavior of mosquitoes. *Acta Trop* 2017; **167**: 137–41.

17 Oliva CF, Benedict MQ, Lempérière G, Gilles J. Laboratory selection for an accelerated mosquito sexual development rate. *Malar J* 2011; **10**: 135.

18 Yaro AS. The distribution of hatching time in Anopheles gambiae. *Malar J* 2006; **5**: 19.

19 Mordecai EA, Paaijmans KP, Johnson LR, *et al.* Optimal temperature for malaria transmission is dramatically lower than previously predicted. *Ecology Letters* 2013; **16**: 22–30.

20 Lines JD, Myamba J, Curtis CF. Experimental hut trials of permethrin-impregnated mosquito nets and eave curtains against malaria vectors in Tanzania. *Med Vet Entomol* 1987; **1**: 37–51.

21 Weetman D. Candidate-gene based GWAS identifies reproducible DNA markers for metabolic pyrethroid resistance from standing genetic variation in East African Anopheles gambiae. *Sci Rep* 2018; **8**: 2920.

22 Carnell R. lhs: Latin Hypercube Samples. 2022. https://CRAN.R-project.org/package=lhs.

23 Matthews J, Bethel A, Osei G. An overview of malarial Anopheles mosquito survival estimates in relation to methodology. *Parasit Vectors* 2020; **13**: 233.

24 Hobbs NP, Weetman D, Hastings IM. Insecticide resistance management strategies for public health control of mosquitoes exhibiting polygenic resistance: A comparison of sequences, rotations, and mixtures. *Evolutionary Applications* 2023; **16**: 936–59.

25 Hobbs NP, Hastings I. Simulating dynamic insecticide selection pressures for resistance management in mosquitoes assuming polygenic resistance. *PLoS Comput Biol* 2025; **21**: e1012944.

26 REX Consortium. Heterogeneity of selection and the evolution of resistance. *Trends Ecol Evol* 2013; **28**: 110–8.

27 Brady OJ, Godfray HCJ, Tatem AJ, *et al.* Vectorial capacity and vector control: reconsidering sensitivity to parameters for malaria elimination. *Trans R Soc Trop Med Hyg* 2016; **110**: 107–17.
